# Synthesis of 16 New Hybrids from Tetrahydropyrans Derivatives and Morita-Baylis-Hillman Adducts: In Vitro Screening against *Leishmania donovani*

**DOI:** 10.3390/molecules22020207

**Published:** 2017-01-30

**Authors:** Suervy Canuto de Oliveira Sousa, Juliana da Câmara Rocha, Tatjana de Souza Lima Keesen, Everton da Paz Silva, Priscilla Anne Castro de Assis, João Paulo Gomes de Oliveira, Saulo Luís Capim, Francisco José Seixas Xavier, Bruno Guimarães Marinho, Fábio Pedrosa Lins Silva, Claudio Gabriel Lima-Junior, Mário Luiz Araújo de Almeida Vasconcellos

**Affiliations:** 1 Laboratório de Síntese Orgânica Medicinal da Paraíba (LASOM-PB), Departamento de Química, Universidade Federal da Paraíba, Campus I, João Pessoa, PB 58059-900, Brazil; suervy@gmail.com (S.C.d.O.S.); notreve.bn@hotmail.com (E.d.P.S.); priscilla.cassis@gmail.com (P.A.C.d.A.); jp_0108@hotmail.com (J.P.G.d.O.); seicxhas@hotmail.com (F.J.S.X.); pedrosalinssilva@gmail.com (F.P.L.S.); 2 Departamento de Biotecnologia, Universidade Federal da Paraíba, Campus I, João Pessoa, PB 58059-900, Brazil ; ju_jurocha@hotmail.com (J.d.C.R.); tat.keesen@cbiotec.ufpb.br (T.d.S.L.K.); 3 Unidade Acadêmica de Saúde, Centro de Educação e Saúde, Universidade Federal de Campina Grande, Campus Cuité, Cuité, PB 58175-000, Brazil; 4 Instituto Federal de Educação, Ciência e Tecnologia da Bahia, Campus Catu, Barão de Camaçari, Catu, BA 48110-000, Brazil; sauloquimico@hotmail.com; 5 Programa de Pós-Graduação em Ciências Fisiológicas and Laboratório de Farmacologia, Departamento Ciências Fisiológicas, Instituto de Ciências e Saúde, Universidade Federal Rural do Rio de Janeiro Seropédica, RJ 23890-000, Brazil; bruno.marinho78@hotmail.com

**Keywords:** antileishmanial Morita-Baylis-Hillman adducts, opioids tetrahydropyrans derivatives, molecular hybridization, *Leishmania donovani*

## Abstract

Leishmaniases are a group of neglected tropical diseases (NTDs) caused by protozoan parasites from >20 *Leishmania* species. Visceral leishmaniasis (VL), also known as kala-aza, is the most severe form of leishmaniasis, usually fatal in the absence of treatment in 95% of cases. The Morita-Baylis-Hillman adducts (MBHAs) are being explored as drug candidates against several diseases, one of them being leishmaniasis. We present here the design, synthesis and in vitro screening against *Leishmania donovani* of sixteen new molecular hybrids from analgesic/anti-inflammatory tetrahydropyrans derivatives and Morita-Baylis-Hillman adducts. First, acrylates were synthesized from analgesic/anti-inflammatory tetrahydropyrans using acrylic acid under TsOH as a catalyst (70%–75% yields). After the 16 new MBHAs were prepared in moderate to good yields (60%–95%) promoted by microwave irradiation or low temperature (0 °C) in protic and aprotic medium. The hybrids were evaluated in vitro on the promastigote stage of *Leishmania donovani* by determining their inhibitory concentrations 50% (IC_50_), 50% hemolysis concentration (HC_50_), selectivity index (HC_50_/IC_50_,), and comparing to Amphotericin B, chosen as the anti-leishmanial reference drug. The hybrid which presents the bromine atom in its chemical structure presents high leishmanicide activity and the high selectivity index in red blood cells (SI_rb_ > 180.19), compared with the highly-toxic reference drug (SI_rb_ = 33.05), indicating that the bromine hybrid is a promising compound for further biological studies.

## 1. Introduction

Neglected tropical diseases (NTDs) are those that occur primarily in poor countries. Drugs to treat these diseases are few, and usually lead to severe side effects, since the buying power of these populations is very low, not being attractive to global pharmaceutical industries to invest money on the development of drugs for these diseases [1]. Leishmaniases are a group of NTDs caused by protozoan parasites from >20 *Leishmania* species [2]. Visceral leishmaniasis (VL), also known as kala-azar, is the most severe form of leishmaniasis, usually fatal in the absence of treatment in over 95% of cases [3]. *Leishmania donovani* is one of the major species of the genus *Leishmania* that causes VL, affecting more than 100 million people worldwide, with 500,000 new cases and more than 50,000 deaths each year [4]. Due to inexistence of vaccines for humans and that the available chemotherapy is toxic and expensive, research aiming to obtain new efficient drugs is of great urgency.

**Scheme 1:**
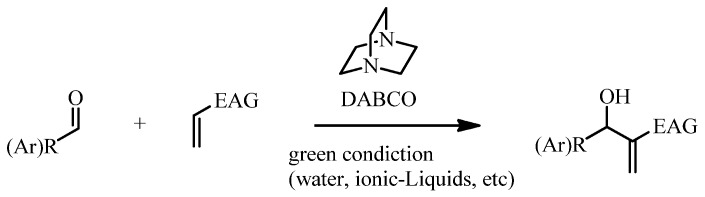
General Morita-Baylis-Hillman reaction. EAG = electron attractor group. Ar = aryl or heteroaryl, R = alkyl or H.

The Morita-Baylis-Hillman adducts (MBHAs) are being explored as drug candidates against several diseases, one of them being leishmaniasis [5]. These compounds are prepared from the Morita-Baylis-Hillman reaction [6,7] in green conditions from aldehydes (among other electrophiles) or alkenes connected to electron attractor groups (EAG, like methyl acrylate, acrylonitrile, and others) under basic catalysis (DABCO being the most common base, Scheme 1).

**Figure 1:**
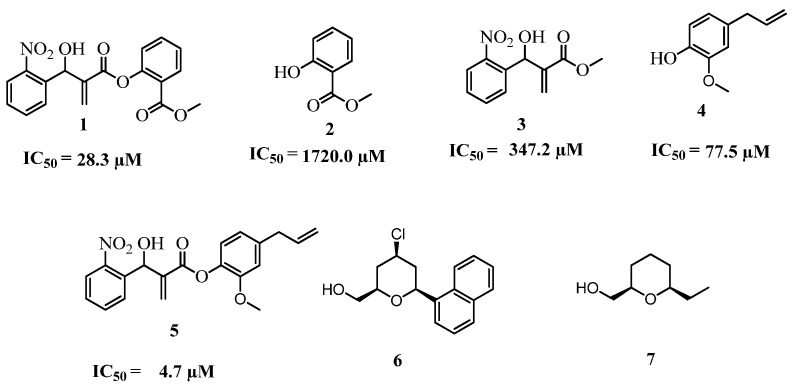
The leishmanicidal activity of hybrids **1** and **5** and their precursors. The analgesic/anti-inflammatory **6** and **7**.

Molecular hybridization is a useful tool in the design of new drug prototypes [8]. It was reported by us that the hybridization between an analgesic/anti-inflammatory methyl salicylate (**2**) and the MBHA (**3**) resulting in the development of a chalcone-like compound (**1**) that is more active as a leishmanicide than corresponding precursors [9]. In 2016 we also described the synthesis of a new hybrid (**5**) from an analgesic and anti-inflammatory eugenol (**4**) that presents a stronger leishmanicidal activity than compound **1** [10] (Figure 1).

In a different line of research from our group, synthesis and in vivo experiments were described that demonstrate that tetrahydropyran derivative **6** is very efficient and is a non-toxic analgesic/anti-inflammatory [11,12]. It was published the synthesis of (±)-*cis,cis*-**6** [13,14] and recently the anti-hyperalgesic effect of is associated with NO/cGMP/KATP pathway participation and the κ-opioid receptor [15]. Additionally, it was recently discovered that the tetrahydropyran derivative (±)-*cis-***7** [16,17] presents an analgesic effect in the same order of magnitude (μmol·kg^−1^) than the analogous **6** (Figure 1) [16].

**Figure 2:**
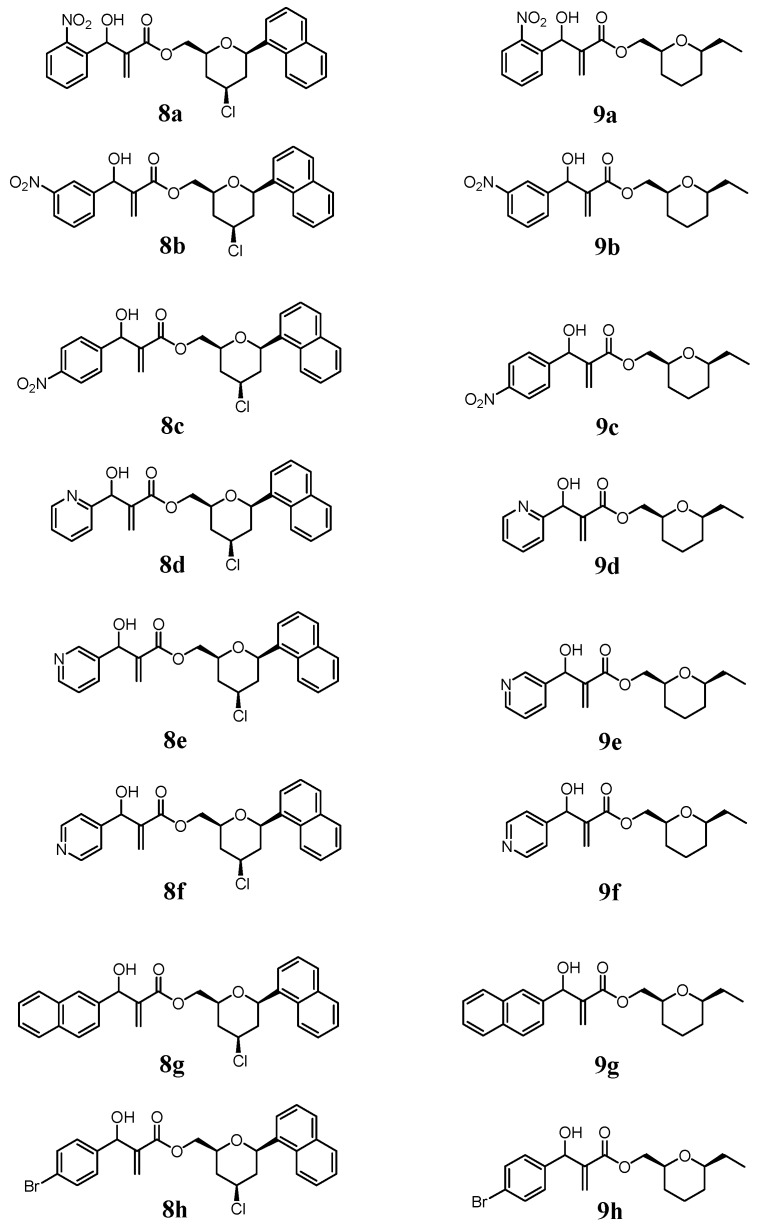
The synthetized hybrids **8a**–**8h** and **9a**–**9h**.

In connection with our interest in the discovery of new Morita-Baylis-Hillman adducts with promising leishmanicidal and other bioactivities [5,18,19,20,21], we present here the design, synthesis, and in vitro screening against *Leishmania donovani* of sixteen new molecular hybrids (**8a**–**h** and **9a**–**h**, Figure 2). It is important to note here that our design is based on the fact that inhibitors of opioid receptors participate in the mechanism of action against leishmaniasis interfering with the immune system, not only participating in the function of the immune cells, but also modulating innate and acquired immune responses [22]. 

## 2. Results and Discussion

### 2.1. Chemistry

**Scheme 2:**
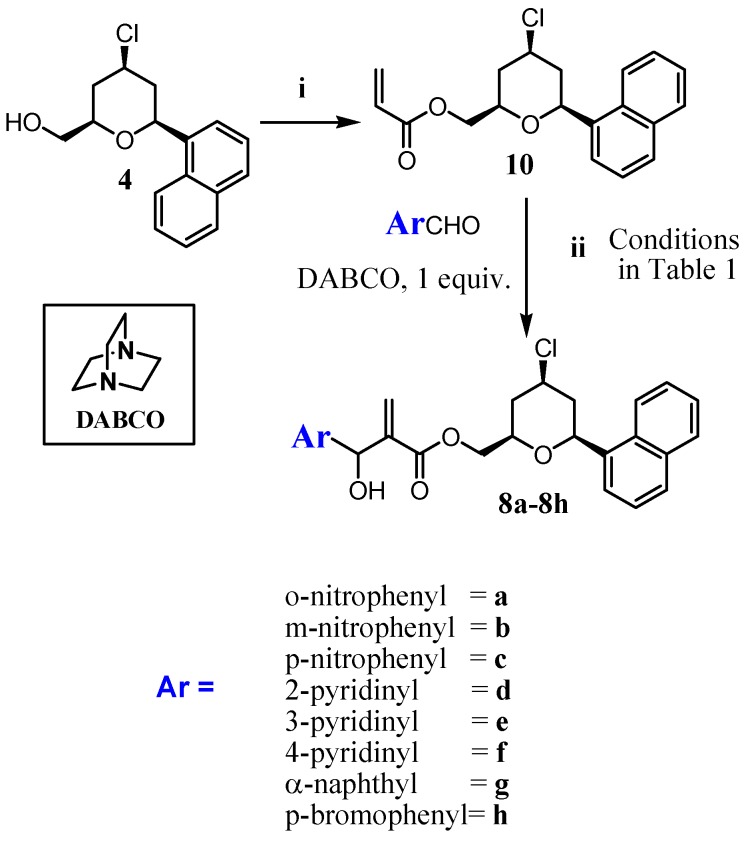
(i) Acrylic acid (2 equiv.), TsOH (20 mol%), anhydrous CH_2_Cl_2_, 50 °C, two days, 70% yield. (ii) MBHR between **10** and ArCHO (conditions and yields in Table 1).

**Scheme 3:**
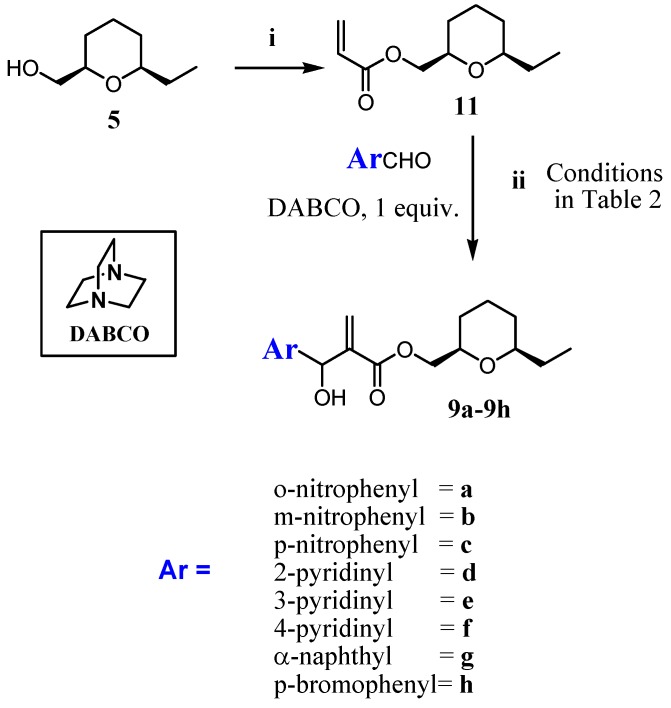
(i) Acrylic acid (2 equiv.), TsOH (20 mol%), anhydrous CH_2_Cl_2_, 50 °C, two days, 75% yield. (ii) MBHR between **11** and ArCHO (conditions and yields in Table 2).

Compounds **6** and **7** were prepared in the same way as described in the literature [11,13]. Synthesis of new hybrids **8a**–**8h** (spectra, see in Supplementary Materials) were made via the Morita-Baylis-Hillman reaction (MBHR) between acrylate **10** and aromatic aldehydes ArCHO (Scheme 2) under DABCO as a promoter (conditions and yields are described in Table 1). Acrylate **10** was synthetized from **6** using acrylic acid under TsOH as a catalyst (70%, Scheme 2). In the same way, the new acrylate **11** was prepared from **7** (Scheme 3) with 75% yield. The conditions and yields of the MBH reaction between **11** and ArCHO, to prepare the hybrids **9a**–**9h** (spectra, see in Supplementary Materials) are presented in Table 2. 

**Scheme 4:**
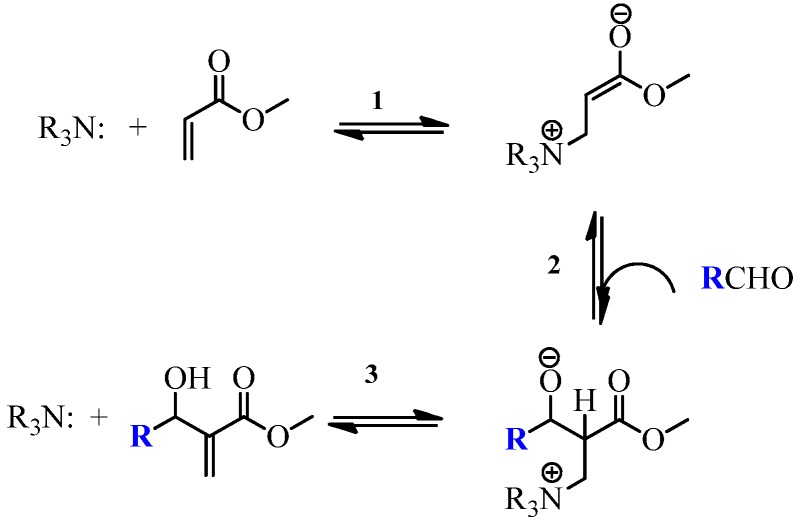
The general proposal mechanism of MBHR.

The general proposal mechanism of MBHR is presented in Scheme 4. 

However, the slow step of the mechanism and the transition state structure has been the main source of controversy [7]. To date, the slow step has been proposed to change between the aldol step (step 2) and the elimination step (step 3). In fact, depending on various parameters, such as the Michael acceptor used as the reagent, solvents, addition or not of phenolic additives, temperature (may be reversible or not), many different catalysts, etc. [7], MBHR is an exquisite reaction and each reaction must have its experimental parameters carefully optimized as an individual problem. However, some facts are incontestable: the MBHR between methyl acrylate reacting with *p*-bromobenzaldehyde is reversible at elevated temperatures [21]; several MBHR are faster at 0 °C than at room temperature [23,24]; and the type of solvent (aprotic or protic) [25,26] and the phenol or naphthol additives modifies the reactions rate. We can see in Table 1 (Entries 1, 2, 4–8, 10 ) and Table 2 (Entries 1–6, 8 and 10) that it used t-buOH/H_2_O at 80 °C under microwave irradiation as the first experimental attempt [21]. When this condition was not efficient, low temperatures were used (0 °C) in DMF/water as the solvent mixture and used phenol as the additive, increasing yields (Entries 7, 9 and 11, Table 2). Addition of phenol as an additive with t-buOH/H_2_O at 80 °C under microwave irradiation also improves the rate and yields (compare entries 6 and 7, Table 2). These delicate results can be interpreted based on the experimental studies on the MBHR described recently by Plata and Singleton [27]. Enthalpy and entropy of activations, contributions in ΔG^≠^ were measured on the methyl acrylate and *p*-nitrobenzaldehyde reaction in methanol as a solvent and DABCO as a promoter, proving the pivotal importance of entropic parameters in the slow step of this reaction.

### 2.2. Biology

**Figure 3:**
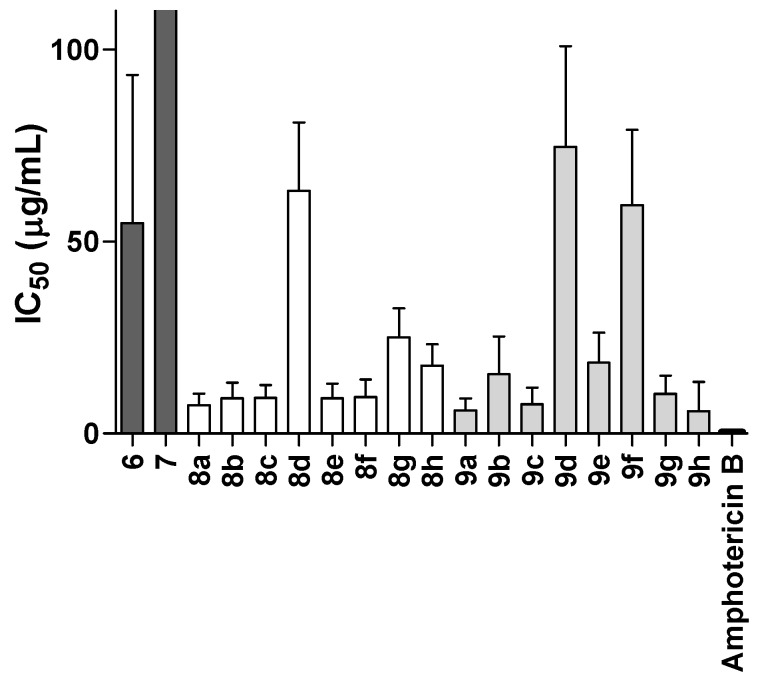
In vitro biological results.

The hybrids **8a**–**8h** and **9a**–**9h** were evaluated in vitro on the promastigote stage of *Leishmania donovani* by determining their inhibitory concentrations 50% (IC_50_), 50% hemolysis concentration (HC_50_), selectivity index (HC_50_/IC_50_,), and comparing to Amphotericin B, chosen as the anti-leishmanial reference drug (Table 3 and Figure 3).

The experimental results are shown in Table 3 and deserve comment here. Precursors **6** and **7** had their leishmanicidal activity evaluated (data not shown). Precursor **6** was shown to be effective against *L. donovani* (IC_50_ = 172.19 μM), whereas the precursor tetrahydropyrans (THP )with the ethyl portion (**7**) has no leishmanicida activity (IC_50_ > 2775.46 μM). Knowing this, the proposal of the work was the molecular hybridization of these precursors with MBHAs presenting anti-*leishmania* activities already reported, aiming to increase the leishmanicidal activity. Initially hybrids **8a**–**8h**, demonstrated a higher antiparasitic activity against *L. donovani* compared to precursor **6**; leishmanicidal activity with IC_50_ values ranging from 14.74 μM to 141.67 μM. As can be seen in Table 3, among hybrids **8a**–**8h**, compounds **8a**, **8b**, **8c**, **8e**, and **8f** showed similar anti-*leishmania* activity against the promastigote forms of *L. donovani*, but **8a** presented a lesser cytotoxic effect (HC_50_ >831,38 μM) and the highest selectivity index (S.I. _rb_ > 56.40), showing the most promise of the **8a**–**8h** hybrids.

The improvement in leishmanicidal activity was also observed for the hybrids originating from precursor **7** that did not show antiparasitic activity (IC_50_ > 2775.46 μM), but with the hybrids **9a**–**9h** presented higher antiparasitic activities against *L. donovani*. The variation of leishmanicidal activities of hybrids **9a**–**9h** IC_50_ values ranged from 5.81 μM to 240.27 μM. Hybrids **9a**, **9c**, and **9h** were the ones that presented greater tendencies of antiparasitic activities, presenting IC_50_ values of 16.15 μM, 20.13 μM, and 5.81 μM, respectively. Additionally, values of HC_50_ were >1000 μM. It should be noted that the high selectivity index of **9h** (SI_rb_ > 180.19) compared with the reference drug that presents high toxicity (SI_rb_ = 33.05). The hybrid **9h** presents the bromine atom in its chemical structure. In previous works described by our research group we highlight the association between leishmanicidal activity and the presence of bromine atoms as substituents in the aromatic moiety [9,18,19,20,28]. Among the works we highlight two examples. In 2007 we reported a significant increase in leishmanicidal activity in promastigote forms of *L. amazonensis* when the benzene moiety (49.3 μM) was changed to *p*-bromobenzyl (12.5 μM) in MBHAs. Furthermore, in these two compounds there was no toxicity measured by the release of lactate dehydrogenase into macrophages (% LDH = 0%) [18]. In 2016 we reported that the homodimer of MBHAs with a bromine atom as a substituent in the aromatic moiety showed very high anti-*leishmania* activity on *L. donovani* (0.50 μM) almost 400 times more active than the corresponding monomer and 1.24 times more potent than Amphotericin B (0.62 μM). Moreover, the selectivity index of the homodimer was very high (SI_rb_ > 400), far better than Amphotericin B (SI_rb_ = 18.73) [28]. These results demonstrate that the hybridization reactions were pivotal for the increase of leishmanicidal action and maintaining the absence of toxicity in macrophages and red blood cells of the hybrids, indicating that **9h** is a promising compound for further biological studies.

## 3. Materials and Methods 

### 3.1. Chemistry

#### 3.1.1. General

All commercially available reagents and solvent were obtained from the commercial providers from Sigma-Aldrich^®^ (St. Louis, MO, USA) and used without further purification, except the opioids tetrahydropyrans **6** and **7**, which have previously been synthesized. Reactions were monitored by TLC (thin layer chromatography) using silica gel 60 UV254 Macherey-Nagel pre-coated silica gel plates (Macherey-Nagel, Bethlehem, PA, USA); detection was by means of a UV lamp. Flash column chromatography was performed on 300–400 mesh silica gel. Organic layers were dried over anhydrous MgSO_4_ or Na_2_SO_4_ prior to evaporation on a rotary evaporator. ^1^H and ^13^C nuclear magnetic resonance (NMR) spectra were recorded using Varian Mercury Spectra AC 20 spectrometer (500 MHz for ^1^H, 125 MHz for ^13^C). Chemical shifts were reported relative to internal tetramethylsilane (δ 0.00 ppm) for ^1^H, and CDCl_3_ (δ 77.0 ppm) for ^13^C (Varian, Palo Alto, CA, USA). HRMS was obtained using Q-TOF quadrupole/orthogonal spectrometry (Waters, Milford, MA) in negative or positive mode. Analyses of low-resolution mass spectrometry were obtained via a gas chromatograph coupled with a mass spectrometer, model GCMS–QP2010 from Shimadzu. Infrared spectra (FTIR) data were recorded in a spectrophotometer, model IR Prestige–21 from Shimadzu (Shimadzu, Kyoto, Japan), using potassium bromide (KBr) pellets. Reactions requiring microwave irradiation were performed in a CEM® microwave reactor, Discover model, with a BenchMate system temperature monitor with an infrared sensor (Gilroy, CA, USA). 

#### 3.1.2. Procedure for the Synthesis of Acrylate **10** and **11**

Tetrahydropyrans **6** or **7** (10 mmol), acrylic acid (20 mmol), p-toluensulfonic acid (20 mol%), and 80 mL anhydrous CH_2_Cl_2_ were mixed in a 125 mL flask under a reflux system at 50 °C for two days. The reaction mixture was neutralized with sodium bicarbonate aqueous solution, washed with salt water until pH = 7, and dried by anhydrous sodium sulfate. The mixture was purified by column chromatography, performed using silica gel (230–400 mesh). Acrylates **10** and **11** were obtained with yields of 70% and 75%, respectively.

#### 3.1.3. General Procedure for the Synthesis of MBHA (**8a**–**8h**) and (**9a**–**9h**)

Reactions were carried out using the acrylates **10** or **11** (0.5 mmol), corresponding aromatic aldehydes (1.0 mmol), and 3 mL of appropriate solvent in the presence of DABCO, with catalytic promotion and under microwave irradiation (MW), or subjected to a temperature of 0 °C when a reaction improvement was necessary. After that, the solvent was removed and the reaction media was directly filtered through silica gel, using hexane/ethyl acetate (7:3) as eluent. The reaction products were concentrated under reduced pressure (yields are shown in Table 1 and Table 2).

*Acrylic acid 4-chloro-6-naphthalen-1-yl-tetrahydro-pyran-2-ylmethyl ester* (**10**): FTIR (KBr) cm^−1^: 541, 777, 794, 954, 1062, 1188, 1298, 1406, 1633, 1718, 2944. RMN ^1^H (500 MHz; CDCl_3_) δ 1.835 (dd, 1H, *J =* 15 Hz); 2.08 (dd, 1H, *J =* 15 Hz); 2.33 (dt, 1H, *J* = 10 Hz, *J =* 5 Hz); 2.60 (dt, 1H, *J =* 10 Hz, *J =* 5 Hz); 3.99 (m, 1H); 4.31 (m, 1H); 5.1 (d, 1H, *J =* 10 Hz); 5.85 (dd, 1H, *J =* 10 Hz, *J =* 2.5 Hz); 6.16 (dd, 1H, *J =* 15 Hz, *J =* 10 Hz); 6.45 (dd, 1H, *J =* 15 Hz, *J =* 2.5 Hz); 7.48 (m, 3H); 7.62 (d, 1H, *J =* 5 Hz); 7.80 (d, 1H, *J =* 5 Hz); 7.86 (d, 1H, 10 Hz); 7.97 (d, 1H, *J =* 10 Hz). RMN ^13^C (125 MHz; CDCl_3_): δ 38.57; 42.70; 55.16; 66.38; 75.11; 75.82; 123.02; 123.31; 125.46; 125.55; 126.34; 128.06; 128.53; 129.93; 130.31; 131.32; 133.78; 136.04; 165.94. LC-MS-IT-TOF calculated for C_19_H_20_ClO_3_: 331.1095 [M + H]^+^; found: 331.1097.

*Acrylic acid 6-ethyl-tetrahydro-pyran-2-ylmethyl ester* (**11**): FTIR (KBr) cm^−1^: 455, 645, 800, 894, 955, 1230, 1411, 1540, 1643, 1704, 2849, 2926. RMN ^1^H (500 MHz; CDCl_3_) δ 0.94 (t, 3H, *J* = 10 Hz); δ 1.51 (m, 2H); δ 1.61 (m, 2H); δ 2.16 (m, 2H); δ 3.25 (m, 1H); δ 3.62 (m, 1H); δ 4.16 (dd, 1H, *J =* 15 Hz, 5 Hz); δ 4.21 (dd, 1H, *J =* 10 Hz, 5 Hz); δ 5.85 (dd, 1H, *J =* 10 Hz, 2.5 Hz); δ 6.16 (dd, 1H, *J =* 15 Hz, 10 Hz); δ 6.44 (dd, 1H, *J =* 15 Hz, 2.5 Hz). RMN ^13^C (125 MHz; CDCl_3_) δ 9.78; 28.63; 38.76; 41.76; 55.34; 66.43; 74.29; 78.24; 128.13; 131.19; 165.95. LC-MS-IT-TOF calculated for C_11_H_19_O_3_: 199.1329 [M + H]^+^; found: 199.1330.

*2-[Hydroxy-(2-nitro-phenyl)-methyl]-acrylic acid 4-chloro-6-naphthalen-1-yl-tetrahydro- pyran-2- ylmethyl ester* (**8a**): FTIR (KBr) cm^−1^: 781, 1051, 1136, 1159, 1265, 1301, 1348, 1446, 1525, 1720, 2927, 2960, 3448. RMN ^1^H (500 MHz; CDCl_3_) δ 1.14 (dd, 1H, *J* = 25 Hz, 10 Hz); δ 1.60 (m. 1H); 1.91 (ddd, 1H, *J* = 30 Hz, 25 Hz, 10 Hz); δ 2.08 (m, 1H); δ 2.49 (d, 1H, *J* = 10 Hz); δ 2.83 (d, 1H); δ 3.8 (m, 1H); δ 4.18 (m, 3H); δ 4.97 (dd, 1H, *J =* = 15 Hz, 10 Hz); δ 5.76 (d, 1H, *J* = 15 Hz); δ 6.08 (d, 1H, *J* = 10 Hz); δ 6.39 (d, 1H, *J* = 15 Hz); δ 7.18 (dt, 1H, *J* = 5 Hz); δ 7.41 (m, 5H); δ 7.55 (dd, 1H, *J* = 20 Hz, 10 Hz); δ 7.72 (m, 2H); δ 7.80 (m, 1H); δ 7.87 (t, 1H, *J* = 10 Hz). RMN ^13^C (125 MHz; CDCl_3_) δ 38.29; 38.47; 55.00; 66.79; 67.49; 74.80; 75.30; 122.84; 122.96; 123.33; 124.53; 124.62; 125.60; 126.23; 127.24; 128.43; 128.56; 128.79; 129.00; 133.47; 136.09; 137.17; 140.37; 140.45; 148.40; 165.50. LC-MS-IT-TOF calculated for C_26_H_24_ClNO_6_Na: 504.1184 [M + H]^+^; found: 504.1188.

*2-[Hydroxy-(3-nitro-phenyl)-methyl]-acrylic acid 4-chloro-6-naphthalen-1-yl-tetrahydro- pyran-2- yl-methyl ester (**8b**):* FTIR (KBr) cm^−1^: 781, 802, 1053, 1087, 1159, 1348, 1527, 1714, 2854, 2924, 3427, RMN ^1^H (500 MHz; CDCl_3_) δ 1.64 (q, 1H, *J* = 10 Hz); δ 1.95 (ddd, 1H, *J* = 25 Hz, 15 Hz, 5 Hz); δ 2.14 (m, 1H); δ 2.50 (d, 1H, *J* = 10 Hz); δ 3.85 (m, 1H); δ 4.20 (m, 3H); δ 5.00 (d, 1H, *J* = 10 Hz); δ 5.52 (d, 1H, *J* = 5 Hz); δ 5.85 (d, 1H, *J* = 10 Hz); δ 6.39 (s, 1H); δ 7.29 (dt, 1H, *J* = 10 Hz, 7.5 Hz); δ 7.41 (m, 3H); δ 7.50 (dd, 1H, *J* = 10 Hz, 5 Hz); δ 7.57 (dd, 1H, *J* = 20 Hz, 10 Hz); δ 7.73 (d, 1H, *J* = 10 Hz); δ 7.80 (dd, 1H, *J* = 5 Hz, 2.5 Hz); δ 7.88 (d, 1H, *J* = 5 Hz); δ 7.96 (m, 1H); δ 8.15 (d, 1H, *J* = 15 Hz). RMN ^13^C (125 MHz; CDCl_3_) δ 38.34; 42.71; 54.88; 66.84; 72.51; 74.86; 75.83; 121.49; 122.79; 123.26; 125.50; 125.66; 126.26; 127.88; 128.66; 129.06; 129.34; 130.26; 132.65; 133.81; 135.90; 140.89; 143.59; 148.31; 165.48. LC-MS-IT-TOF calculated for C_26_H_24_ClNO_6_Na: 504.1184 [M + H]^+^; found: 504.1188.

*2-[Hydroxy-(4-nitro-phenyl)-methyl]-acrylic acid 4-chloro-6-naphthalen-1-yl-tetrahydro-pyran-2- ylmethyl ester* (**8c**): FTIR (KBr) cm^−1^: 781, 854, 1053, 1159, 1263, 1346, 1521, 1604, 1720, 2926, 2960, 3450. RMN ^1^H (500 MHz; CDCl_3_) δ 1.16 (d, 1H, *J* = 10 Hz); δ 1.64. (q, 1H, *J* = 10 Hz); δ 1.96 (m, 1H); δ 2.14 (d, 1H, *J* = 15 Hz); δ 2.51 (d, 1H, *J* = 0 Hz); δ 3.84 (m, 1H); δ 4.20 (m, 3H); δ 5.00 (d, 1H, *J* = 10 Hz); δ 5.52 (d, 1H, *J* = 10 Hz); δ 5.83 (d, 1H, *J* = 10 Hz); δ 6.38 (s, 1H); δ 7.42 (m, 5H); δ 7.51 (d, 1H, *J* = 5 Hz); δ 7.75 (d, 1H, *J* = 5 Hz); δ 7.80 (m, 1H); δ 7.89 (m, 1H); δ 7.99 (dd, 2H, *J* = 10 Hz, 5 Hz). RMN ^13^C (125 MHz; CDCl_3_) δ 18.95; 38.30; 42.63; 54.87; 66.79; 72.51; 74.88; 75.84; 122.87; 123.27; 123.60; 125.49; 125.68; 126.28; 127.32; 127.79; 127.88; 128.73; 129.10; 130.28; 133.83; 133.85; 140.94; 147.44; 148.57; 165.46. LC-MS-IT-TOF calculated for C_26_H_24_ClNO_6_Na: 504.1184 [M + H]^+^; found: 504.1186.

*2-(Hydroxy-pyridin-2-yl-methyl)-acrylic acid 4-chloro-6-naphthalen-1-yl-tetrahydro-pyran-2-yl methyl ester (**8d**):* FTIR (KBr) cm^−1^: 781, 800, 958, 1064, 1159, 1247, 1375, 1438, 1593, 1720, 2926, 2958, 3057, 3394. RMN ^1^H (500 MHz; CDCl_3_) δ 1.18 (s, 1H); δ 1.66 (dd, 2H, *J =* 30 Hz, 10 Hz); δ 1.14 (d, 1H, *J =* 15 Hz); δ 2.50 (dt, 1H, *J =* 20 Hz, 5 Hz); δ 4.20 (m, 4H); δ 5.61 (d, 1H, *J =* 10 Hz); δ 6.00 (d, 1H, *J* = 10 Hz); δ 6.40 (d, 1H, *J =* 5 Hz); δ 6.97 (t, 1H, *J =* 10 Hz); δ 7.41 (m, 6H); δ 7.74 (d, 2H, *J =* 10 Hz); δ 7.79 (m, 1H); δ 7.90 (m, 1H). RMN ^13^C (125 MHz; CDCl_3_) δ 29.71; 38.41; 42.81; 55.04; 66.65; 71.37; 71.57; 74.94; 75.77; 121.86; 122.98; 123.40; 125.49; 125.67; 126.33; 128.53; 128.96; 130.25; 133.79; 136.17; 128.23; 141.01; 146.68; 158.86; 165.49. LC-MS-IT-TOF calculated for C_25_H_24_ClNO_4_Na: 438.1467 [M + H]^+^; found: 438.1469.

*2-(Hydroxy-pyridin-3-yl-methyl)-acrylic acid 4-chloro-6-naphthalen-1-yl-tetrahydro-pyran-2-yl methyl ester* (**8e**): FTIR (KBr) cm^−1^: 713, 781, 800, 848, 1058, 1157, 1261, 1375, 1425, 1597, 1624, 1720, 2856, 2960, 3055, 3294. RMN ^1^H (500 MHz; CDCl_3_) δ 1.26 (m, 1H); δ 1.71 (qua, 1H, *J* = 20 Hz); δ 2.04 (m, 1H); δ 2.19 (m, 1H); δ 2.58 (dt, 1H, *J* = 25 Hz, 5 Hz); δ 3.90 (t, 1H, *J* = 15 Hz); δ 4.24 (m, 3H); δ 5.06 (dd, 1H, *J* = 20 Hz); δ 5.57 (s, 1H); δ 5.96 (d, 1H, *J* = 5 Hz); δ 6.45 (s, 1H); δ 7.12 (m, 1H); δ 7.48 (m, 3H); δ 7.58 (d, 1H); δ 7.66 (t, 1H, *J* = 20 Hz); δ 7.82 (m, 2H); δ 7.96 (d, 1H, *J* = 15 Hz); δ 8.40 (m, 1H); δ 8.55 (s, 1H). RMN ^13^C (125 MHz; CDCl_3_) δ 38.57; 42.88; 55.12; 66.87; 71.10; 75.07; 75.97; 123.09; 123.46; 123.62; 125.68; 125.81; 126.40; 127.33; 128.76; 129.19; 130.44; 133.96; 134.74; 136.14; 137.36; 141.44; 148.38; 148.90; 165.67. LC-MS-IT-TOF calculated for C_25_H_24_ClNO_4_Na: 438.1467 [M + H]^+^; found: 438.1470.

*2-(Hydroxy-pyridin-4-yl-methyl)-acrylic acid 4-chloro-6-naphthalen-1-yl-tetrahydro-pyran-2-yl methyl ester* (**8f**): FTIR (KBr) cm^−1^: 781, 800, 1066, 1178, 1244, 1373, 1448, 1598, 1734, 2854, 2958, 3053, 3332. RMN ^1^H (500 MHz; CDCl_3_) Despite the spectrum does not provide enough resolution to demonstrate the integration of the peaks we can observe the presence of the olefinic and carbinolic hydrogens signals characterizing the C-C bond formation in Morita-Baylis-Hillman reaction step. δ 5.42 (d, 1H, *J =* 5 Hz); δ 5.83 (d, 1H, 10 Hz); δ 6.38 (s, 1H). RMN ^13^C (125 MHz; CDCl_3_) δ 13.24; 24.06; 38.16; 41.31; 42.66; 48.44; 54.83; 65.68; 67.21; 74.72; 75.72; 121.36; 122.75; 122.83; 123.31; 125.53; 125.67; 126.26; 128.53; 128.62; 129.07; 150.85; 159.84. LC-MS-IT-TOF calculated for C_25_H_24_ClNO_4_Na: 438.1467 [M + H]^+^; found: 438.1470.

*2-(Hydroxy-naphthalen-2-yl-methyl)-acrylic acid 4-chloro-6-naphthalen-1-yl-tetrahydro-pyran -2-ylmethyl ester* (**8g**): FTIR (KBr) cm^−1^: 474, 553, 746, 772, 956, 1034, 1139, 1252, 1507, 1621, 1708, 2847, 2926, 2952, 3057, 3425. RMN ^1^H (500 MHz; CDCl_3_) δ 1.62 (ddd, 1H, *J* = 20 Hz, 10 Hz, 5 Hz); δ 1.90 (qui, 1H, *J* = 10 Hz); δ 2.07 (m, 1H); δ 2.47 (d, 1H, *J* = 15 Hz); δ 2.82 (d, 1H); δ 3.77 (m, 1H); δ 4.10 (m, 1H); δ 4.15 (dd, 1H, *J* = 10 Hz, 5 Hz); δ 4.18 (m, 1H); δ 4.95 (m, 1H); δ 5.66 (s, 1H); δ 5.87 (d, 1H, *J* = 2.5 Hz); δ 6.39 (s, 1H); δ 7.39 (m, 7H); δ 7.71 (m, 6H); δ 7.80 (d, 1H, *J* = 5 Hz); δ 7.86 (d, 1H, *J* = 10 Hz). RMN ^13^C (125 MHz; CDCl_3_) δ 29.72; 38.39; 42.65; 54.95; 66.57; 73.30; 74.95; 75.80; 122.93; 122.98; 123.96; 124.57; 125.50; 125.60; 126.07; 126.19; 127.03; 127.68; 128.09; 128.29; 128.55; 129.00; 130.26; 133.02; 133.20; 133.80; 135.98; 138.60; 141.70; 165.95. LC-MS-IT-TOF calculated for C_30_H_27_ClO_4_Na: 509.1490 [M + H]^+^; found: 509.1492.

*2-[(4-Bromo-phenyl)-hydroxy-methyl]-acrylic acid 4-chloro-6-naphthalen-1-yl-tetrahydro-pyran -2-ylmethyl ester* (**8h**): FTIR (KBr) cm^−1^: 550, 728, 771, 797, 950, 1009, 1034, 1137, 1265, 1299, 1401, 1503, 1630, 1707, 2856, 2925, 3435. RMN ^1^H (500 MHz; CDCl_3_) δ 1.63 (dd, 1H, *J* 30 Hz, 10 Hz); δ 1.93 (m, 1H); δ 2.09 (dd, 1H, *J =* 20 Hz, 5 Hz); δ 2.48 (dt, 1H, *J* 15 Hz, 5 Hz); δ 3.76 (qui, 1H); δ 4.15 (m, 3H); δ 4.95 (d, 1H, *J =* 10 Hz); δ 5.37 (s, 1H); δ 5.76 (d, 1H, *J =* 10 Hz); δ 6.30 (s, 1H); δ 7.90 (t, 2H, *J =* 10 Hz); δ 7.27 (m, 2H); δ 7.39 (m, 3H); δ 7.49 (d, 1H, *J =* 10 Hz); δ 7.72 (d, 1H, *J =* 10 Hz); δ 7.79 (m, 1H); δ 7.86 (d, 1H, *J =* 15 Hz) RMN ^13^C (125 MHz; CDCl_3_) δ 38.35; 42.70; 54.99; 66.64; 72.58; 74.94; 75.84; 121.82; 122.98; 123.33; 125.53; 125.67; 126.27; 126.97; 127.07; 128.36; 128.65; 129.07; 130.30; 131.57; 133.83; 135.99; 140.35; 141.48; 165.75. LC-MS-IT-TOF calculated for C_26_H_24_BrClO_4_Na: 537.0439 [M + H]^+^; found: 537.0442.

*2-[Hydroxy-(2-nitro-phenyl)-methyl]-acrylic acid 6-ethyl-tetrahydro-pyran-2-ylmethyl ester* (**9a**): FTIR (KBr) cm^−1^: 752, 786, 1037, 1145, 1267, 1350, 1527, 1633, 1720, 2875, 2933, 2962, 3448. RMN ^1^H (500 MHz; CDCl_3_) δ 0.84 (t, 2H, *J* 15 Hz); δ 1.42 (m, 6H); δ 2.06 (m, 1H); δ 3.14 (m, 1H); δ 3.90 (m, 1H); δ 4.04 (d, 2H, *J =* 15 Hz); δ 5.68 (d, 1H, *J* = 15 Hz); δ 6.11 (d, 1H, *J* = 10 Hz); δ 6.36 (s, 1H); δ 7.40 (t, 1H, *J* = 15 Hz); δ 7.58 (t, 1H, *J* = 15 Hz); δ 7.65 (m, 1H); δ 7.89 (ddd, 1H, *J* = 15 Hz, 10 Hz, 2.5 Hz). RMN ^13^C (125 MHz; CDCl_3_) δ 9.71; 28.53; 38.58; 41.66; 55.10; 66.73; 67.63; 73.97; 78.22; 124.58; 127.16; 128.87; 133.49; 136.04; 140.53; 148.34; 165.41. LC-MS-IT-TOF calculated for C_18_H_24_NO_6_: 350.1598 [M + H]^+^; found: 350.1602.

*2-[Hydroxy-(3-nitro-phenyl)-methyl]-acrylic acid 6-ethyl-tetrahydro-pyran-2-ylmethyl ester* (**9b**): FTIR (KBr) cm^−1^: 740, 812, 962, 1047, 1147, 1350, 1529, 1624, 1658, 1722, 2873, 2931, 2962, 3427. RMN ^1^H (500 MHz; CDCl_3_) δ 0.85 (t, 3H, *J* = 15 Hz); δ 1.43 (m, 4H); δ 2.07 (m, 1H); δ 3.17 (m, 1H); δ 3.49 (m, 1H); δ 3.91 (m, 1H); δ 4.06 (m, 2H); δ 5.56 (s, 1H); δ 5.84 (d, 1H, *J* = 10 Hz); δ 6.39 (d, 1H, *J* = 10 Hz); δ 7.46 (t, 1H, *J* = 20 Hz); δ 7.66 (t, 1H, *J* = 10 Hz); δ 8.07 (d, 1H, *J* = 15 Hz); δ 8.19 (s, 1H). RMN ^13^C (125 MHz; CDCl_3_) δ 9.70; 28.55; 38.39; 41.58; 54.92; 66.79; 72.65; 73.89; 78.20; 121.46; 122.78; 127.88; 129.34; 132.52; 140.81; 143.70; 148.33; 165.32. LC-MS-IT-TOF calculated for C_18_H_24_NO_6_: 350.1598 [M + H]^+^; found: 350.16012.

*2-[Hydroxy-(4-nitro-phenyl)-methyl]-acrylic acid 6-ethyl-tetrahydro-pyran-2-ylmethyl ester* (**9c**): FTIR (KBr) cm^−1^: 754, 829, 854, 962, 1047, 1147, 1265, 1348, 1521, 1604, 1631, 1720, 2875, 2935, 2964, 3462. RMN ^1^H (500 MHz; CDCl_3_) δ 0.92 (t, 3H, *J =* 10 Hz); δ 1.51 (m, 5H); δ 2.05 (m, 1H); δ 2.15 (dt, 1H, *J =* 15 Hz, 5 Hz); δ 3.23 (m, 1H); δ 3.55 (m, 1H); δ 3.985 (m, 1H); δ 4.15 (d, 2H, *J =* 10 Hz); δ 5.64 (d, 1H, *J =* 5 Hz); δ 5.90 (d, 2H, *J =* 15 Hz); δ 6.46 (d, 1H, *J =* 5 Hz); δ 7.58 (dd, 2H, *J =* 10 Hz, 2.5 Hz); δ 8.20 (d, 2H, *J =* 10 Hz). RMN ^13^C (125 MHz; CDCl_3_) δ 9.71; 28.57; 38.38; 41.59; 54.93; 66.78; 72.65; 73.98; 78.23; 123.62; 127.38; 127.79; 127.94; 140.91; 147.46; 148.61; 148.73; 165.37. LC-MS-IT-TOF calculated for C_18_H_24_NO_6_: 350.1598 [M + H]^+^; found: 350.15995.

*2-(Hydroxy-pyridin-2-yl-methyl)-acrylic acid 6-ethyl-tetrahydro-pyran-2-ylmethyl ester* (**9d**): FTIR (KBr) cm^−1^: 754, 960, 1006, 1045, 1062, 1147, 1267, 1382, 1436, 1593, 1635, 1720, 2875, 2933, 2962, 3392. RMN ^1^H (500 MHz; CDCl_3_) δ 0.84 (t, 3H, *J =* 10 Hz); δ 1.44 (m, 5H); δ 1.95 (m, 1H); δ 2.07 (dt, 1H, *J =* 15 Hz, 2.5 Hz); δ 3.14 (qui, 1H, *J =* 7.5 Hz); δ 3.46 (t, 1H, *J =* 7.5 Hz); δ 3.91 (qui, 1H, *J =* 5 Hz, 10 Hz); δ 4.06 (m, 2H); δ 5.55 (s, 1H); δ 5.93 (s, 1H); δ 6.34 (s, 1H); δ 7.16 (t, 1H, *J =* 7.5 Hz); δ 7.37 (t, 1H, *J =* 10 Hz); δ 7.61 (t, 1H, *J =* 10 Hz); δ 8.48 (d, 1H, *J =* 5 Hz). RMN ^13^C (125 MHz; CDCl_3_) δ 9.72; 28.57; 38.65; 41.68; 55.19; 66.53; 72.20; 74.08; 78.15; 121.23; 122.71; 127.50; 136.87; 141.53; 148.22; 159.36; 165.68. LC-MS-IT-TOF calculated for C_17_H_24_NO_4_: 306.1700 [M + H]^+^; found: 306.17010.

*2-(Hydroxy-pyridin-3-yl-methyl)-acrylic acid 6-ethyl-tetrahydro-pyran-2-ylmethyl ester (***9e***):* FTIR (KBr) cm^−1^: 713, 758, 813, 848, 960, 1058, 1147, 1263, 1327, 1377, 1425, 1460, 1624, 1633, 1720, 2875, 2933, 2962, 3332. RMN ^1^H (500 MHz; CDCl_3_) δ 0.84 (t, 3H, *J* = 15 Hz); 1.43 (m, 4H); 1.94 (m, 1H); 2.07 (dt, 1H, *J* = 25 Hz, 5 Hz); 3.15 (m, 1H); 3.45 (m, 1H); 3.91 (m, 1H); 4.06 (m, 2H); 5.53 (s, 1H); 5.88 (d, 1H, *J* = 10 Hz); 6.37 (s, 1H); 7.20 (t, 1H, *J* = 10 Hz); 7.66 (dd, 1H, *J* = 15 Hz, 2.5 Hz); 8.40 (dd, 1H, *J* = 10 Hz, 2.5 Hz); 8.48 (s, 1H). RMN ^13^C (125 MHz; CDCl_3_) δ 9.72; 28.55; 38.49; 41.59; 55.02; 66.65; 70.93; 73.98; 78.16; 123.40; 127.00; 134.44; 137.14; 141.41; 148.34; 148.83; 165.40. LC-MS-IT-TOF calculated for C_17_H_24_NO_4_: 306.1700 [M + H]^+^; found: 306.17011.

*2-(Hydroxy-pyridin-4-yl-methyl)-acrylic acid 6-ethyl-tetrahydro-pyran-2-ylmethyl* ester (**9f**): FTIR (KBr) cm^−1^: 758, 873, 1041, 1089, 1149, 1259, 1377, 1409, 1458, 1602, 1635, 1735, 2852, 2927, 2962, 3392. RMN ^1^H (500 MHz; CDCl_3_) Despite the spectrum does not provide enough resolution to demonstrate the integration of the peaks we can observe the presence of the olefinic and carbinolic hydrogens signals characterizing the C-C bond formation in Morita-Baylis-Hillman reaction step. δ 5.48 (d, 1H, *J =* 5 Hz); δ 5.84 (d, 1H, *J =* 10 Hz); δ 6.38 (d, 1H, *J =* 5 Hz). RMN ^13^C (125 MHz; CDCl_3_) δ 9.81; 13.25; 28.58; 38.28; 41.61; 48.43; 54.99; 55.59; 65.60; 67.31; 73.91; 78.23; 121.47; 141.95; 150.95; 169.81. LC-MS-IT-TOF calculated for C_17_H_24_NO_4_: 306.1700 [M + H]^+^; found: 306.17006.

*2-(Hydroxy-naphthalen-2-yl-methyl)-acrylic acid 6-ethyl-tetrahydro-pyran-2-ylmethyl ester* (**9g**): FTIR (KBr) cm^−1^: 752, 819, 962, 1045, 1147, 1269, 1382, 1462, 1631, 1658, 1718, 2852, 2927, 2962, 3427. RMN ^1^H (500 MHz; CDCl_3_) δ 0.81 (t, 3H, *J* = 5 Hz); δ 1.35 (m, 3H); δ 1.45 (m, 1H); δ 1.87 (m, 1H); δ 1.99 (d, 1H); δ 3.06 (m, 1H); δ 3.37 (m, 1H); δ 3.78 (m, 1H); δ 4.01 (m, 2H); δ 5.635 (d, 1H); δ 5.81 (d, 1H); δ 6.35 (d, 1H); δ 7.39 (m, 3H); δ 7.75 (dd, 4H, *J* = 10 Hz); RMN ^13^C (125 MHz; CDCl_3_) δ 9.78; 28.59; 38.52; 41.63; 55.10; 66.58; 73.34; 74.06; 78.18; 124.60; 125.51; 126.10; 126.23; 127.02; 127.73; 128.12; 128.28; 133.05; 133.24; 138.68; 141.80; 165.89. LC-MS-IT-TOF calculated for C_22_H_26_O_4_Na: 377.1723 [M + H]^+^; found: 377.17258.

*2-[(4-Bromo-phenyl)-hydroxy-methyl]-acrylic acid 6-ethyl-tetrahydro-pyran-2-ylmethyl ester* (**9h**): FTIR (KBr) cm^−1^: 759, 817, 875, 962, 1014, 1045, 1070, 1147, 1271, 1382, 1404, 1460, 1631, 1656, 1716, 2852, 2927, 2962, 2448. RMN ^1^H (500 MHz; CDCl_3_) δ 0.83 (t, 3H, *J* = 10 Hz); δ 1.37 (m, 3H); δ 1.48 (dt, 1H, *J* = 25 Hz, 10 Hz); δ 1.93 (t, 1H, *J* = 10 Hz); δ 2.05 (d, 1H, *J* = 15 Hz); δ 3.12 (m, 1H); 3.43 (m, 1H); δ 3.89 (m, 1H); δ 4.04 (m, 2H); δ 5.41 (d, 1H, *J* = 10 Hz); δ 5.74 (d, 1H, *J* = 10 Hz); δ 6.30 (d, 1H); δ 7.17 (dd, 1H, *J* = 7.5 Hz); δ 7.38 (d, 1H, *J* = 7.5 Hz). RMN ^13^C (125 MHz; CDCl_3_) δ 9.78; 28.63; 38.52; 41.66; 55.08; 66.66; 72.77; 72.87; 74.03; 78.23; 121.81; 127.07; 127.11; 128.32; 131.57; 140.29; 140.40; 141.41; 141.52; 165.68. LC-MS-IT-TOF calculated for C_18_H_23_BrO_4_Na: 405.0672 [M + H]^+^; found: 405.06756.

### 3.5. Biology

#### 3.5.1. *Leishmania* Culture

*Leishmania (Leishmania) donovani* (MHOM/ET/1967/HU3) was provided by Oswaldo Cruz Institute *Leishmania* Collection-Fiocruz-RJ-Brazil. Promastigotes were maintained in vitro at 26 °C in Schneider’s medium, pH 7 (20% heat-inactivated fetal bovine serum (FBS), 100 U/mL penicillin, 100 mg/mL streptomycin, and 2% of male urine), as previously described [29].

#### 3.5.2. Effect of Morita-Baylis-Hillman Adduct in Promastigotes of *Leishmania donovani*

Briefly, promastigotes in the logarithmic growth phase were cultured in 96-well cell culture plates at 1 × 10^6^ parasites per well in 100 mL of Schneider's medium with increasing concentrations of 3.12, 6.25, 12.5, 25, 50, 100, 200, and 400 µg/mL of Morita-Baylis-Hillman adduct. The plates were incubated for 72 h in a biological oxygen demand (B.O.D.) incubator at 26 °C. Cytotoxicity to promastigotes was evaluated by the 3-(4,5-dimethyl-2-thiazole)-2,5-diphenyltetrazolium bromide (MTT) colorimetric assay as described by Valadares et al. [30].The absorbance was measured using an ELISA plate reader (ELx800, BIOTEK) at 540 nm. Amphotericin^®^ was used as a positive control. The viability of promastigotes incubated in the presence of different concentrations of adducts was determined by comparing to a culture control (*L. donovani* cultivated in Schneider only). The concentration that caused a 50% reduction in cell viability (IC_50_) was calculated by Probit analysis (SPSS 13.0 for Windows). Each experiment was performed in duplicate and repeated at least three times. Haemolytic assays (HC_50_ > 400 μg·L^–1^) towards human red blood cells were performed as described in the literature [30].

## 4. Conclusions 

The synthesis of the sixteen new hybrids was done in two steps in moderate to very good yields, through the reaction of MBHAs with tetrahydropyran acrylates and aromatic aldehydes. Studies of the variation of reaction conditions, such as the use of microwaves promoting the reaction at 80 °C, use of low temperature, protic or aprotic solvents, and the use of phenol as additives were used satisfactorily based on the knowledge of MBHR reaction mechanisms. The success of the biological results of leishmanicidal activities and high indices of selectivities of several hybrids, especially those which present a bromine atom in the aromatic structure (SIrb > 180.19) higher than the reference drug Amphotericin B (SI_rb_ = 33.05), which presents high toxicity, indicate that our proposal that hybridization of opioid analgesic tetrahydropyrans with MBHA was a good strategy. This also shows a good possibility to obtain new leishmanicidal drugs against *Leishmania donovani* ssp.

## Figures and Tables

**Table 1:**
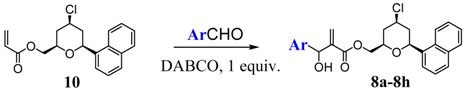
Conditions and yields in the synthesis of new hybrids **8a**–**8h**.

Entry	Ar	Hybrid	Solvent ^a^	Temp. (°C) ^b^	Time (h)	Yield (%) ^c^
1	*o*-nitrophenyl	**8a**	t-buOH/H_2_O	80 Mw	1	85
2	*m*-nitrophenyl	**8b**	t-buOH/H_2_O	80 Mw	0.7	34^d^
3	*m*-nitrophenyl	**8b**	DMF:H_2_O	0	48	81
4	*p*-nitrophenyl	**8c**	t-buOH/H_2_O	80 Mw	0.7	86
5	2-pyridinyl	**8d**	t-buOH/H_2_O	80 Mw	1.3	81
6	3-pyridinyl	**8e**	t-buOH/H_2_O	80 Mw	2	80
7	4-pyridinyl	**8f**	t-buOH/H_2_O	80 Mw	1	87
8	α-naphthyl	**8g**	t-buOH/H_2_O	80 Mw	2	n.r.
9	α-naphthyl	**8g**	DMF:H_2_O	0	48	45
10	*p*-bromophenyl	**8h**	t-buOH/H_2_O	80 Mw	2	n.r.
11	*p*-bromophenyl	**8h**	DMF:H_2_O	0	240	60

^a^ Mixture of 9:1; ^b^ Mw = microwave irradiation; ^c^ isolated yields; ^d^ co-products were observed; n.r.= no reaction.

**Table 2:**
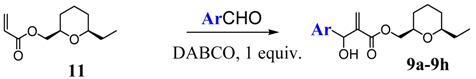
Conditions and yields in the synthesis of new hybrids **9a**–**9h.**

Entry	Ar	Hybrid	Solvent ^a^	Temp. (°C) ^b^	Time (h)	Yield (%) ^c,d^
1	*o*-nitrophenyl	**9a**	t-buOH/H_2_O	80 Mw	0.7	74
2	*m*-nitrophenyl	**9b**	t-buOH/H_2_O	80 Mw	0.7	78
3	*p*-nitrophenyl	**9c**	t-buOH/H_2_O	80 Mw	0.3	95
4	2-pyridinyl	**9d**	t-buOH/H_2_O	80 Mw	0.8	96
5	3-pyridinyl	**9e**	t-buOH/H_2_O	80 Mw	1.3	80
6	4-pyridinyl	**9f**	t-buOH/H_2_O	80 Mw	0.7	72
7	4-pyridinyl	**9f**	t-buOH/H_2_O, PhOH	80 Mw	0.3	96
8	α-naphthyl	**9g**	t-buOH/H_2_O	80 Mw	2	n.r.
9	α-naphthyl	**9g**	DMF/H_2_O, PhOH	0	96	65
10	*p*-bromophenyl	**9h**	t-buOH/H_2_O	80 Mw	2	n.r.
11	*p*-bromophenyl	**9h**	DMF/H_2_O, PhOH	0	240	50

^a^ Mixture of 9:1; ^b^ Mw = microwave irradiation; ^c^ isolated yields; ^d^ co-products were not observed.

**Table 3:** In vitro biological results.

Compounds	IC_50_^a^ (μg·mL^−1^)	IC_50_^a^ (μM)	HC_50_^b^ (μg·mL^−1^)	HC_50_^b^ (μM)	SI_rb_^c^
**6**	47.53 ± 39	172.19 ± 141.25	-	-	-
**7**	>400	>2775.46	-	-	-
**8a**	7.09 ± 3.0	14.74 ± 6.23	>400	>831.38	>56.40
**8b**	8.76 ± 4.0	18.21 ±8.31	-	-	-
**8c**	9.05 ± 3.3	18.81 ± 6.85	-	-	-
**8d**	61.93 ± 18	141.67 ± 41.17	-	-	-
**8e**	8.76 ± 3.8	20.04 ± 8.69	-	-	-
**8f**	8.96 ± 4.5	20.50 ± 10.29	-	-	-
**8g**	24.51± 7.5	50.42 ± 15.42	-	-	-
**8h**	17.17 ± 5.6	33.40 ± 10.90	-	-	-
**9a**	5.64 ± 3.0	16.15 ± 8.60	>400	>1145.64	>70.94
**9b**	13.75 ± 9.8	39.38 ± 28.06	-	-	-
**9c**	7.03 ± 4.3	20.13 ± 12.31	>400	>1145.64	>56.91
**9d**	72.32 ± 26.0	240.27 ± 85.20	-	-	-
**9e**	17.68 ± 7.7	57.94 ± 25.23	-	-	-
**9f**	57.76 ± 20.0	189.28 ± 65.53	-	-	-
**9g**	9.73 ± 4.8	27.47 ± 13.55	-	-	-
**9h**	2.22 ± 7.6	5.81 ± 19.90	>400	>1046.90	>180.19
*Amphotericin B*	0.35 ± 0.27	0.38 ± 0.30	11.61	12.56	33.05

^a^ The half maximal inhibitory concentration; ^b^ 50% hemolysis in red blood cells; ^c^ SI_rb_ (selectivity index on red blood cell) = HC_50_/IC_50_.

## Supplementary Materials

The following are available online at www.mdpi.com/1420-3049/22/2/207/s1.
