# Supplementary material for: Synthesis of 16 New Hybrids from Tetrahydropyrans Derivatives and Morita-Baylis-Hillman Adducts: In Vitro Screening against Leishmania donovani
Source: Molecules. 2017 Jan 30;22(2):207. doi: 10.3390/molecules22020207 (PMC6155752; doi:10.3390/molecules22020207)
Supplement: Supplementary file 1 [file molecules-22-00207-s001.pdf]

Supplementary Materials: Synthesis of 16 New Hybrids from Tetrahydropyrans Derivatives and Morita- Baylis- Hillman Adducts: In Vitro Screening against Leishmania donovani

Suervy Canuto de Oliveira Sousa, Juliana da Câmara Rocha, Tatjana de Souza Lima Keesen, Everton da Paz Silva, Priscilla Anne Castro de Assis, João Paulo Gomes de Oliveira, Saulo Luís Capim, Francisco José Seixas Xavier, Bruno Guimarães Marinho, Fábio Pedrosa Lins Silva, Claudio Gabriel Lima Junior, and Mário Luiz Araújo de Almeida Vasconcellos

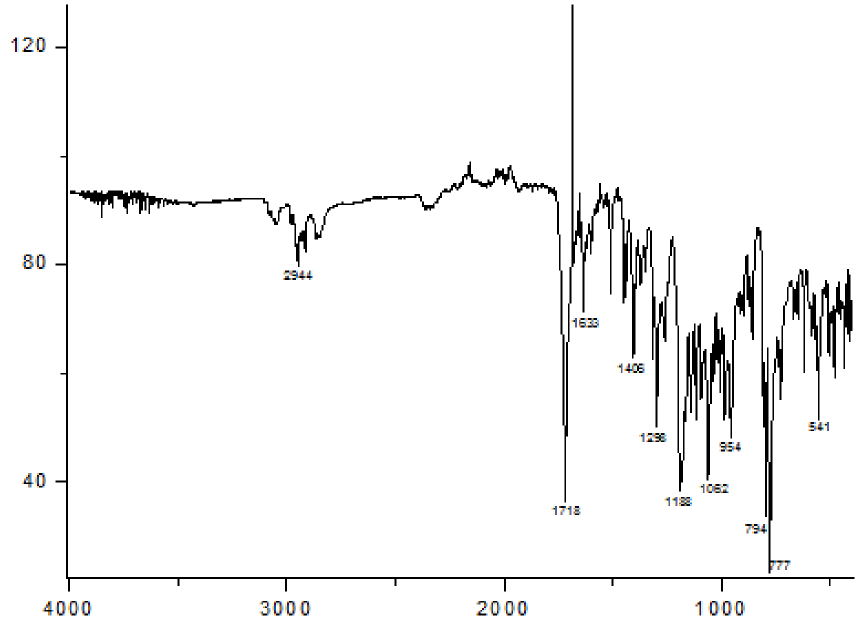

Figure S1. FTIR spectrum of acrylate **10**.

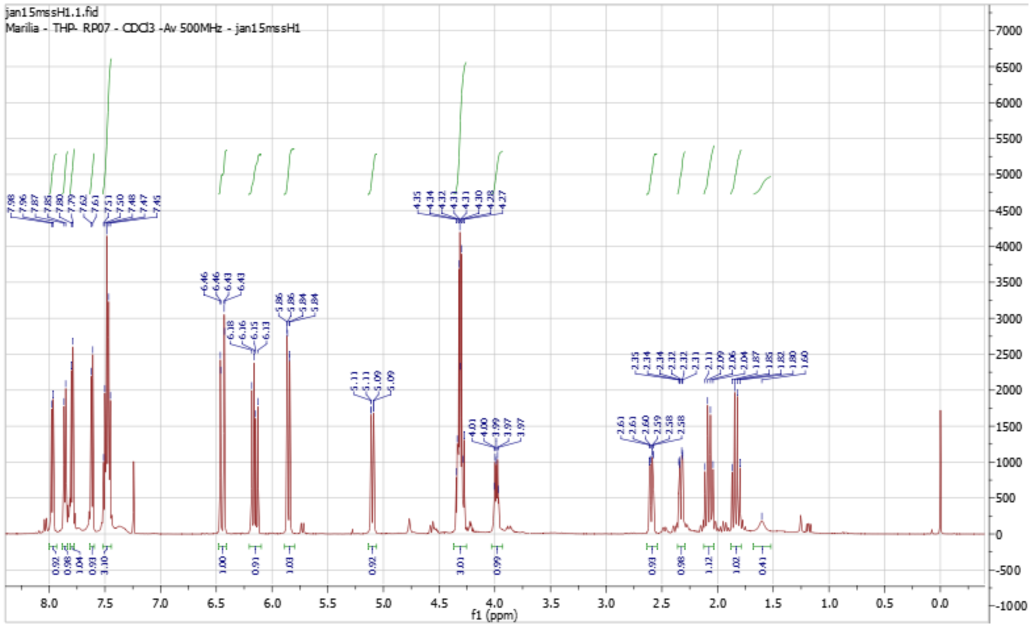

Figure S2. RMN ^{1}H spectrum of acrylate **10**.

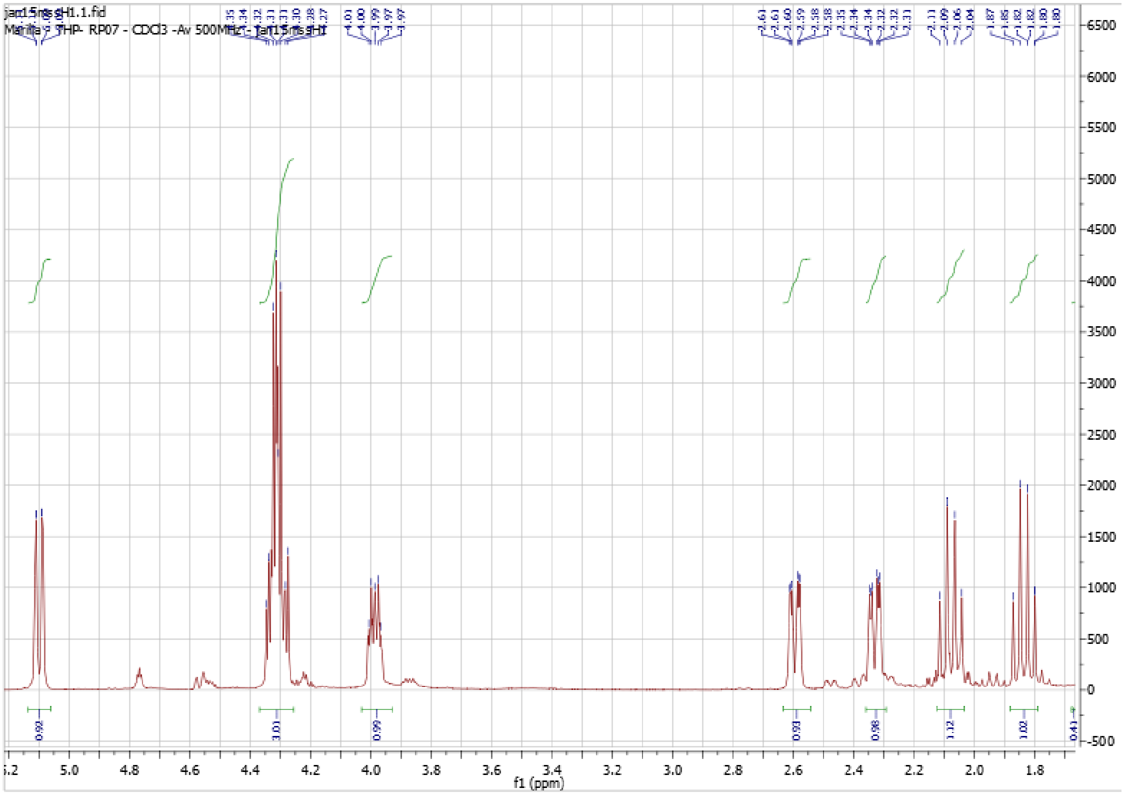

Figure S3. RMN ^{1}H spectrum enlargement of acrylate **10** (1.8 – 5.2ppm)

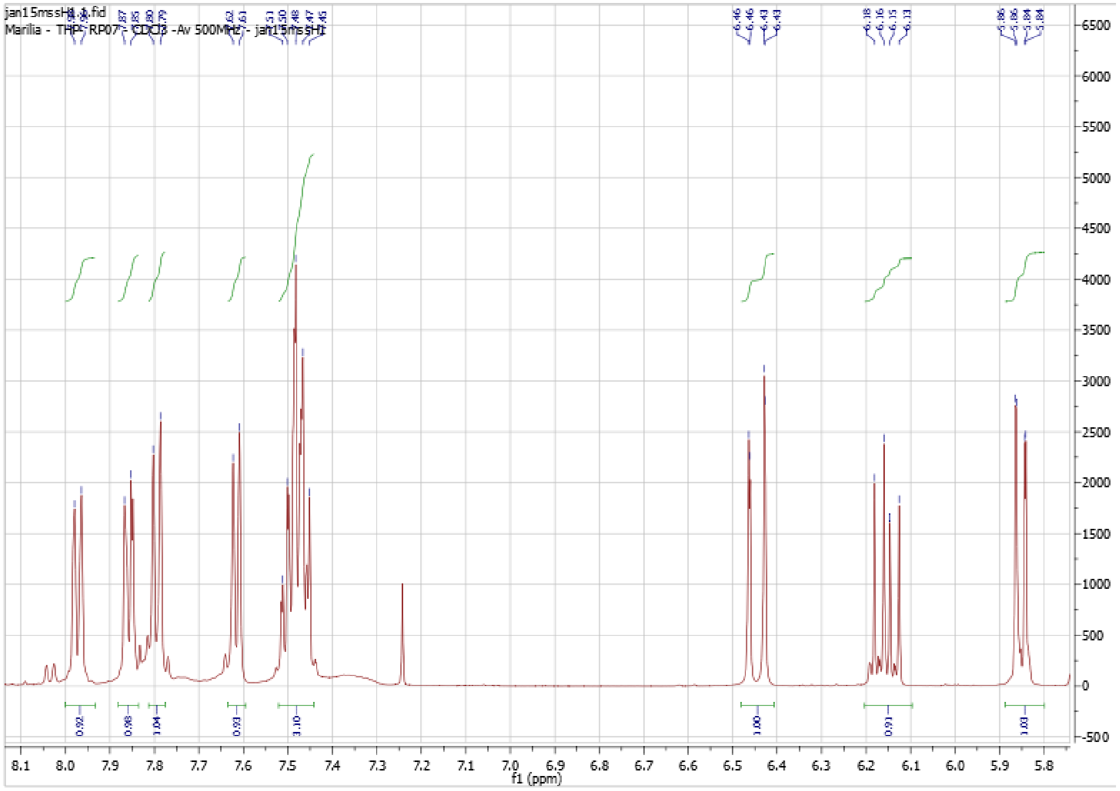

Figure S4. RMN ^{1}H spectrum enlargement of acrylate **10** (5.8 – 8.0ppm)

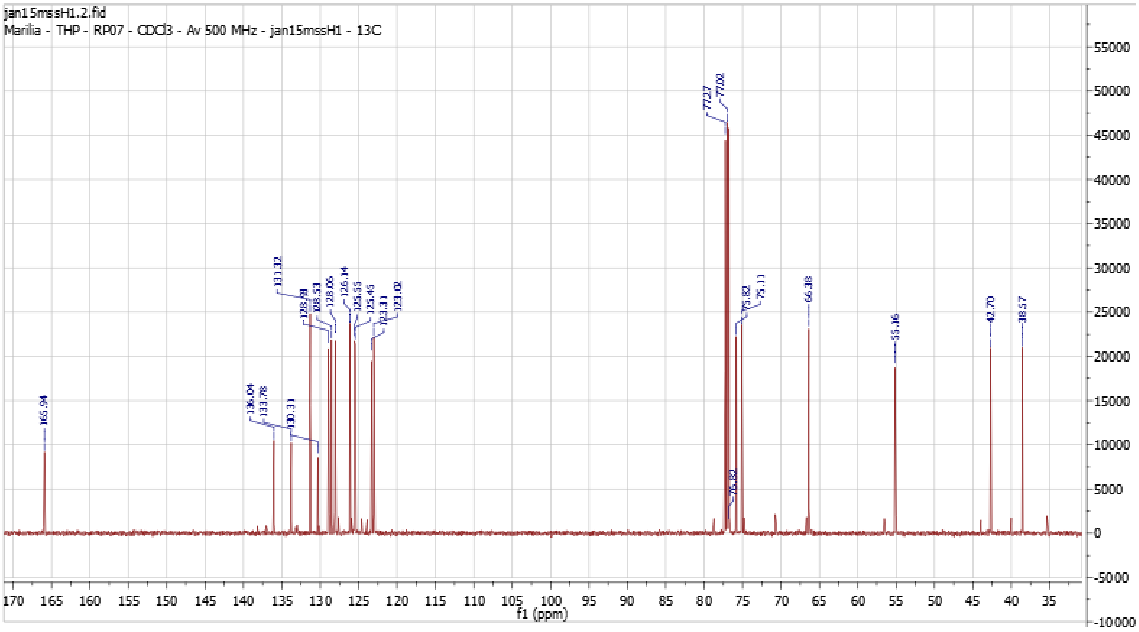

Figure S5. RMN ^{13}C spectrum of acrylate **10**.

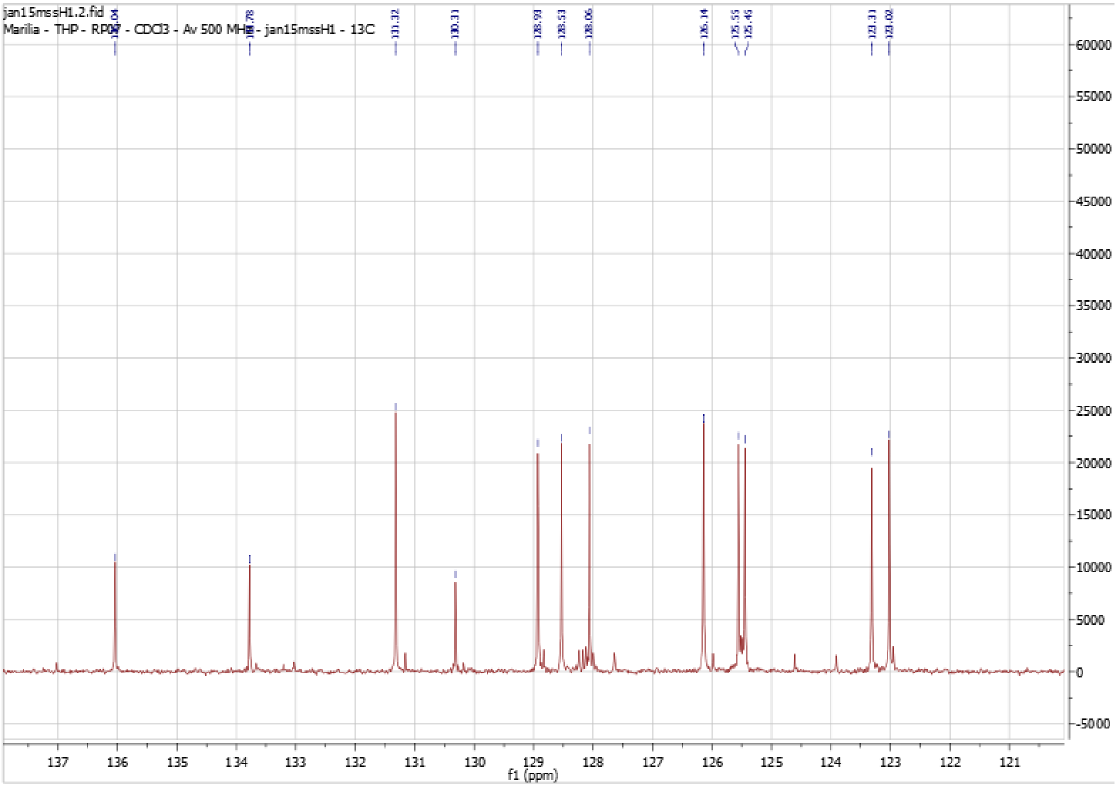

Figure S6. RMN ^{13}C spectrum enlargement of acrilate **10** (122 – 137ppm).

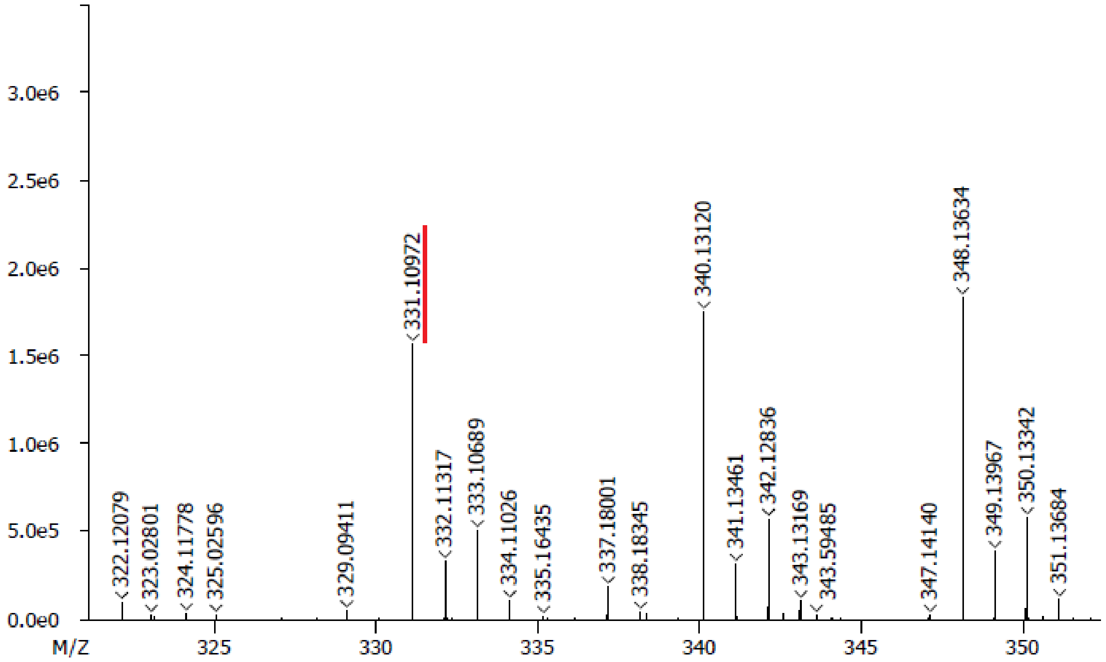

Figure S7. HRMS spectrum of acrylate **10**.

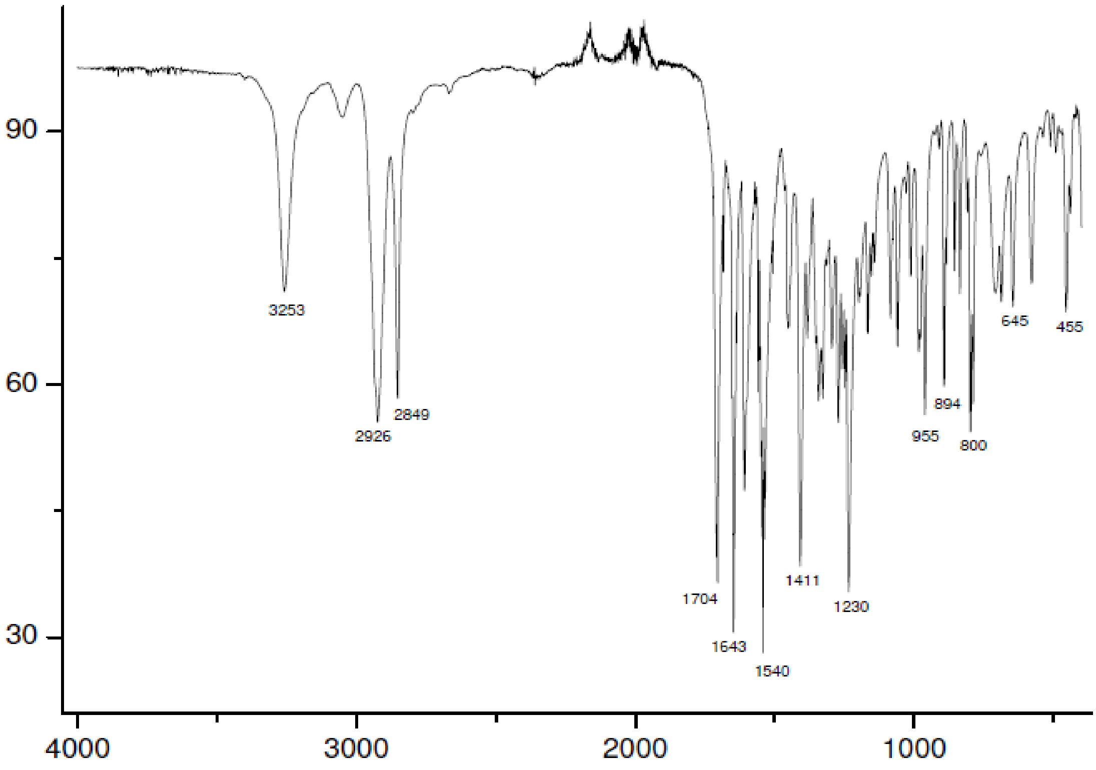

Figure S8. FTIR spectrum of acrylate **11**.

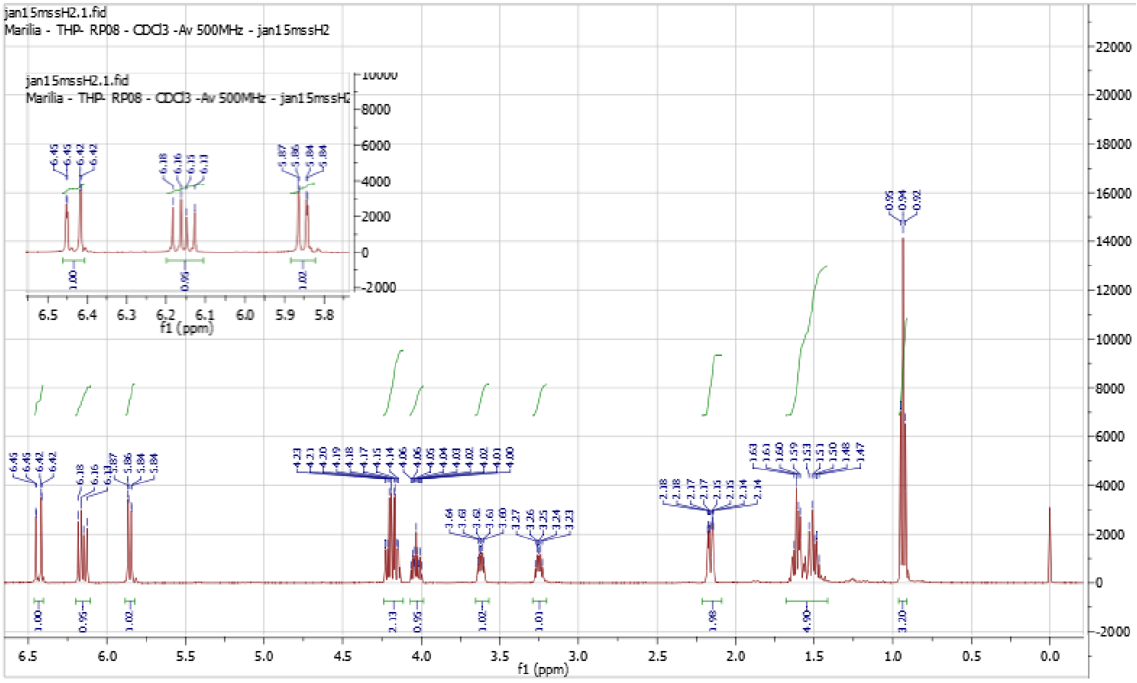

Figure S9. RMN ^{1}H spectrum of acrilate **11**.

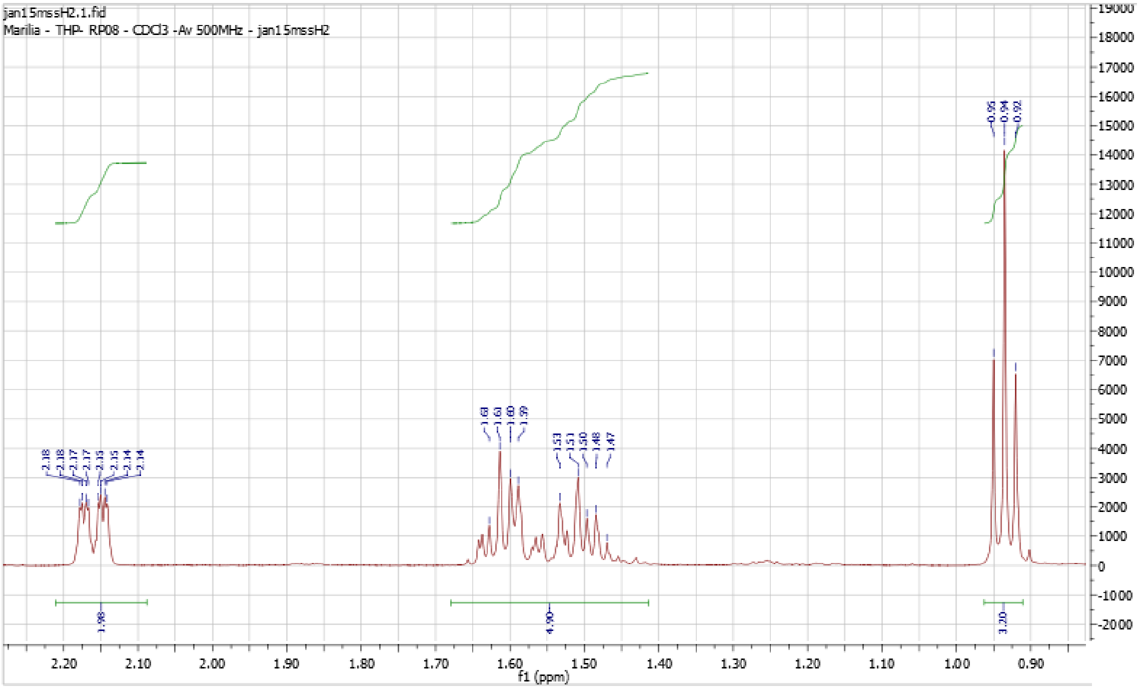

Figure S10. RMN ^{1}H spectrum enlargement of acrilate **11** (0.9 – 2.2ppm).

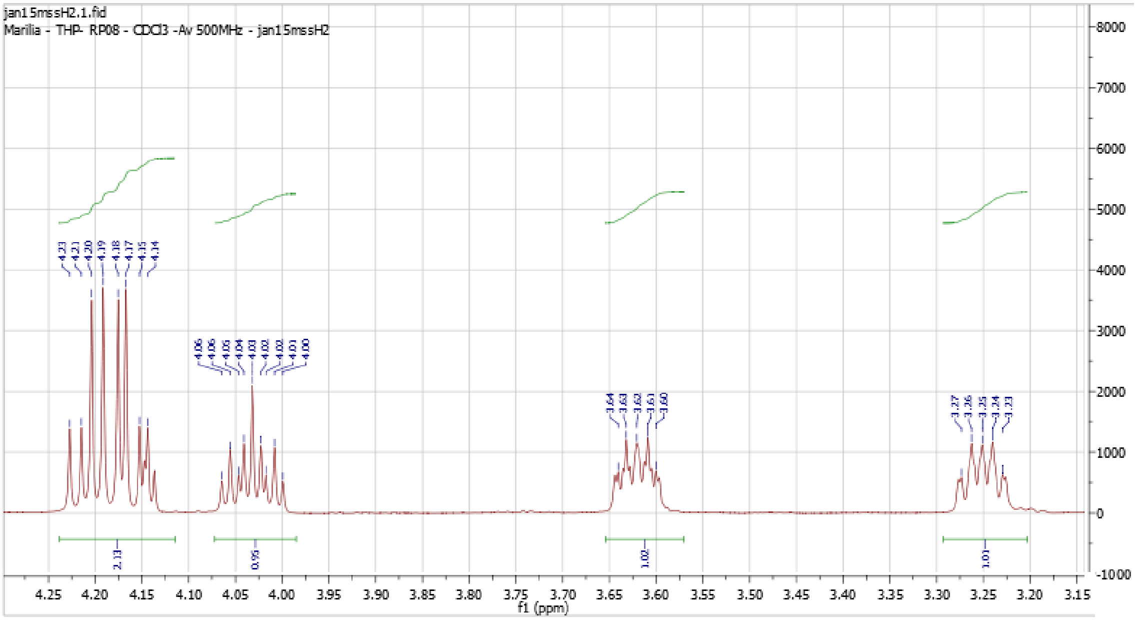

Figure S11. RMN ^{1}H spectrum enlargement of acrilate **11** (3.2 – 4.25ppm).

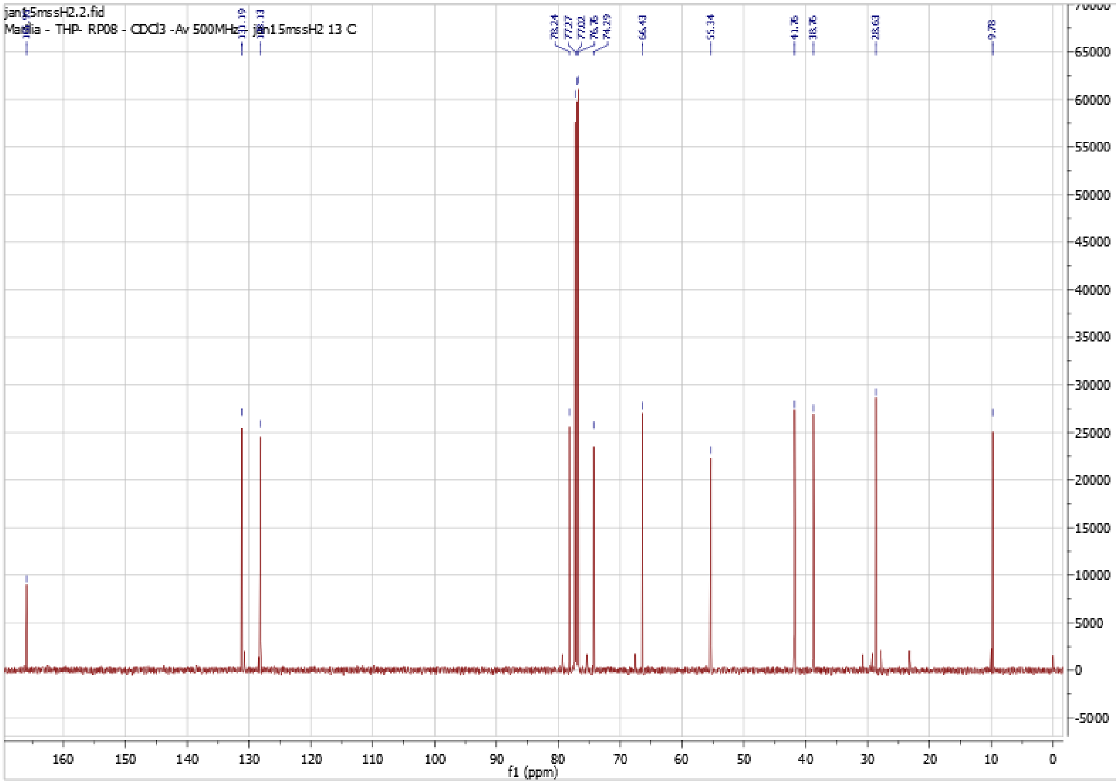

Figure S12. RMN ^{13}C spectrum of acrylate **11**.

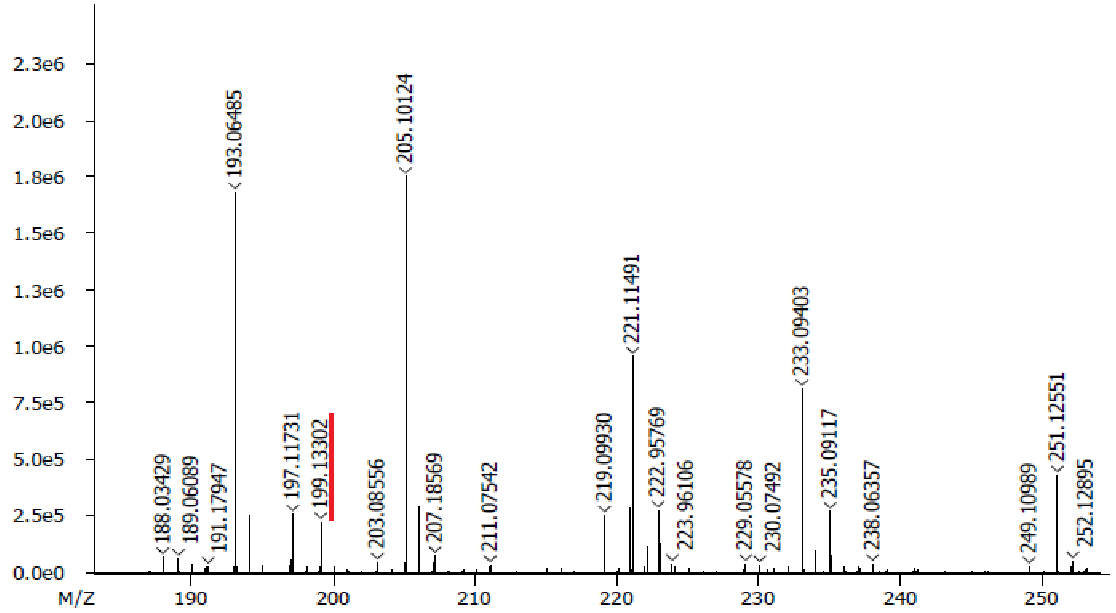

Figure S13. HRMS spectrum of acrylate **11**.

4. Spectroscopic data of adducts (8a – 8h) and (9a – 9h)

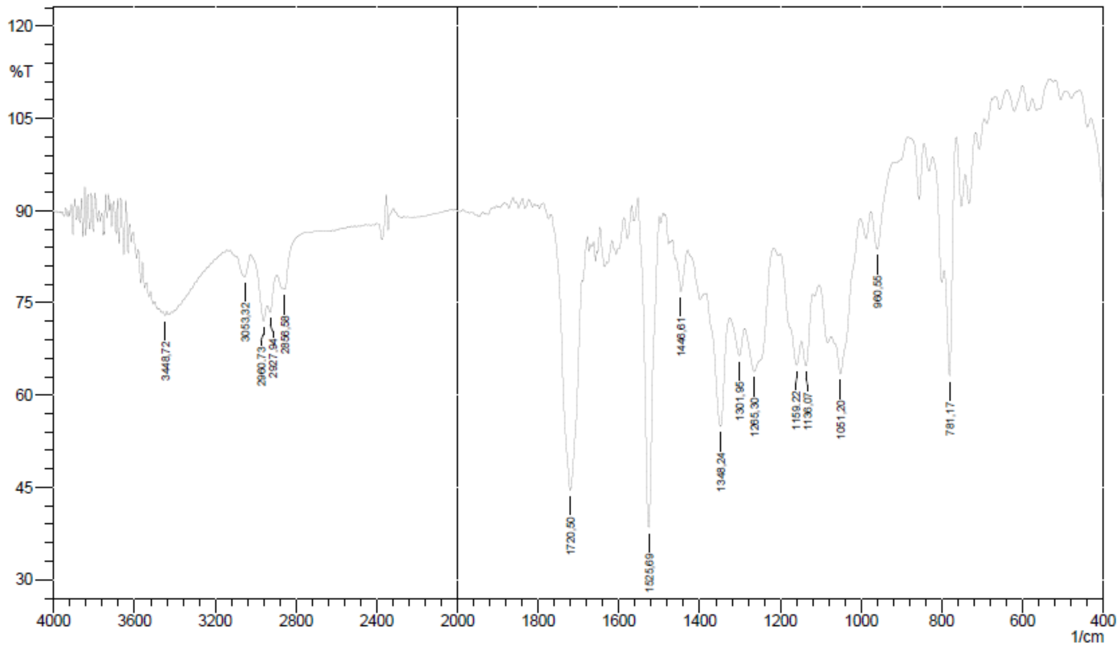

Figure S14. FTIR spectrum of adduct **8a**.

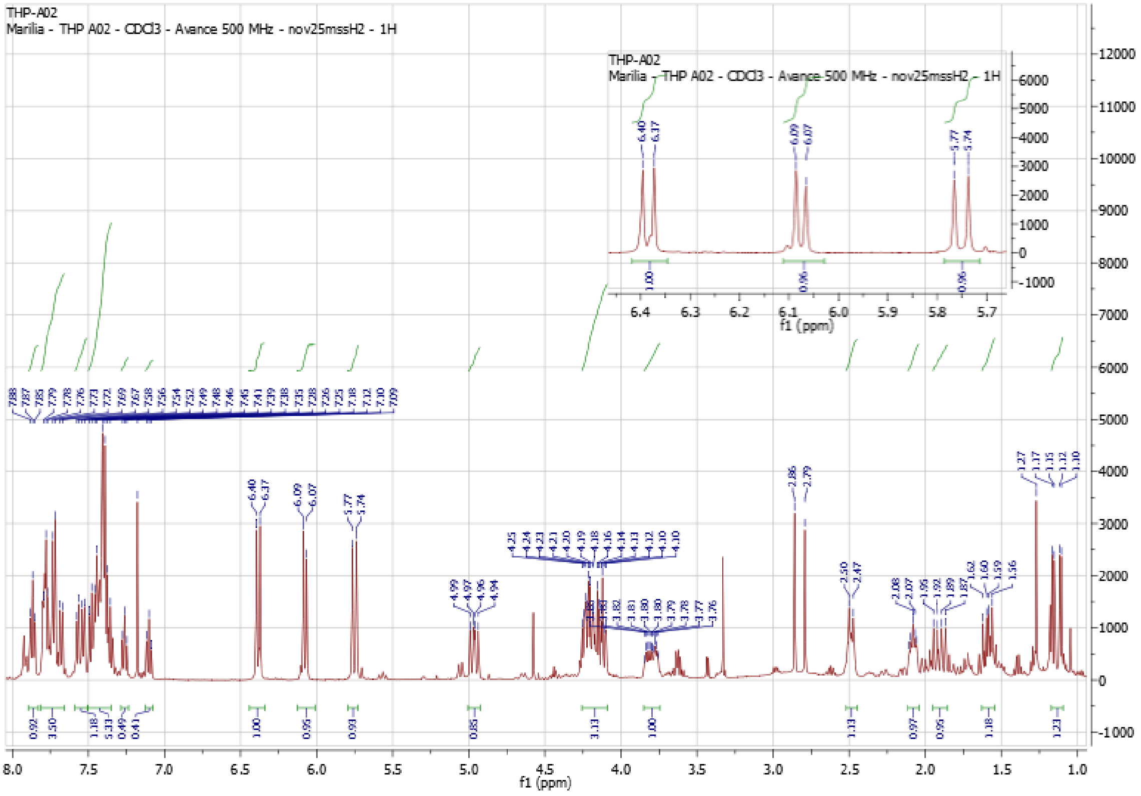

Figure S15. RMN ^{1}H spectrum of adduct **8a**.

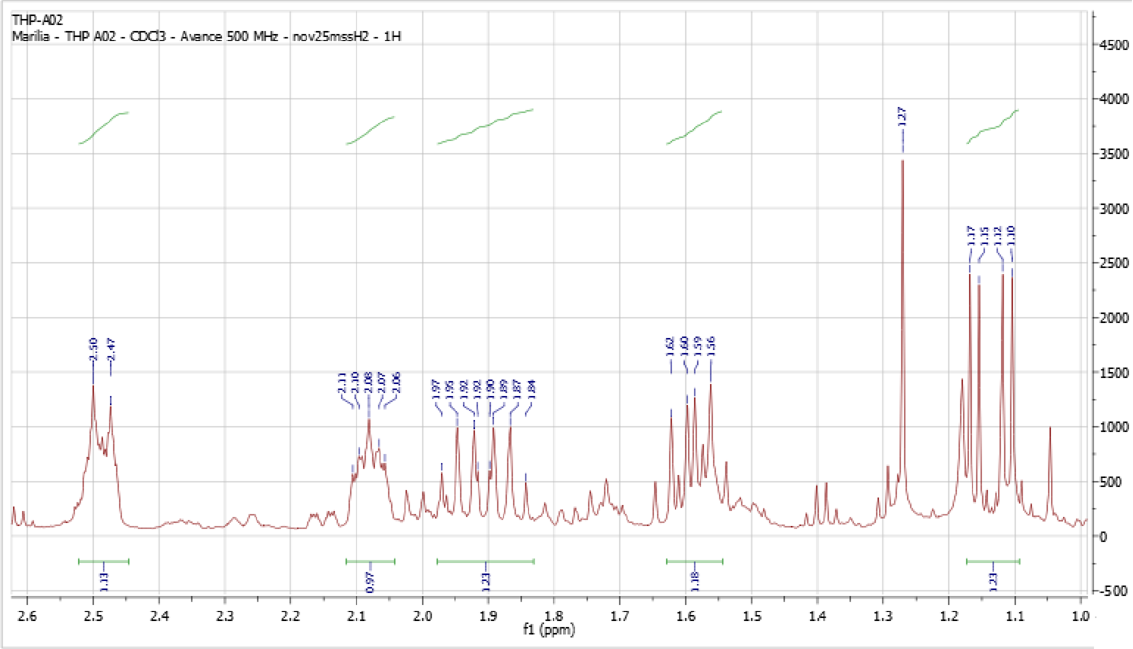

Figure S16. RMN ^{1}H spectrum enlargement of adduct **8a** (1.0 – 2.55ppm).

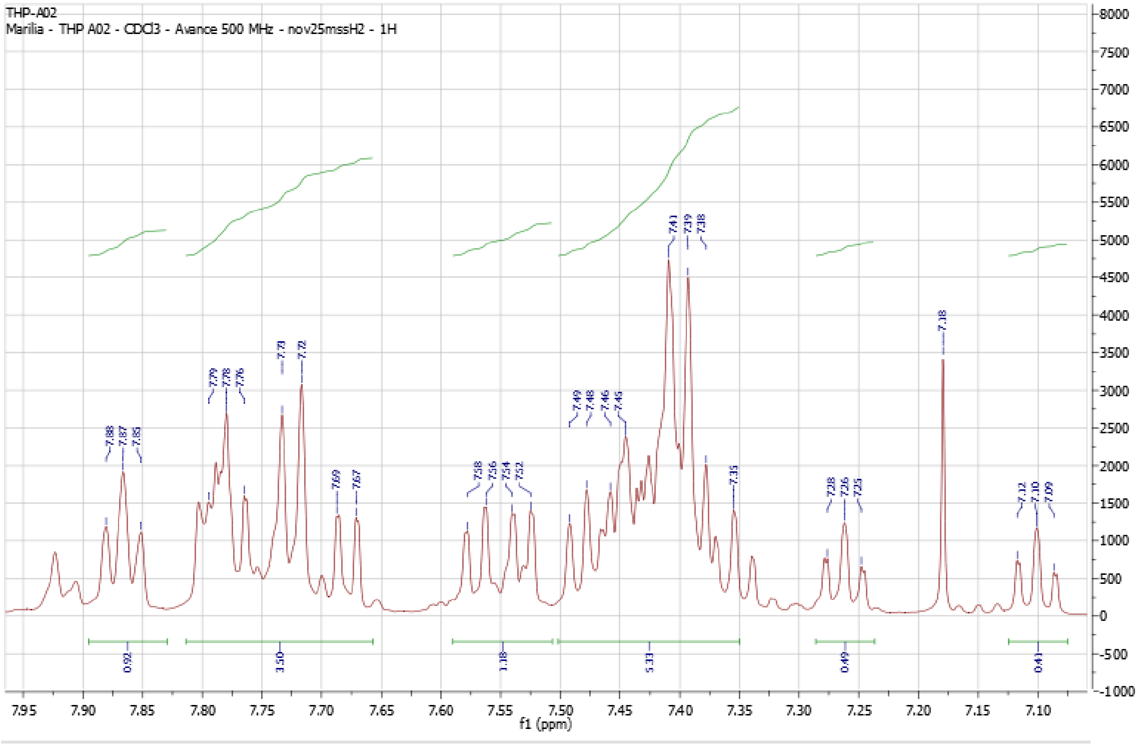

Figure S17. RMN ^{1}H spectrum enlargement of adduct **8a** (7.95 – 7.9ppm).

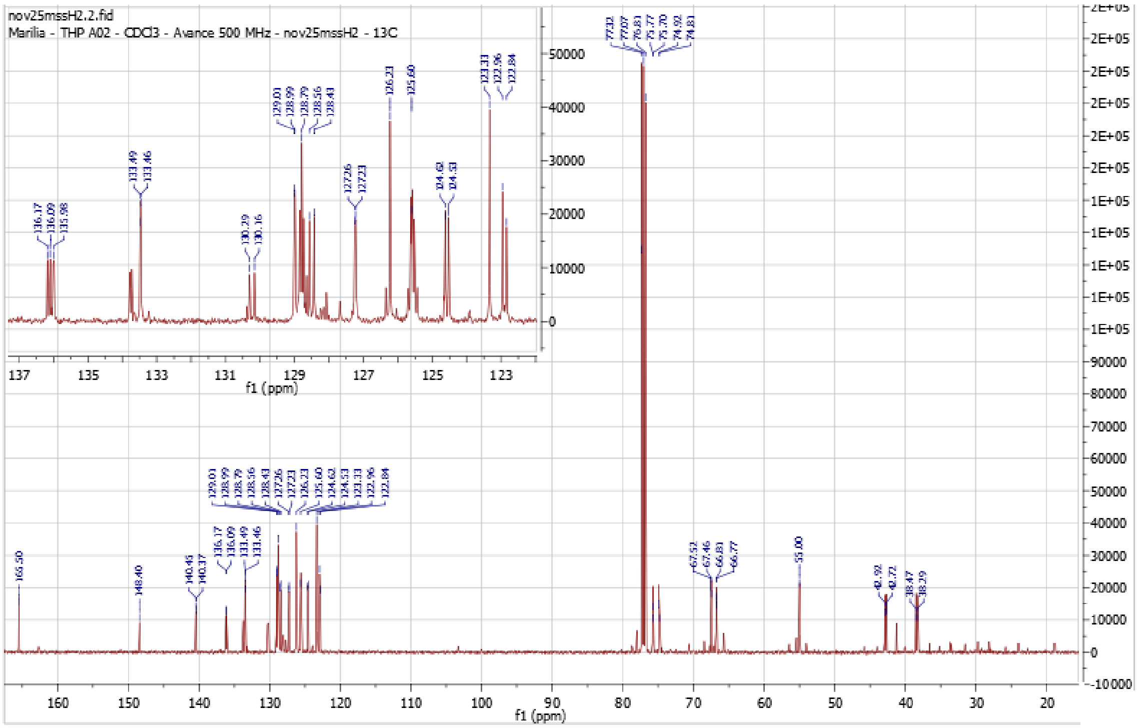

Figure S18. RMN ^{13}C spectrum of adduct **8a**.

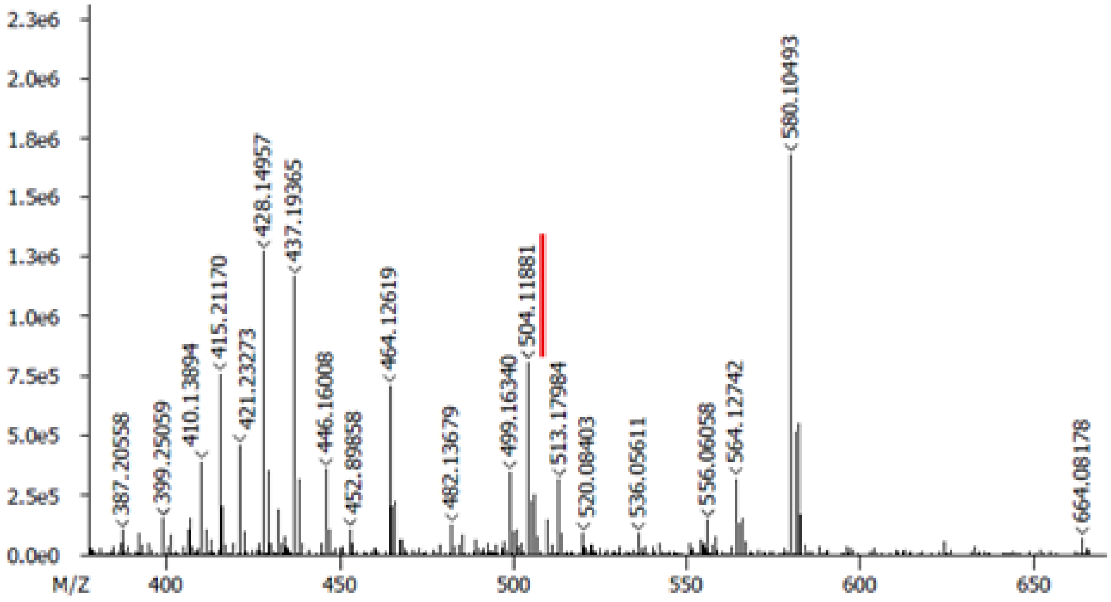

Figure S19. HRMS spectrum of compound **8a**.

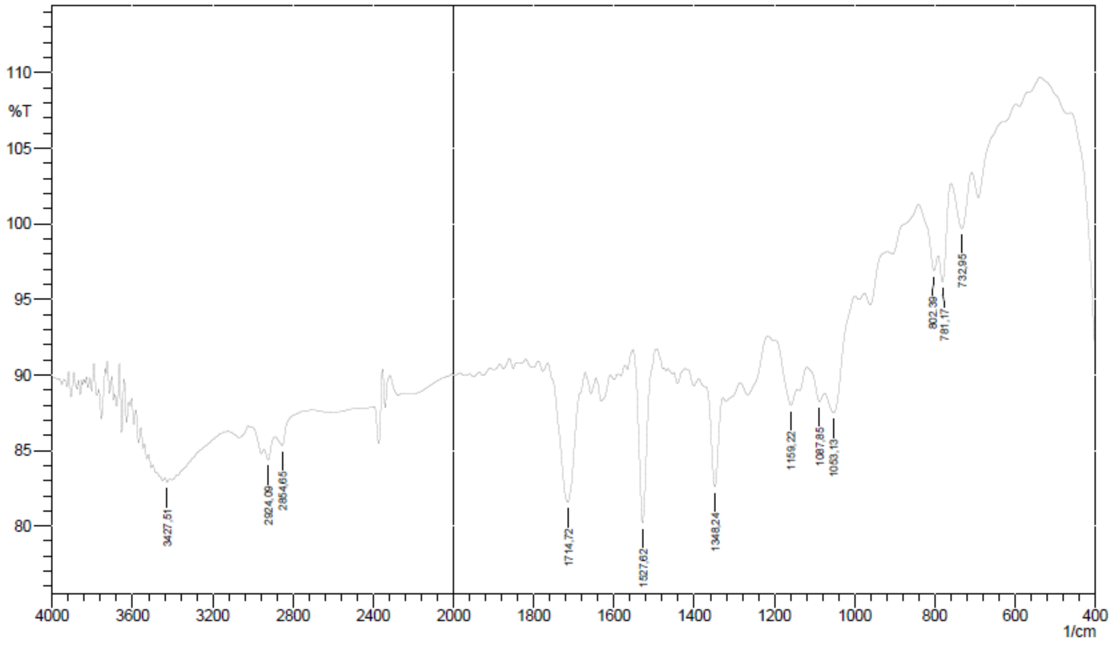

Figure S20. FTIR spectrum of adduct **8b**.

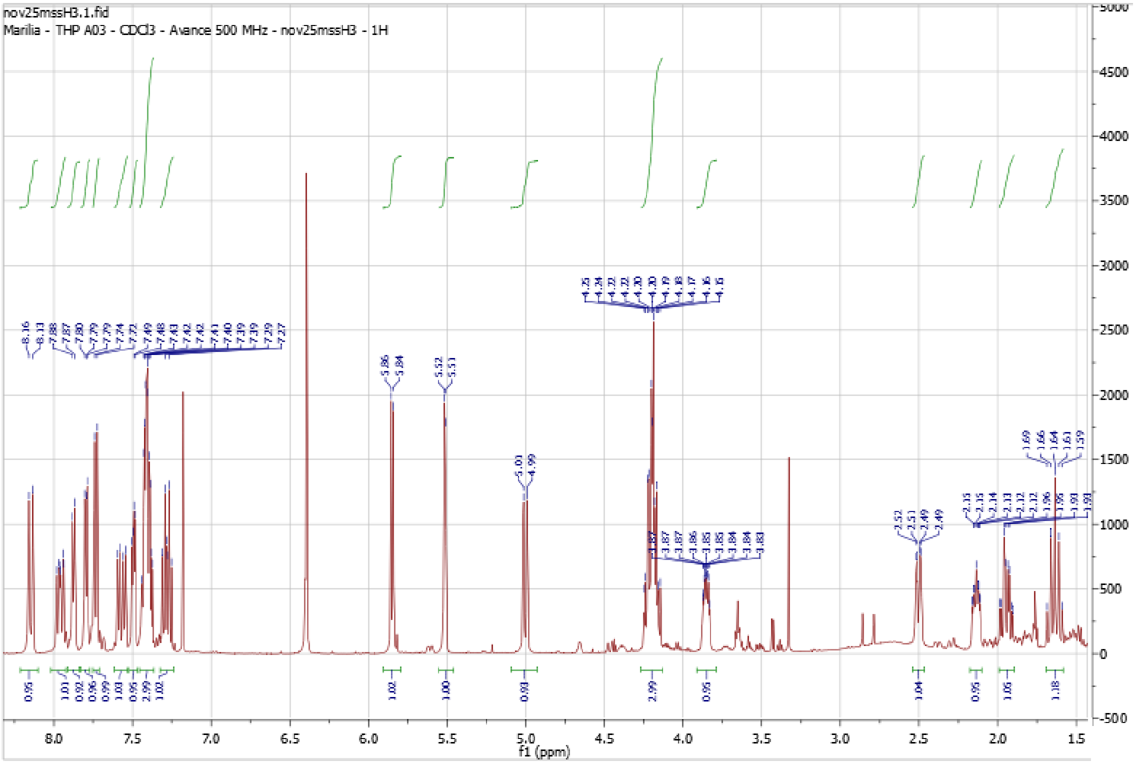

Figure S21. RMN ^{1}H spectrum of adduct **8b**.

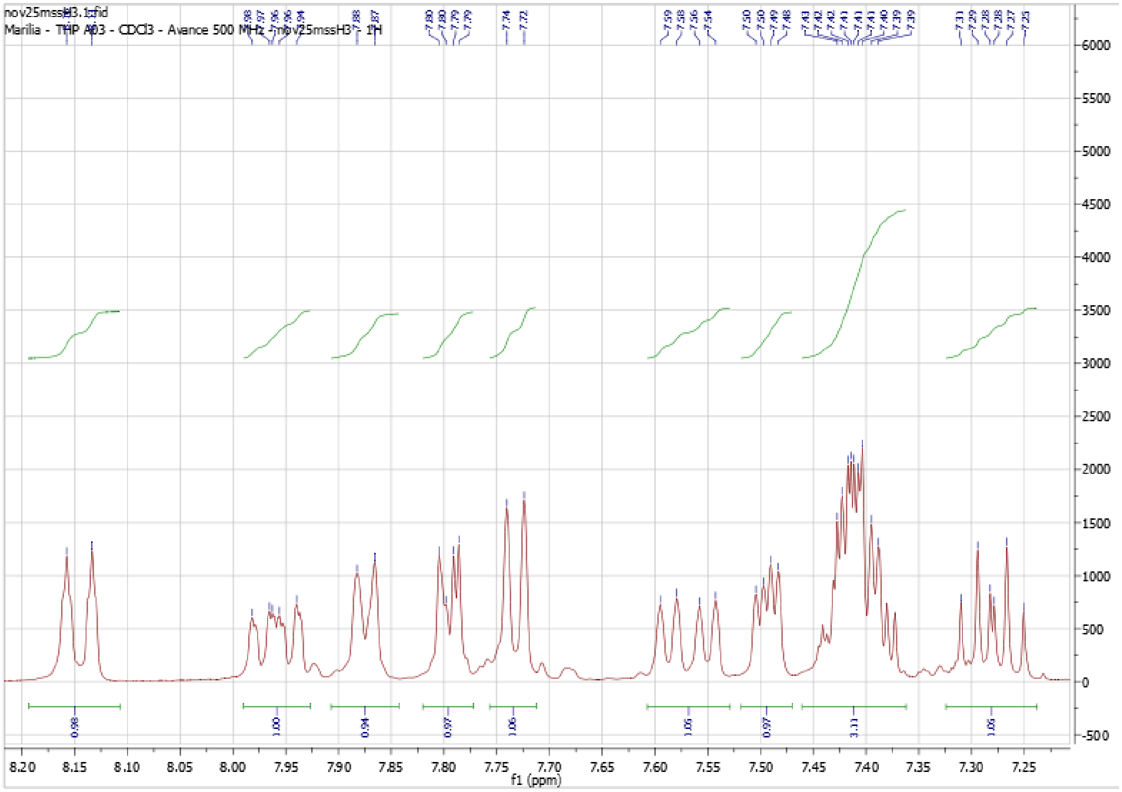

Figure S22. RMN ^{1}H spectrum enlargement of adduct **8b** (7.2 – 8.2ppm).

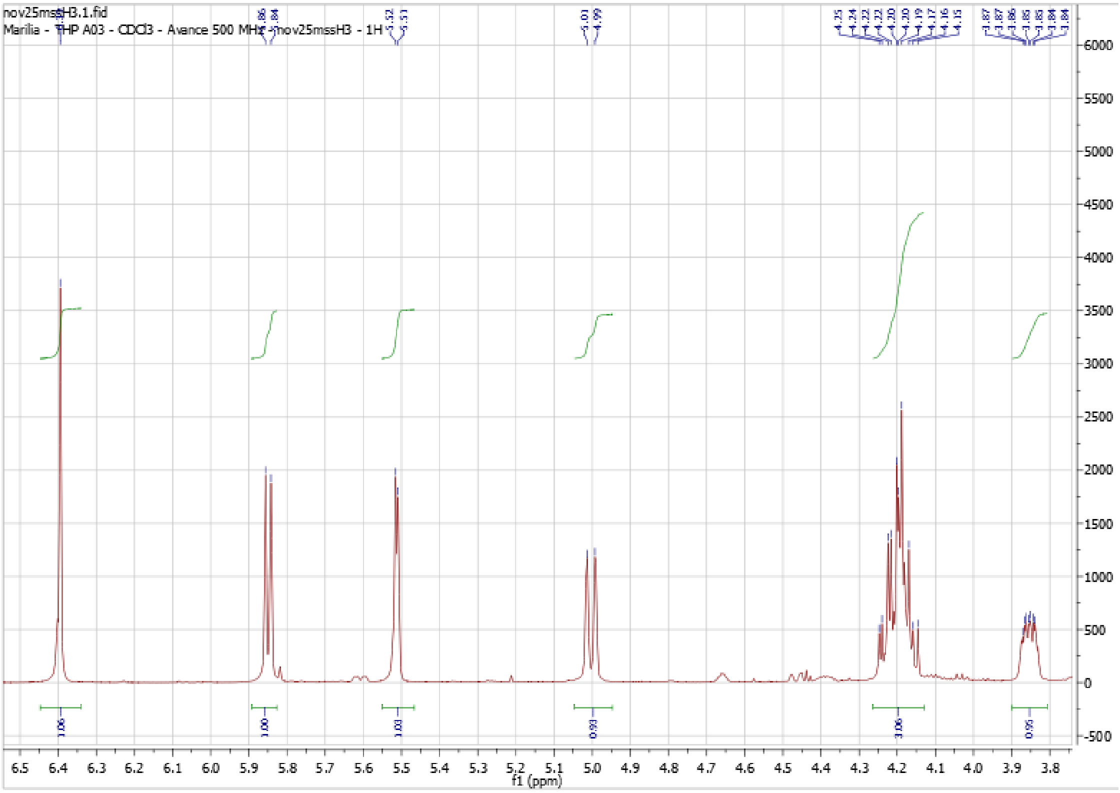

Figure S23. RMN ^{1}H spectrum enlargement of adduct **8b** (3.8 – 6.5ppm).

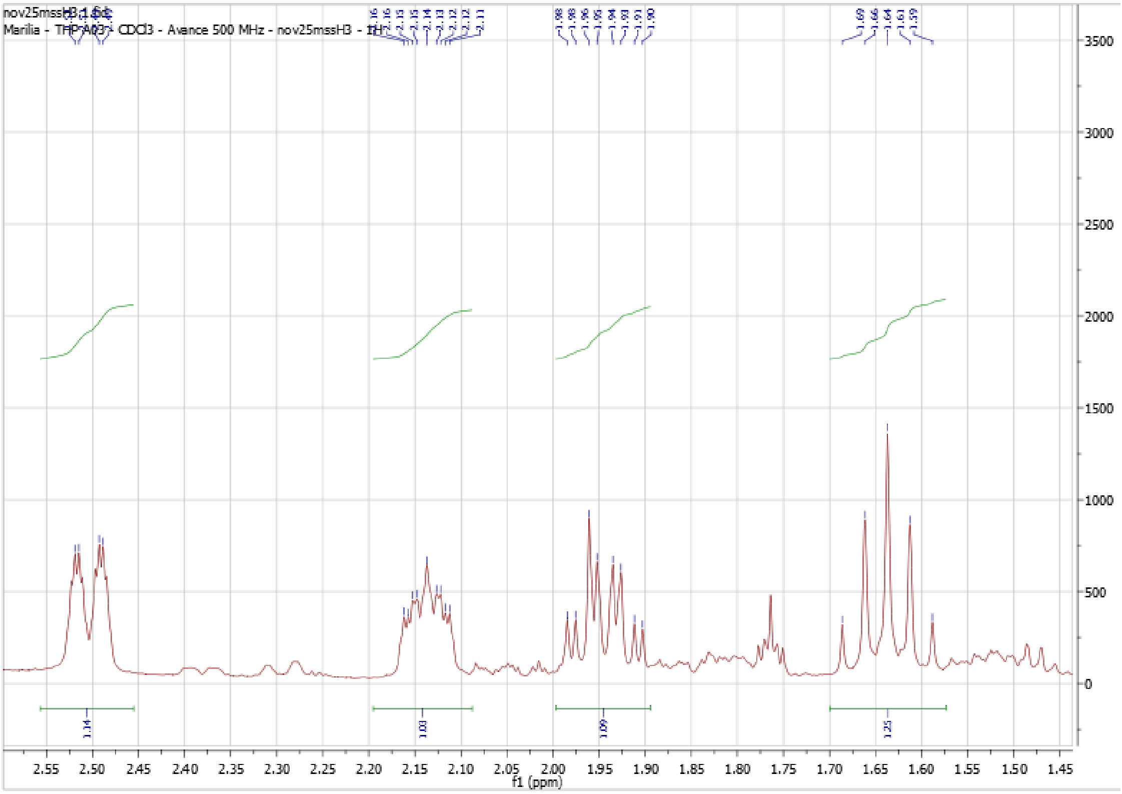

Figure S24. RMN ^{1}H spectrum enlargement of adduct **8b** (1.5 – 2.6ppm).

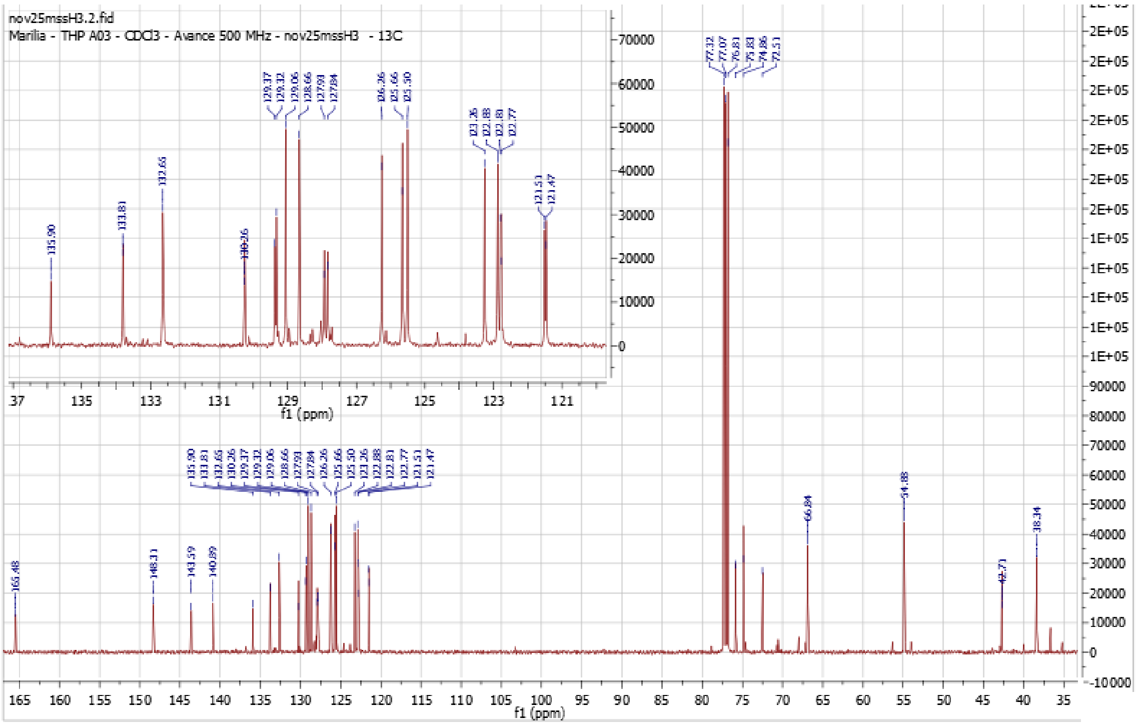

Figure S25. RMN ^{13}C spectrum of adduct **8b**.

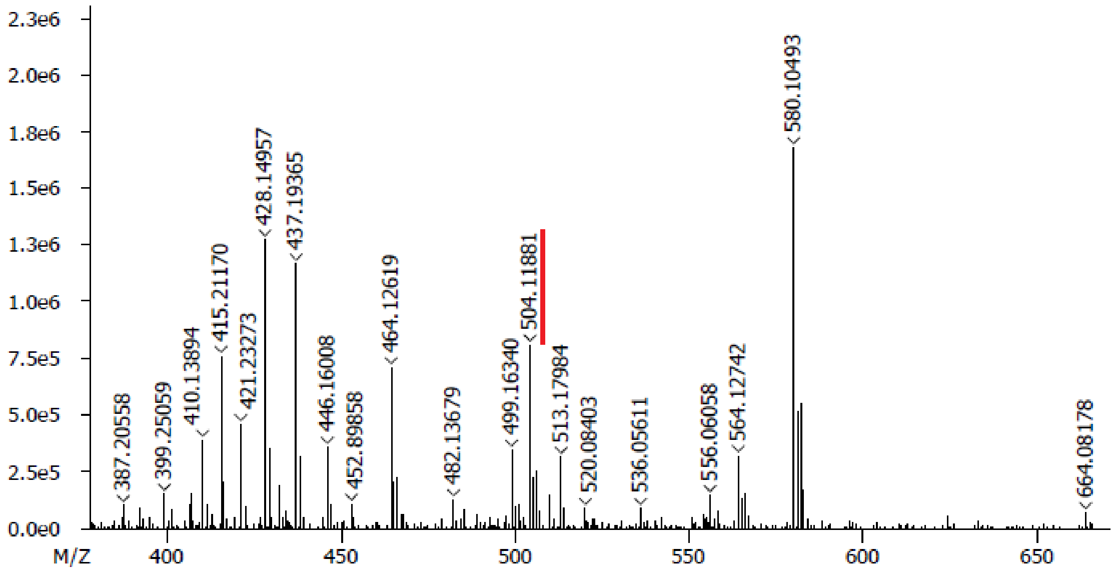

Figure S26. HRMS spectrum of compound **8b**.

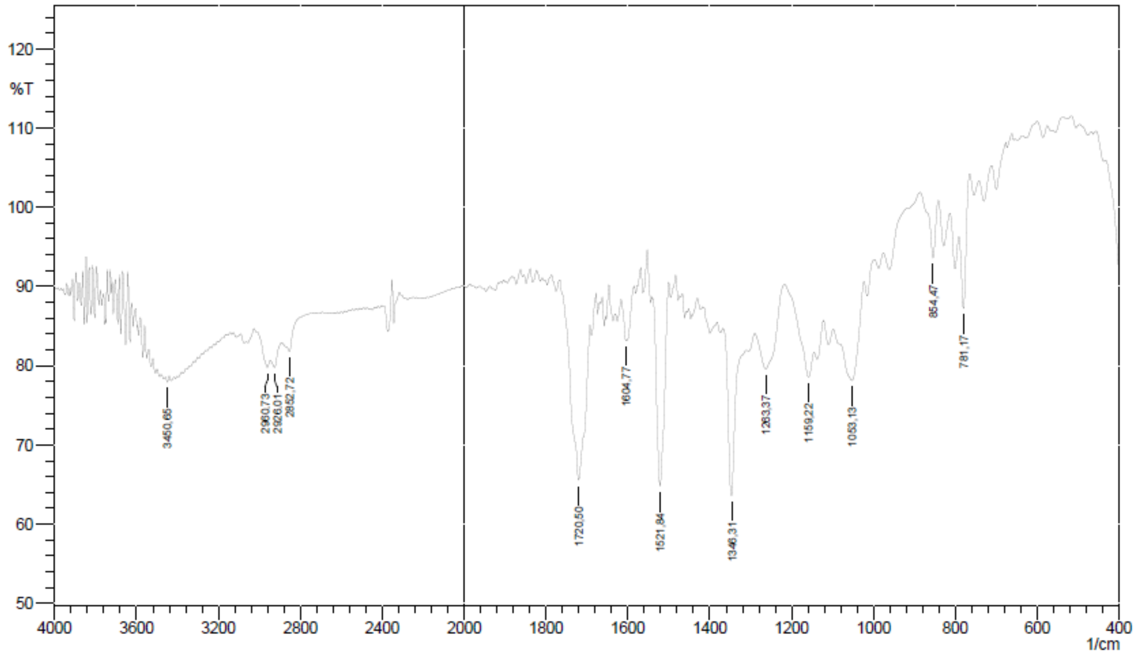

Figure S27. FTIR spectrum of adduct **8c**.

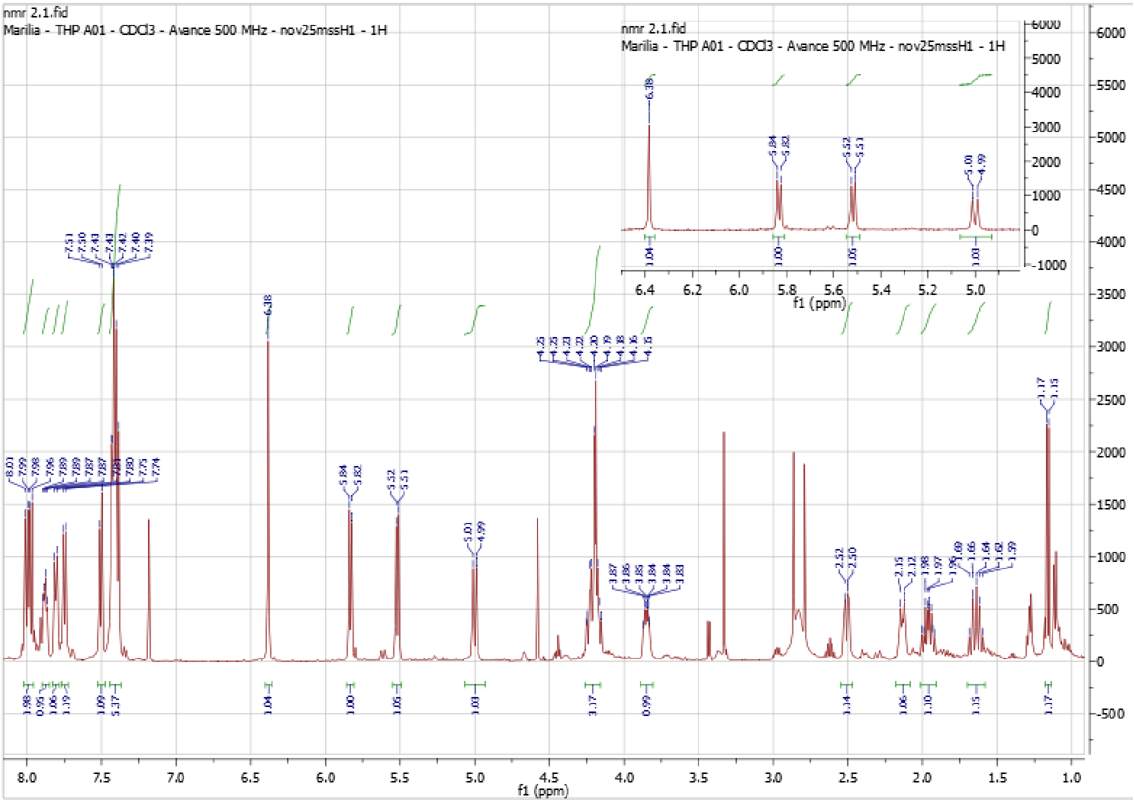

Figure S28. RMN ^{1}H spectrum of adduct **8c**.

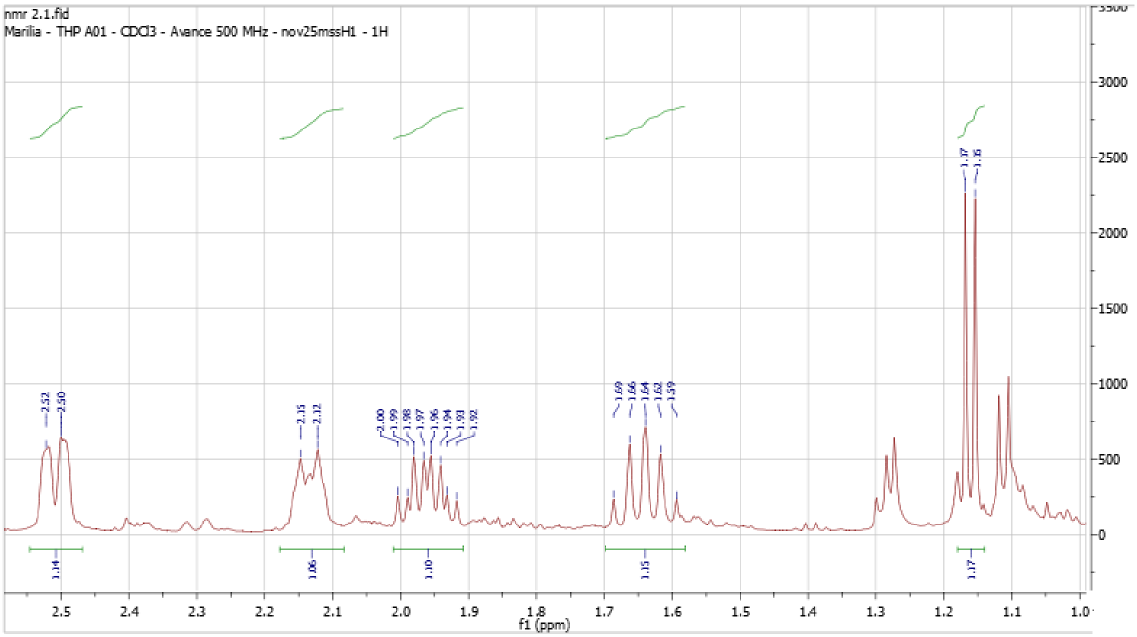

Figure S29. RMN ^{1}H spectrum enlargement of adduct **8c** (1.0 – 2.55ppm).

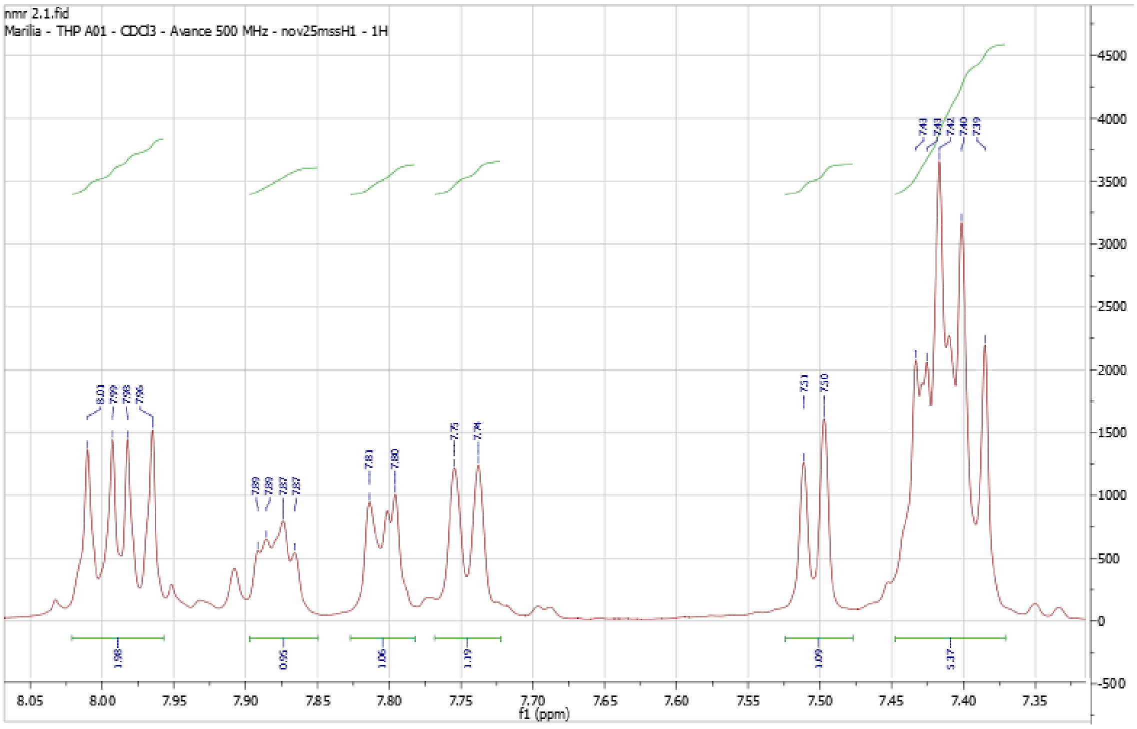

Figure S30. RMN ^{1}H spectrum enlargement of adduct **8c** (7.35 – 8.05ppm).

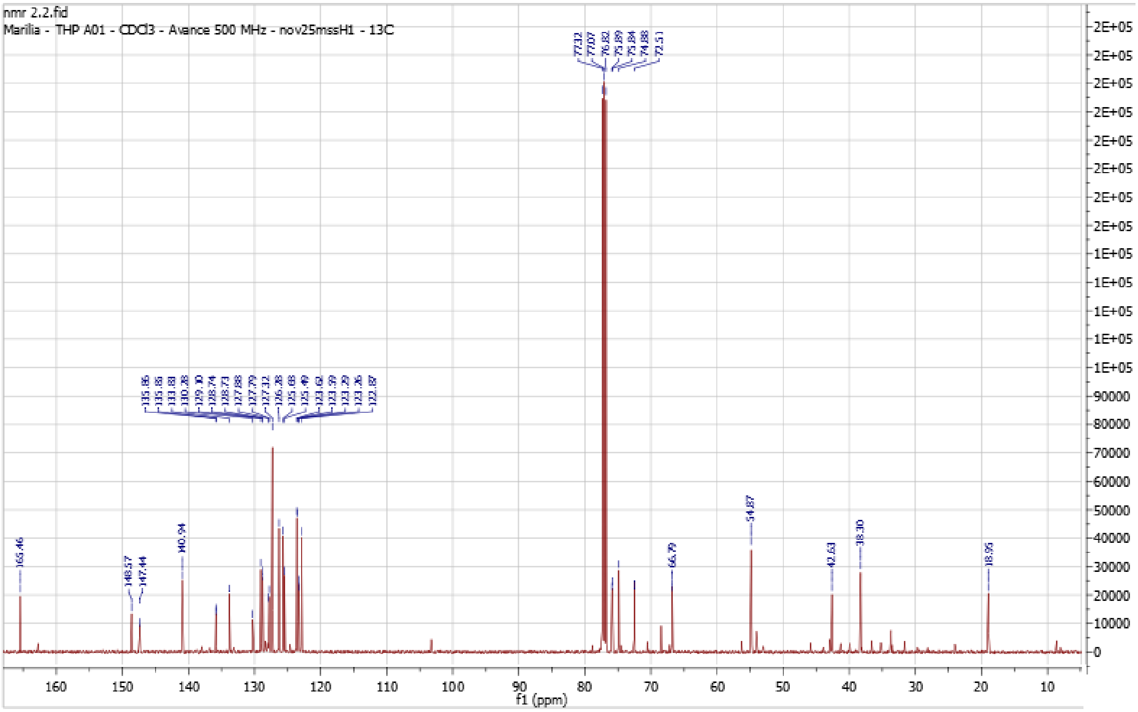

Figure S31. RMN ^{13}C spectrum of adduct **8c**.

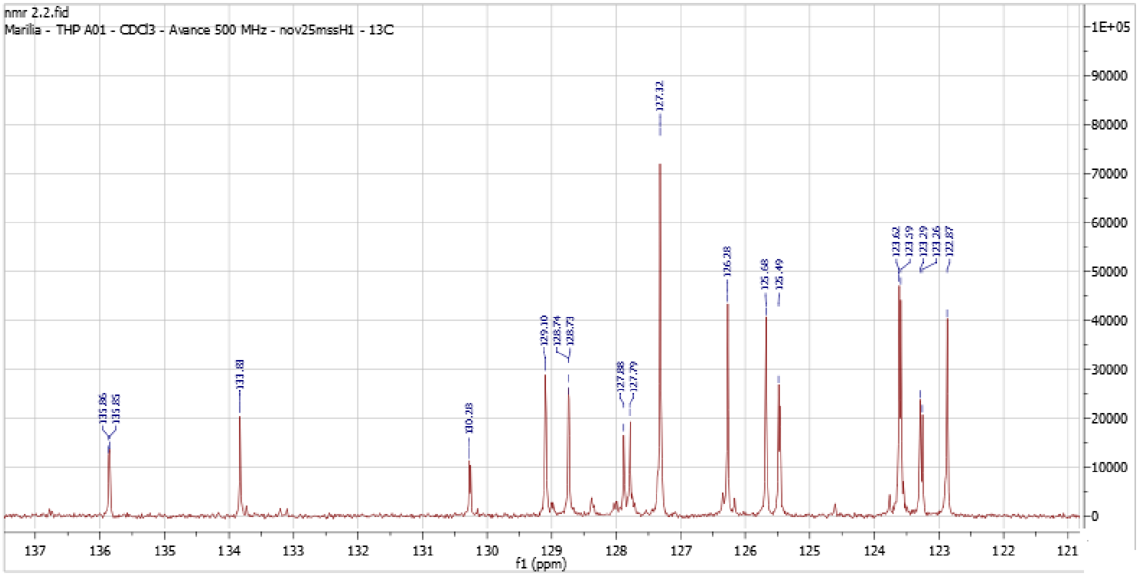

Figure S32. RMN ^{13}C spectrum enlargement of adduct **8c** (122 – 136ppm).

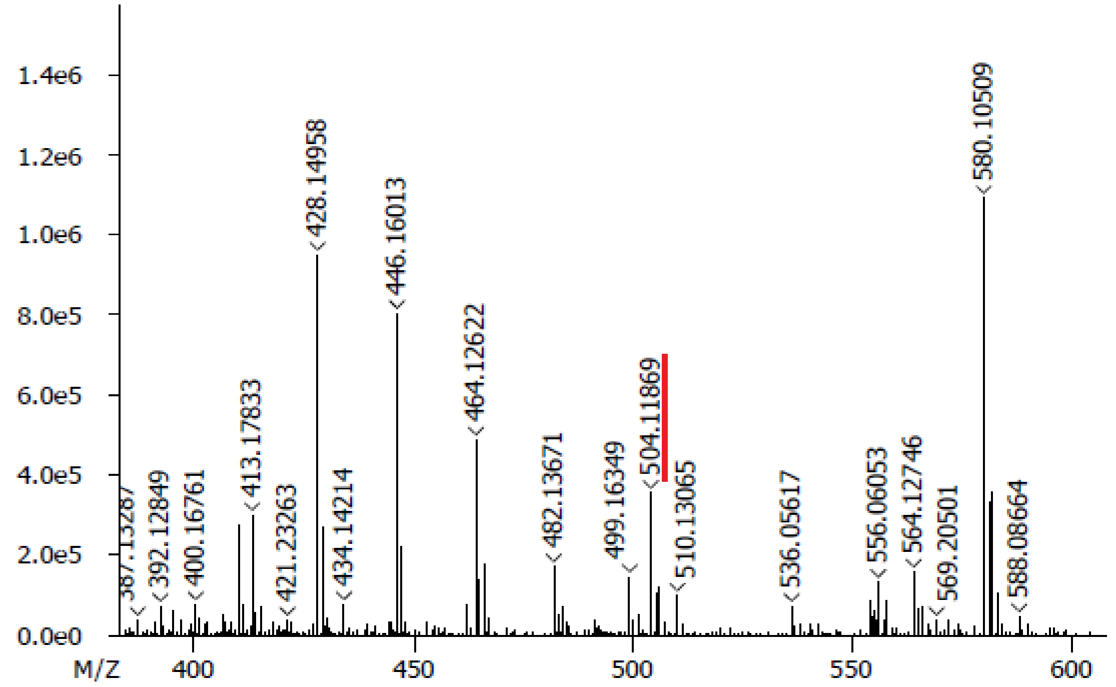

Figure S33. HRMS spectrum of compound **8c**.

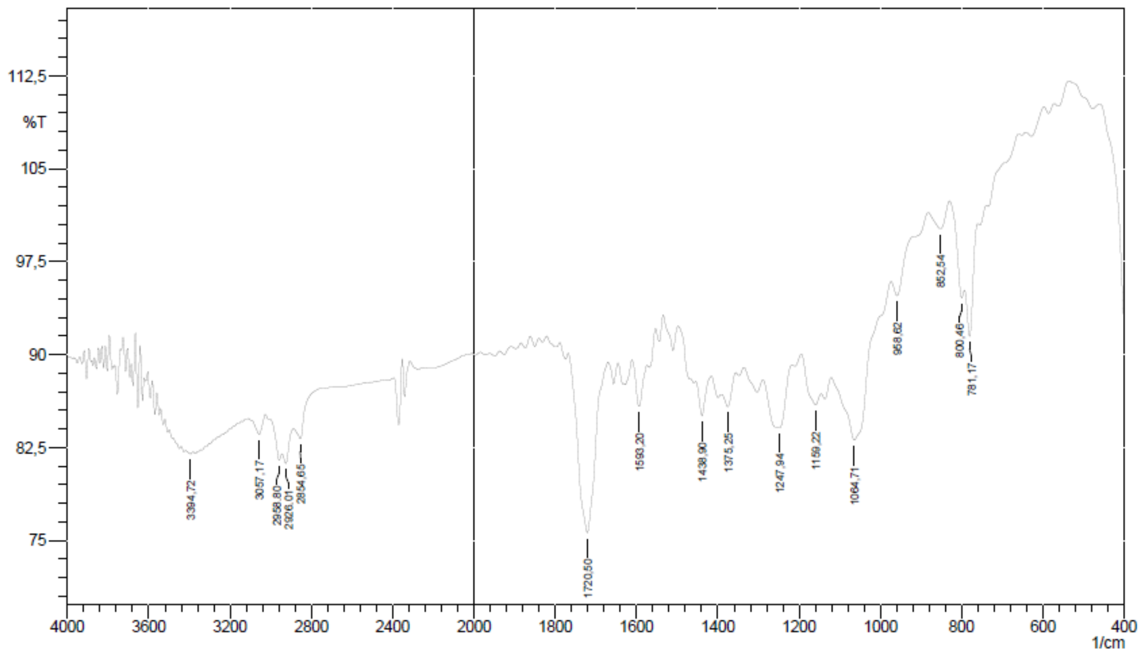

Figure S34. FTIR spectrum of adduct **8d**.

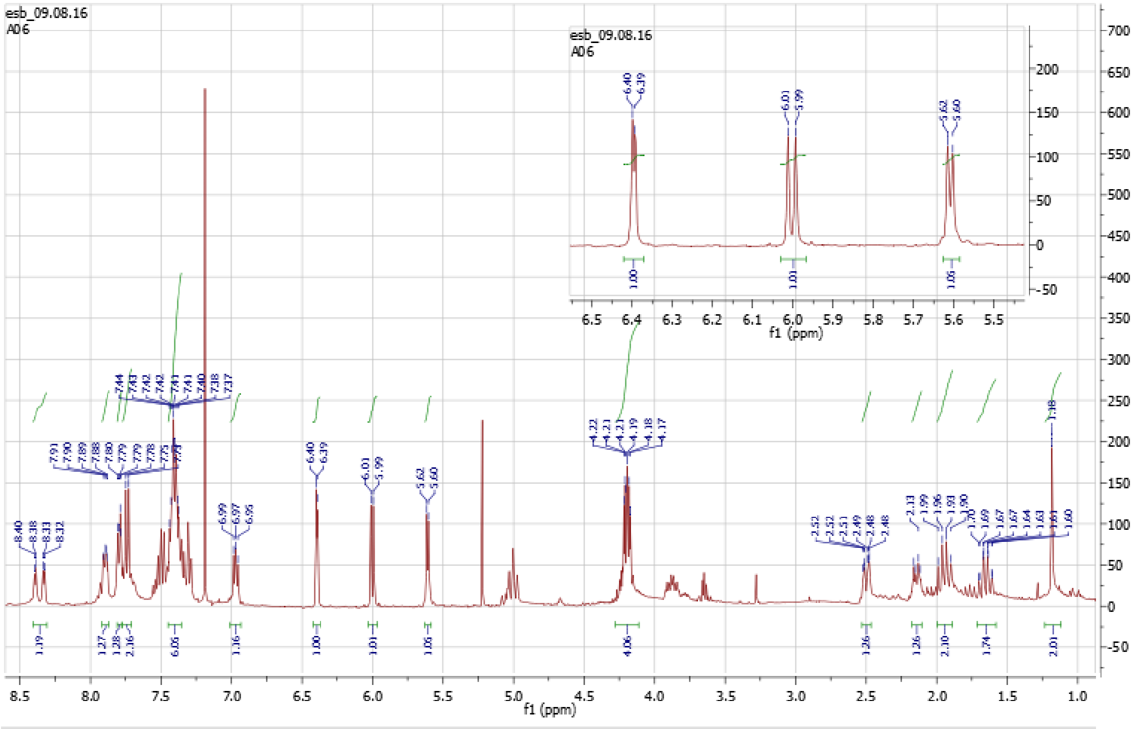

Figure S35. RMN ^{1}H spectrum of adduct **8d**.

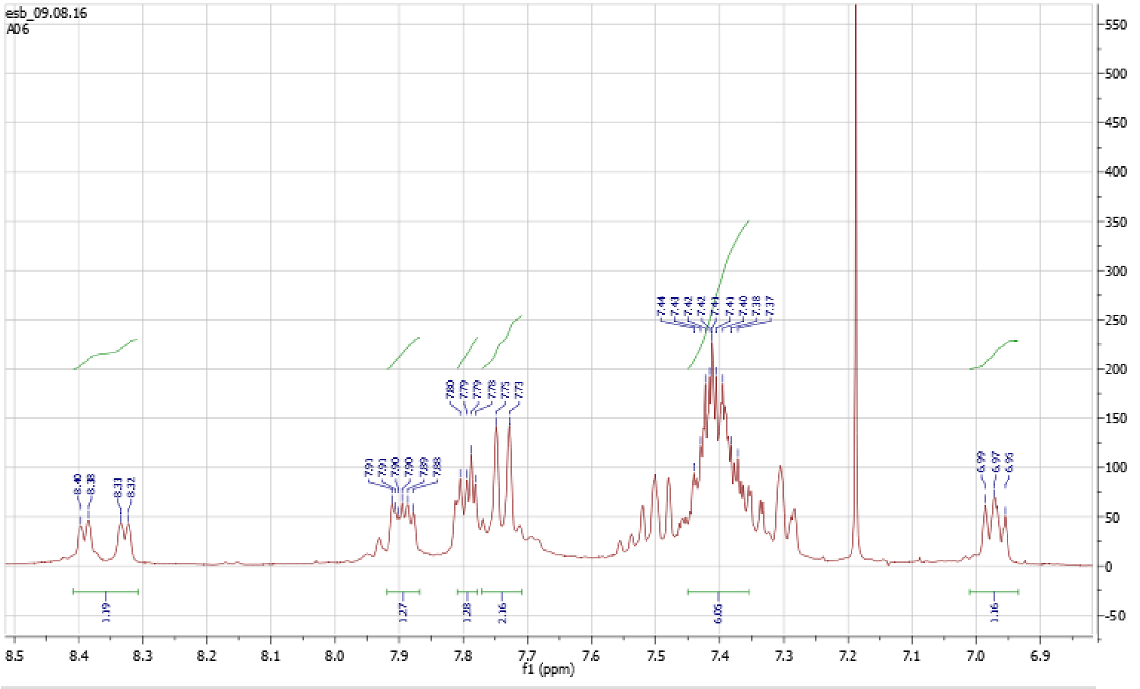

Figure S36. RMN ^{1}H spectrum enlargement of adduct **8d** (6.9 – 8.45ppm)

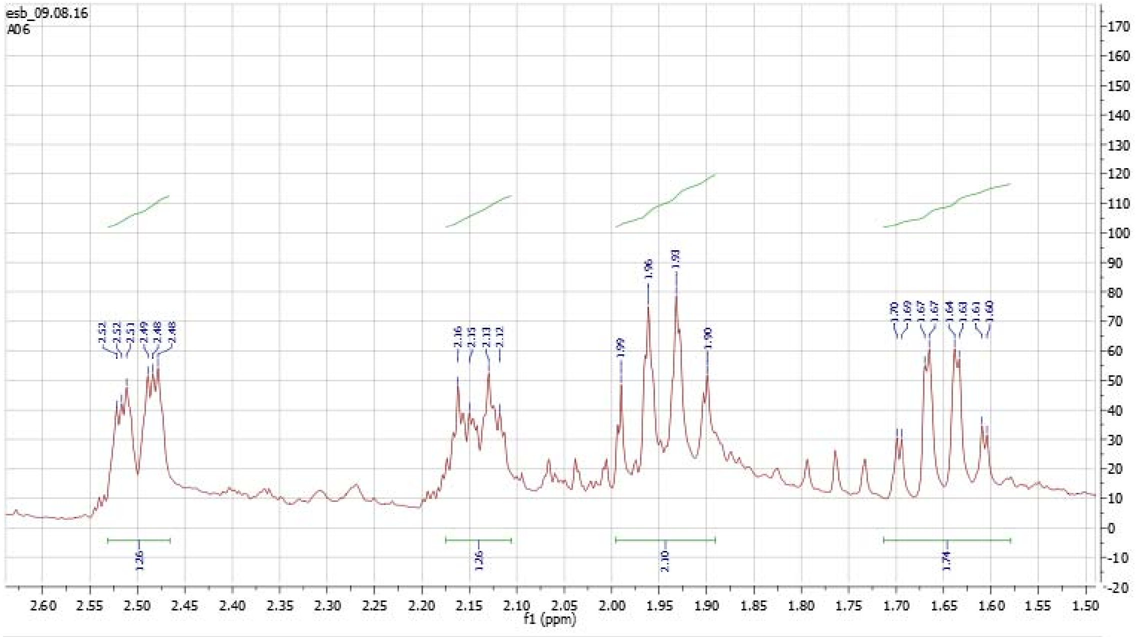

Figure S37. RMN ^{1}H specrum enlargement of adduct **8d** (1.55 – 2.55ppm).

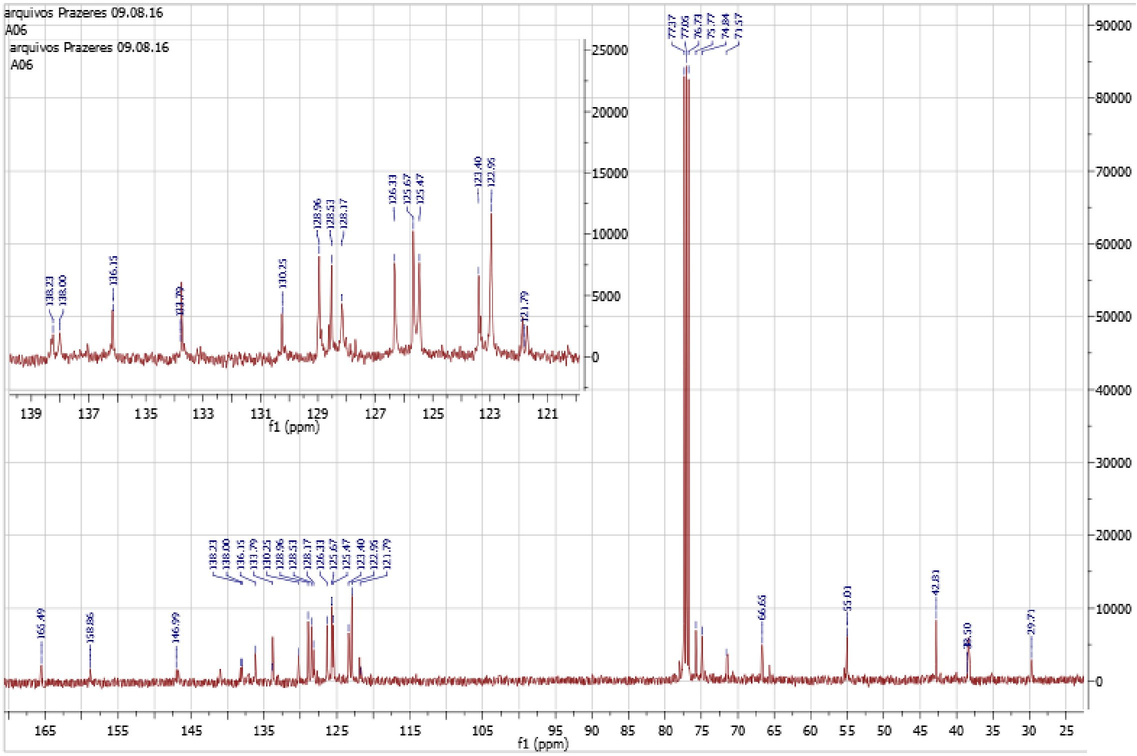

Figure S38. RMN ^{13}C spectrum of adduct **8d**.

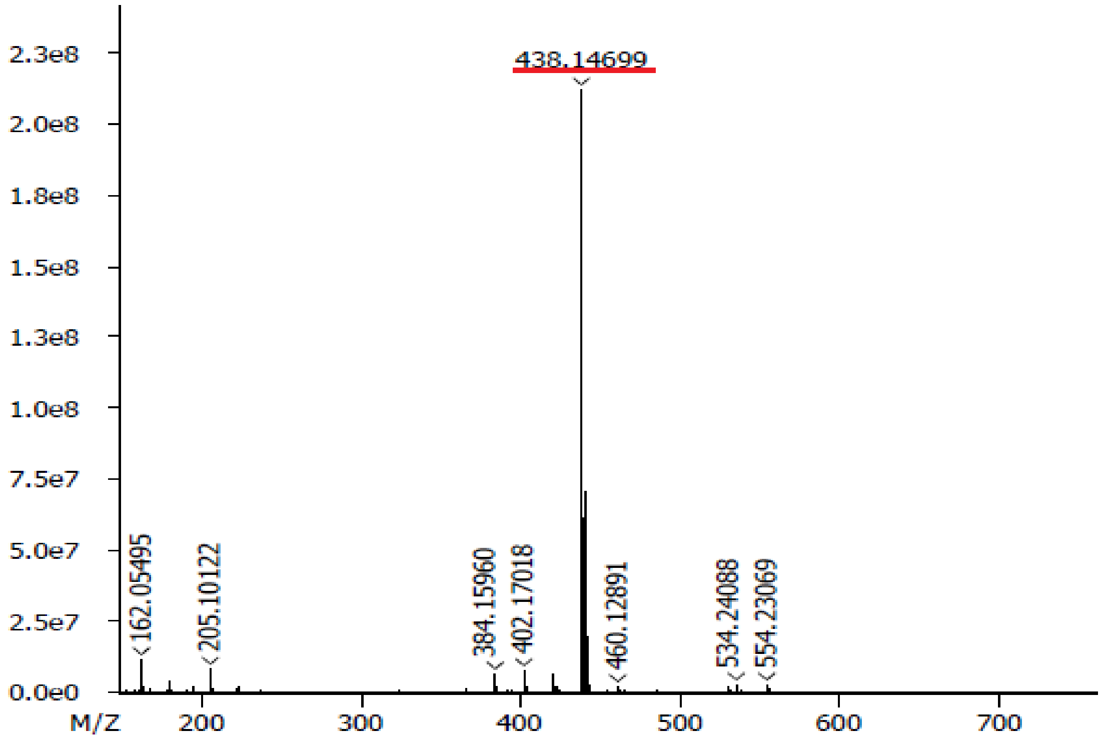

Figure S39. HRMS spectrum of compound **8d**.

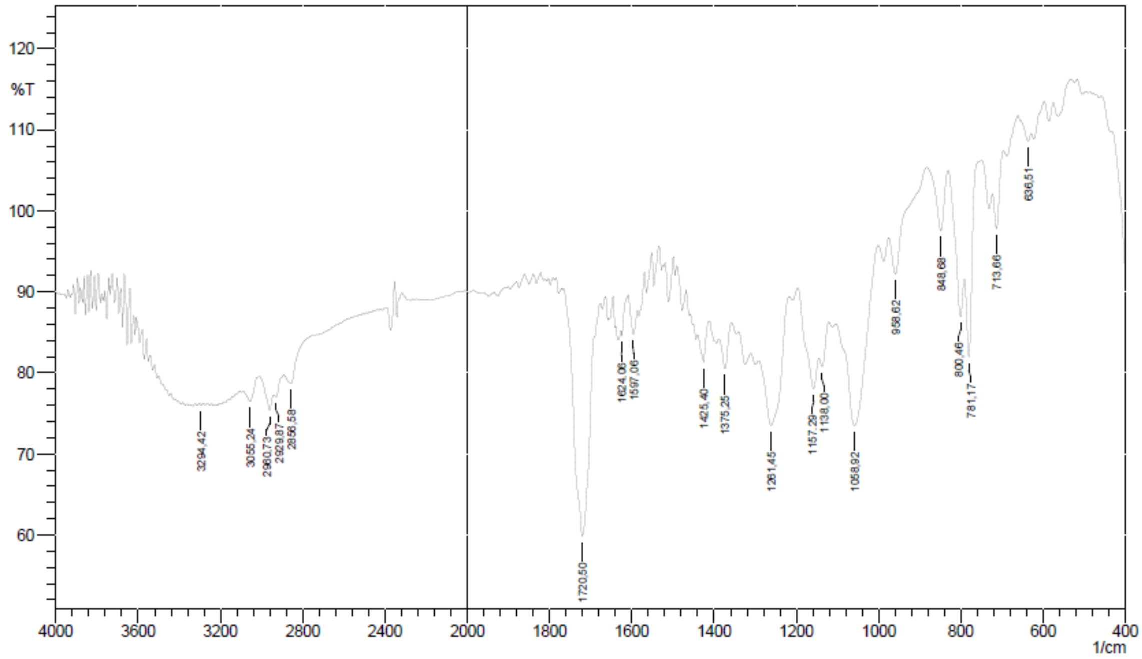

Figure S40. FTIR spectrum of adduct **8e**.

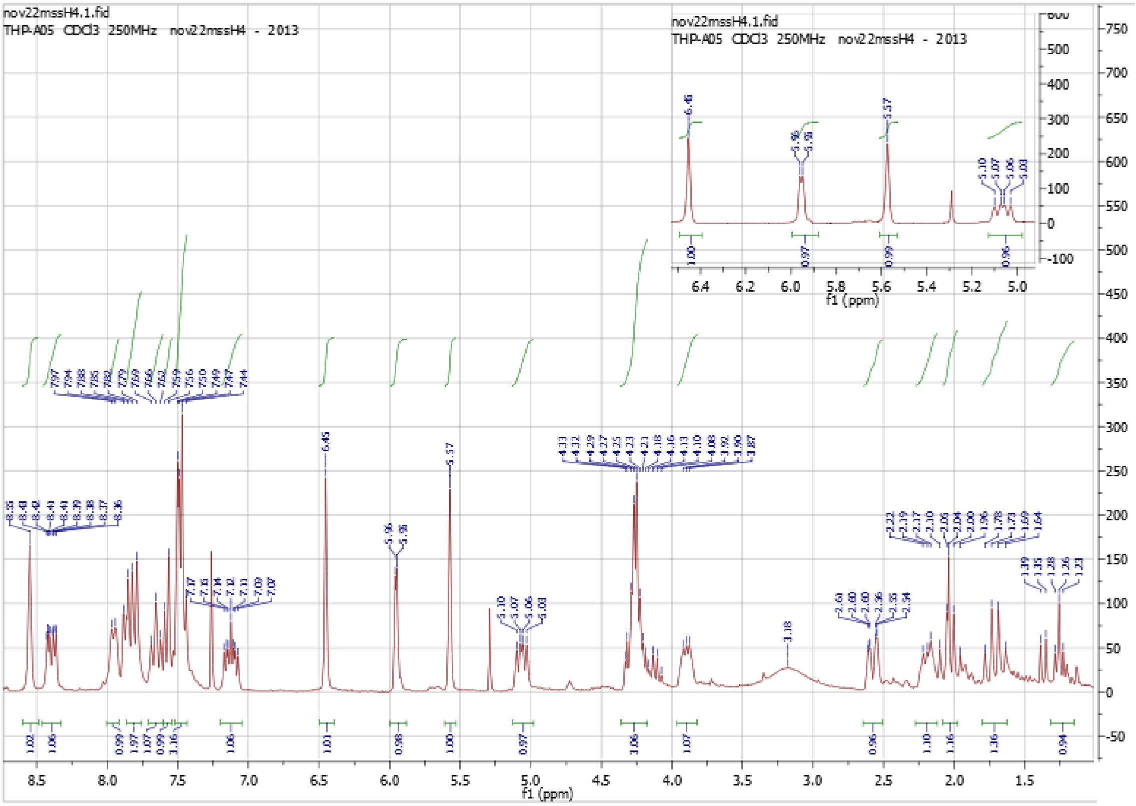

Figure S41. RMN ^{1}H spectrum of adduct **8e**.

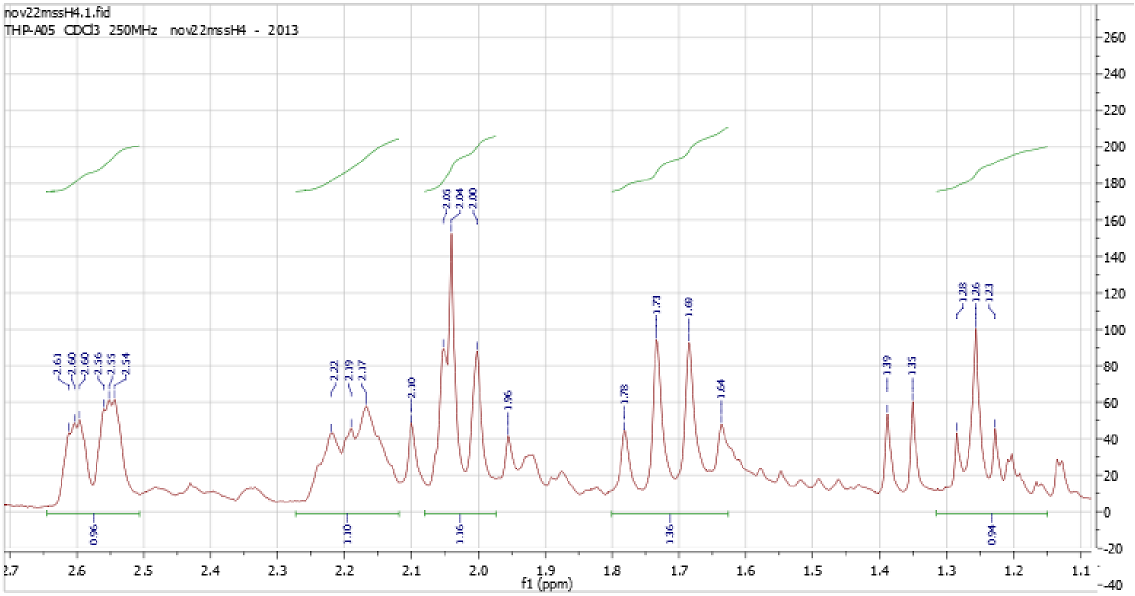

Figure S42. RMN ^{1}H spectrum enlargement of adduct **8e** (1.1 – 2.65ppm).

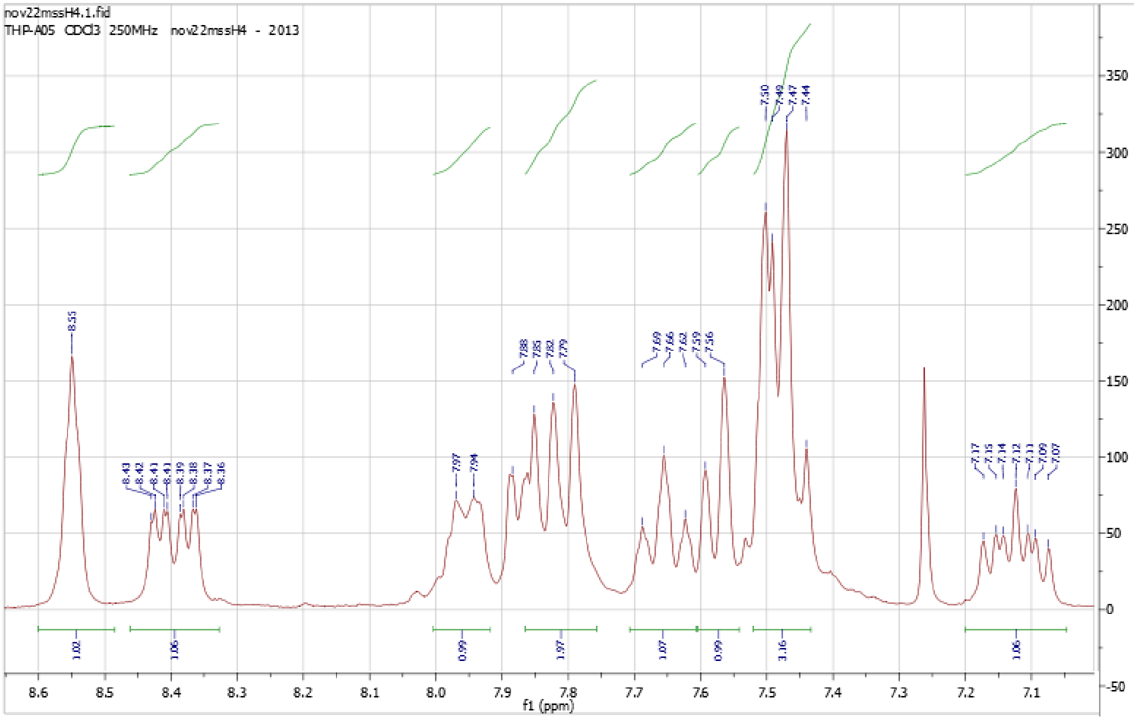

Figure S43. RMN ^{1}H spectrum enlargement of adduct **8e** (7.0 – 8.6ppm).

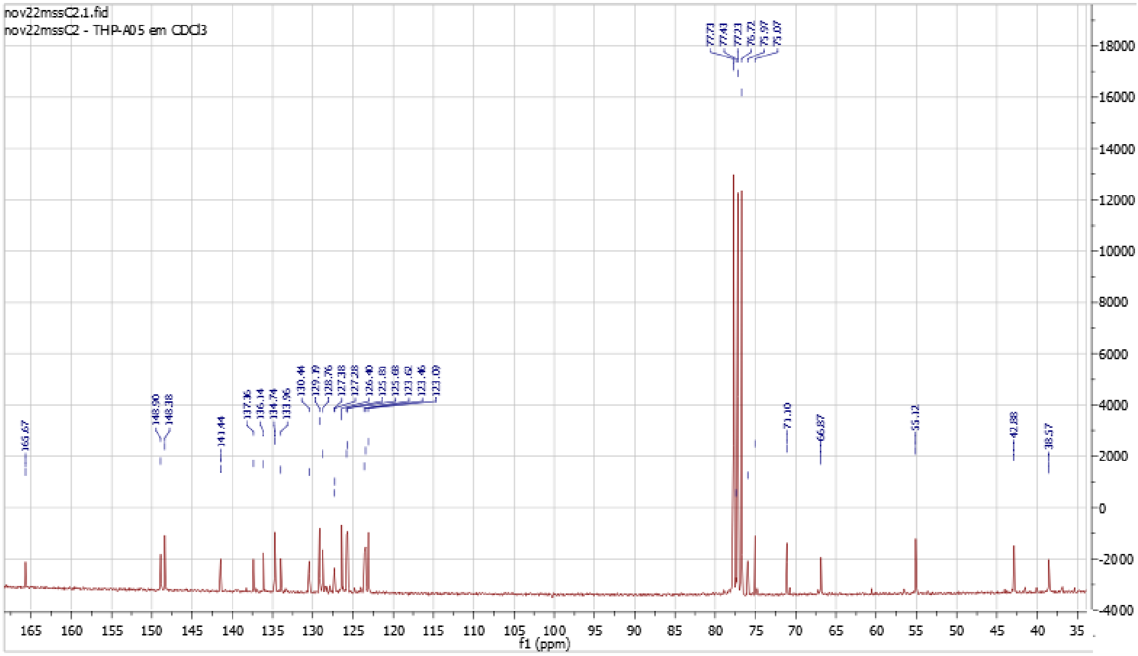

Figure S44. RMN ^{13}C spectrum of adduct **8e**.

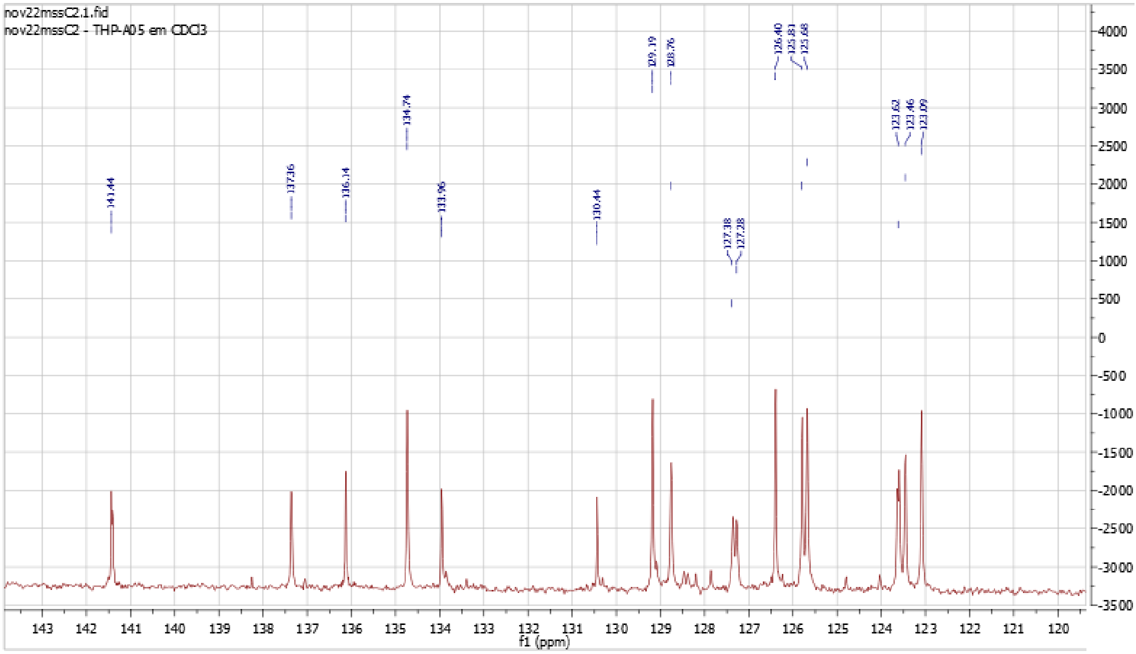

Figure S45. RMN ^{13}C spectrum enlargement of adduct **8e** (122 – 142ppm).

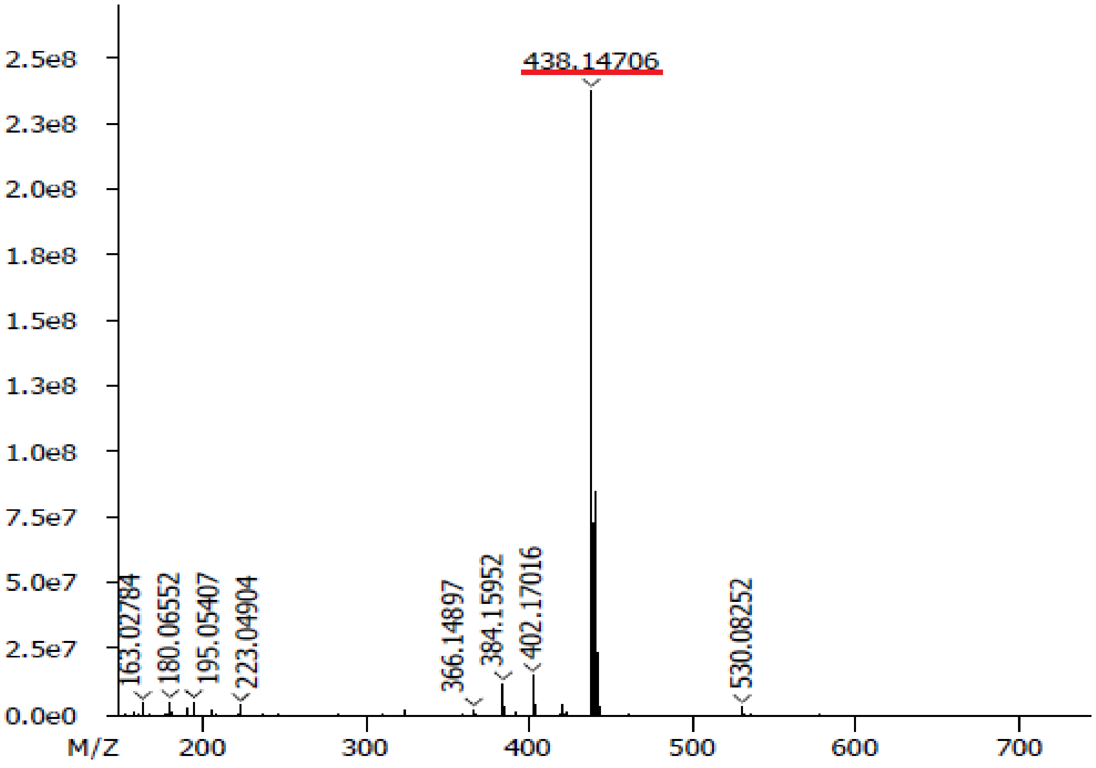

Figure S46. HRMS spectrum of compound **8e**.

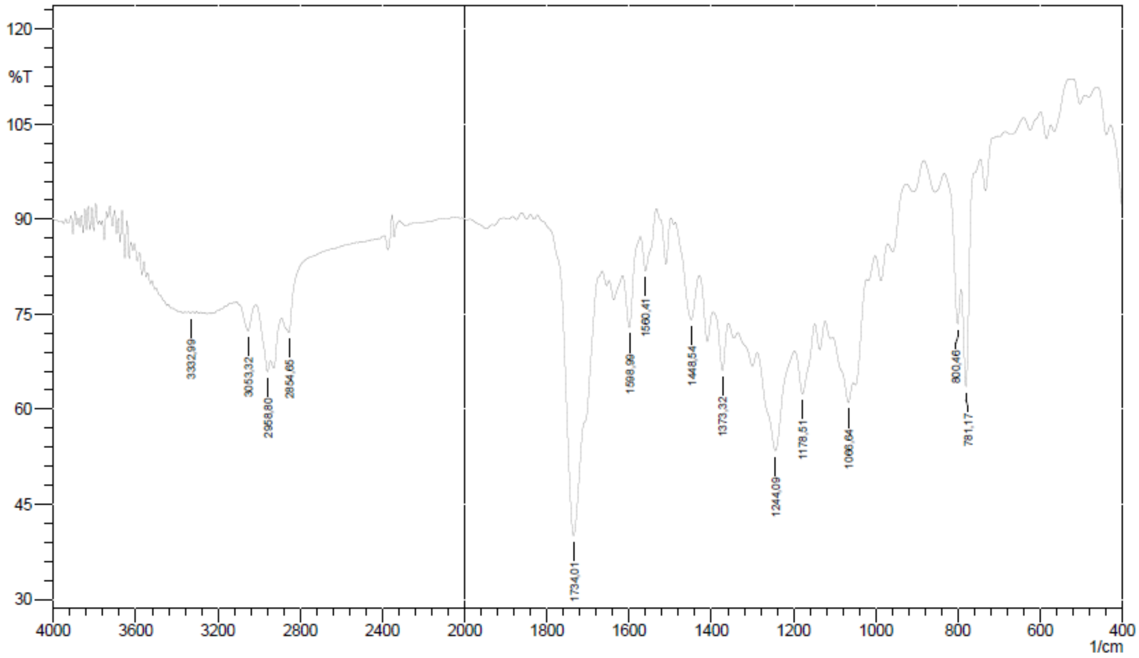

Figure S47. FTIR spectrum of adduct **8f**.

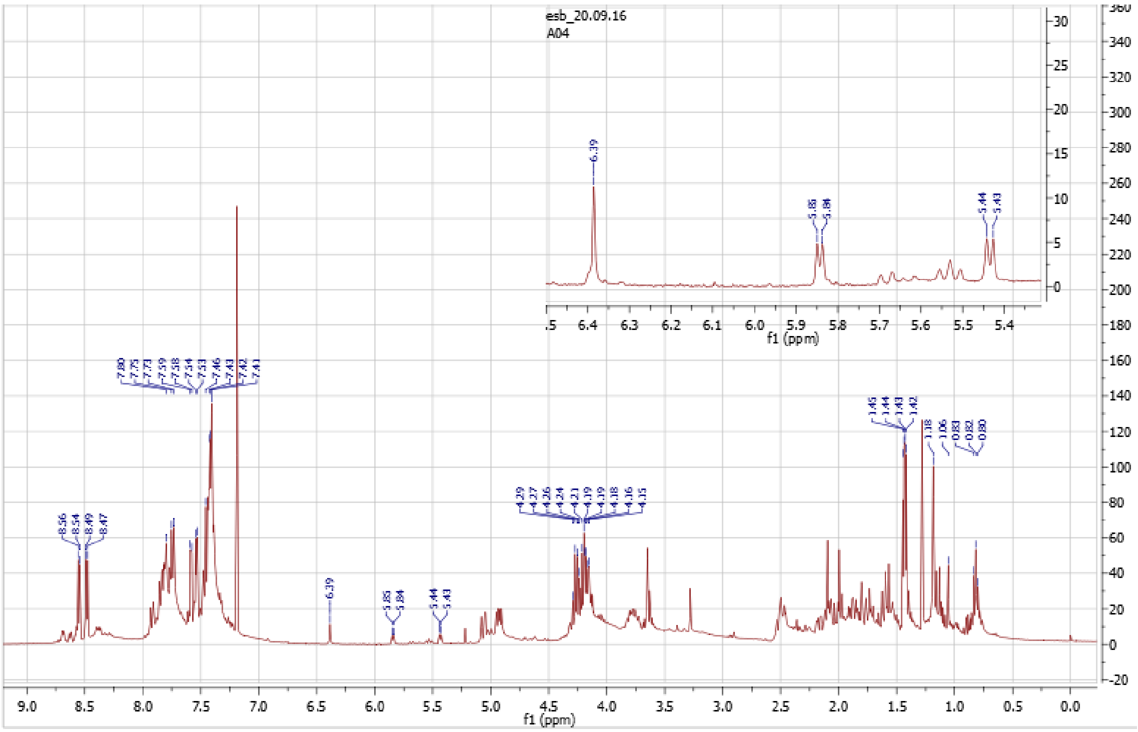

Figure S48. RMN ^{1}H Spectrum of adduct **8f**.

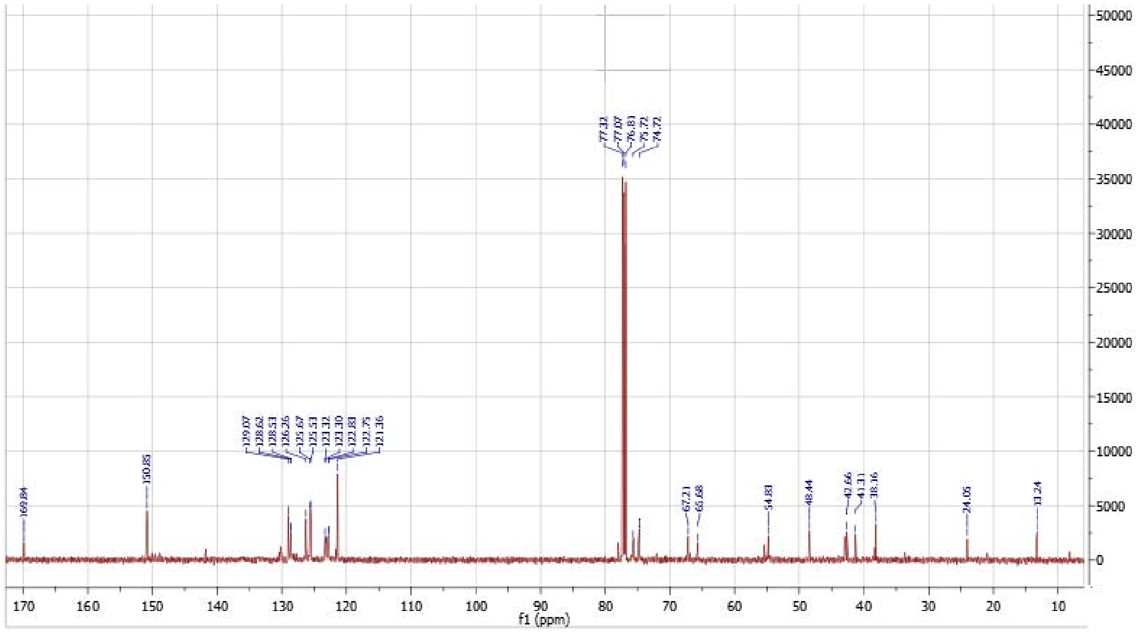

Figure S49. RMN ^{13}C spectrum of adduct **8f**.

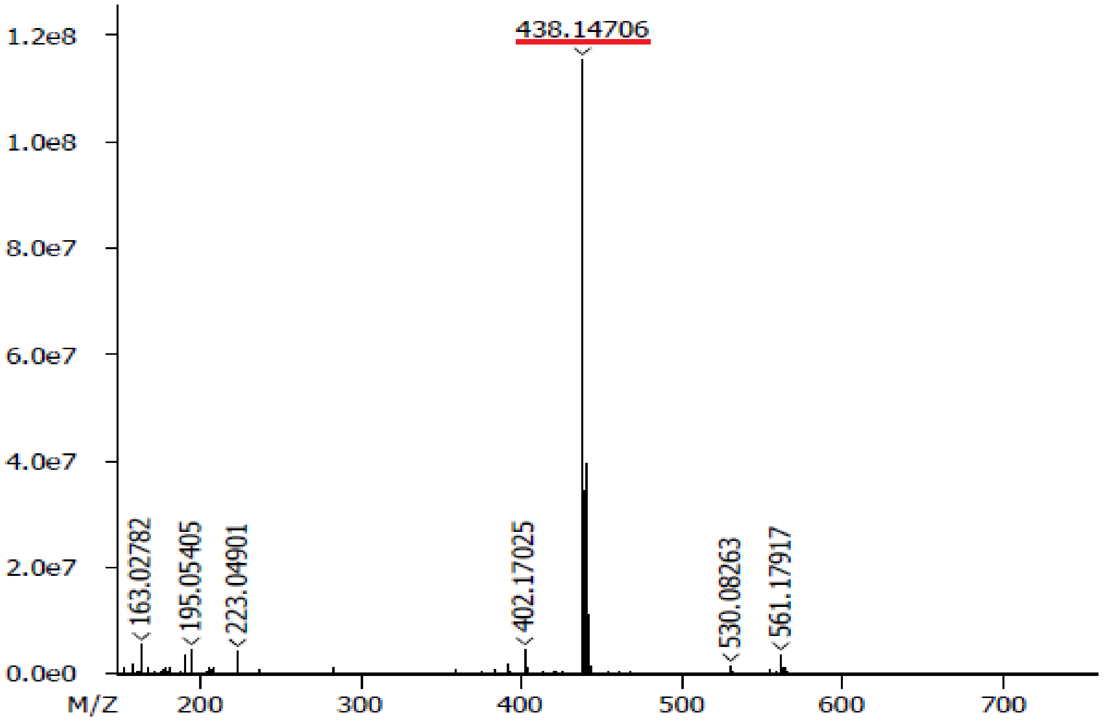

Figure S50. HRMS spectrum of compound **8f**.

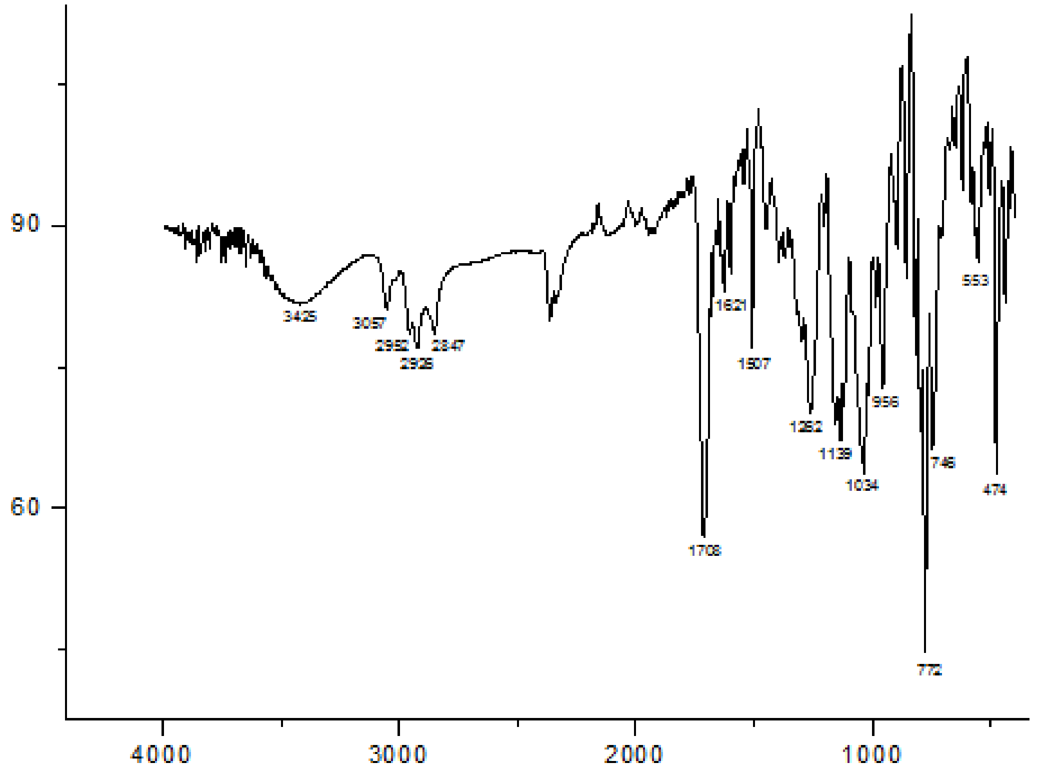

Figure S51. FTIR spectrum of adduct **8g**.

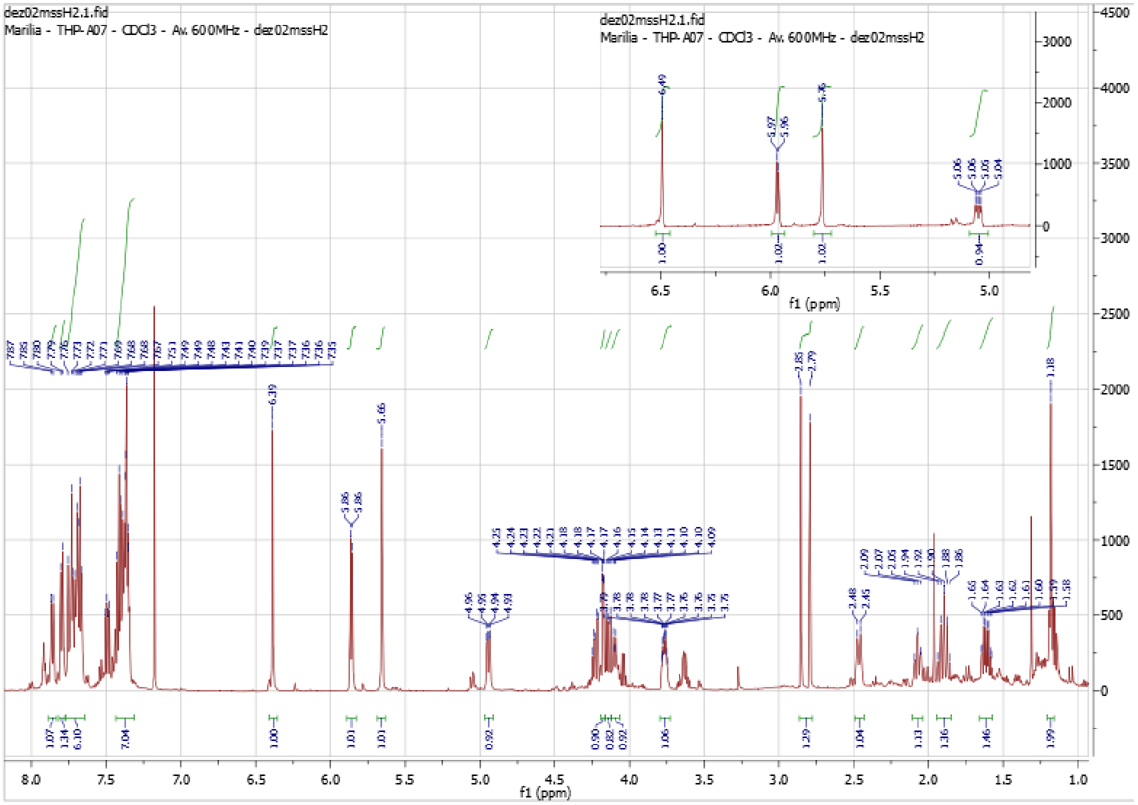

Figure S52. RMN ^{1}H spectrum of adduct **8g**.

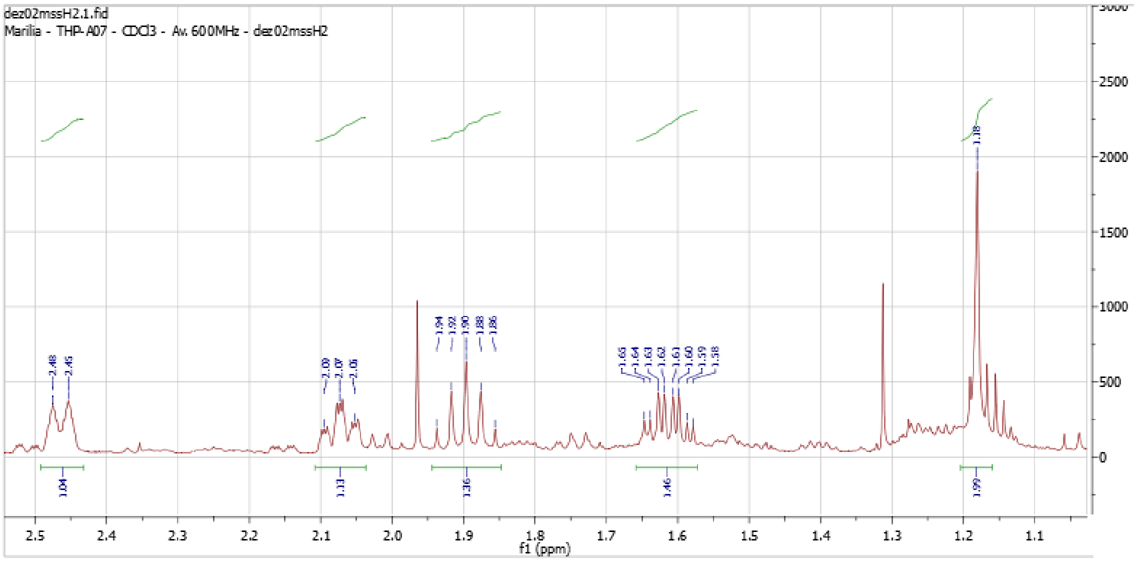

Figure S53. RMN ^{1}H spectrum enlargement of adduct **8g** (1.1 – 2.5ppm).

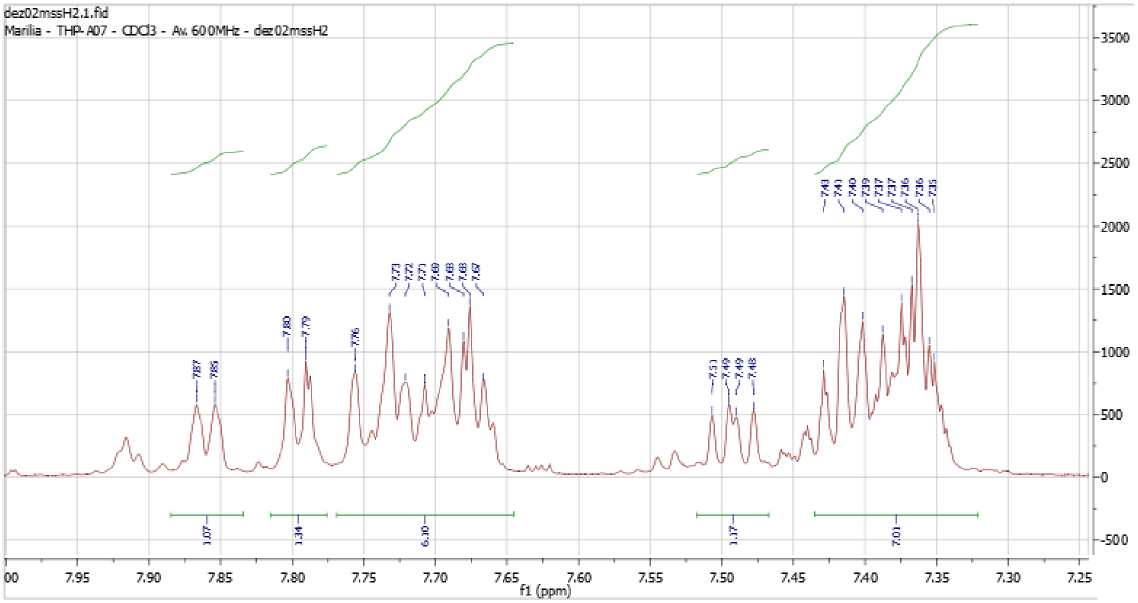

Figure S54. RMN ^{1}H spectrum enlargement of adduct **8g** (7.3 – 7.9ppm).

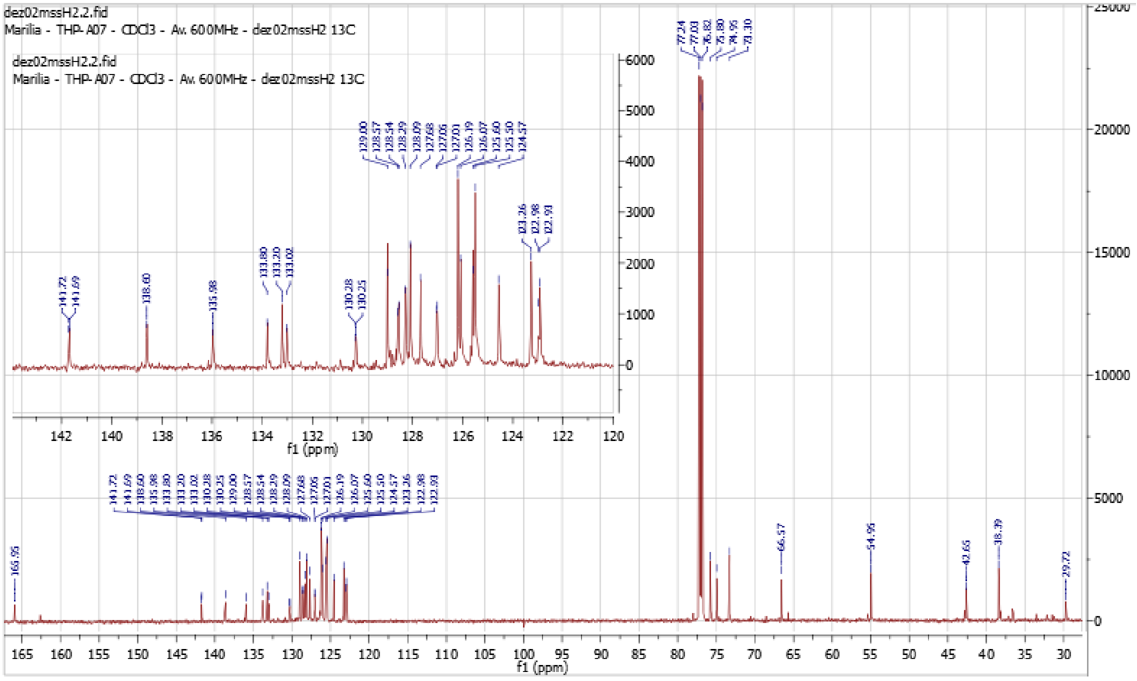

Figure S55. RMN ^{13}C spectrum of adduct **8g.**

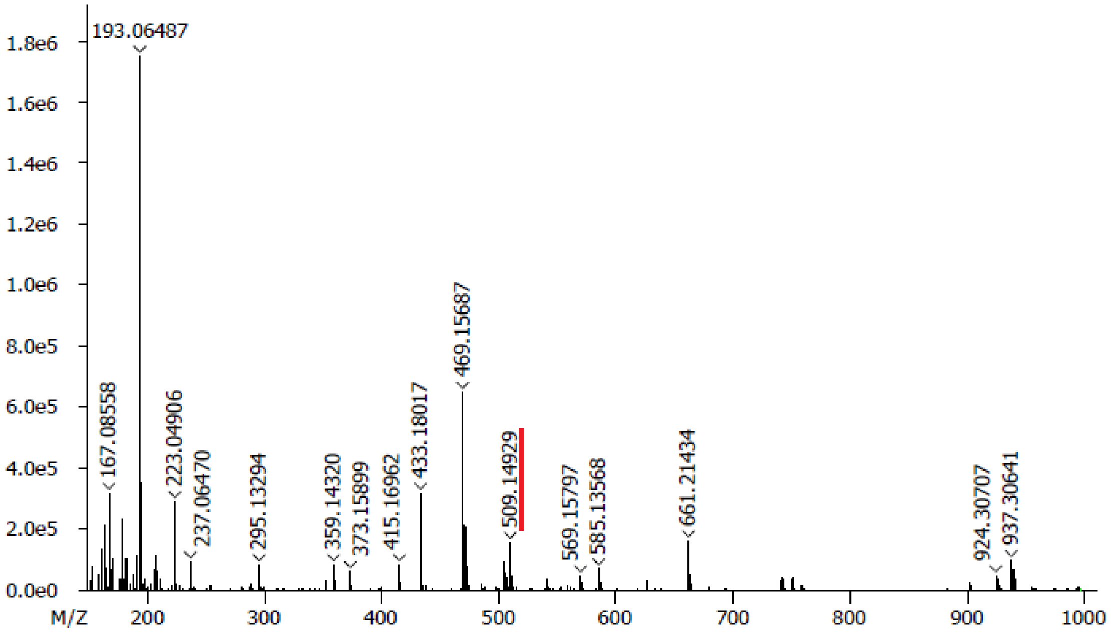

Figure S56. HRMS spectrum of compound **8g**.

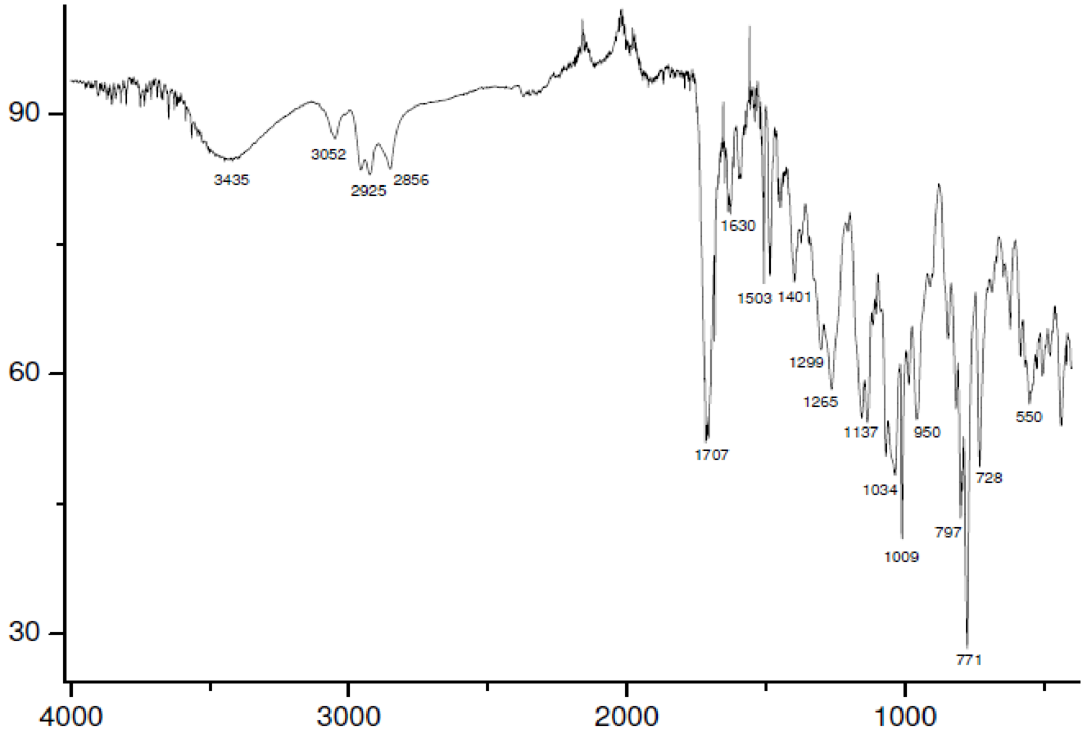

Figure S57. FTIR spectrum of adduct **8h**.

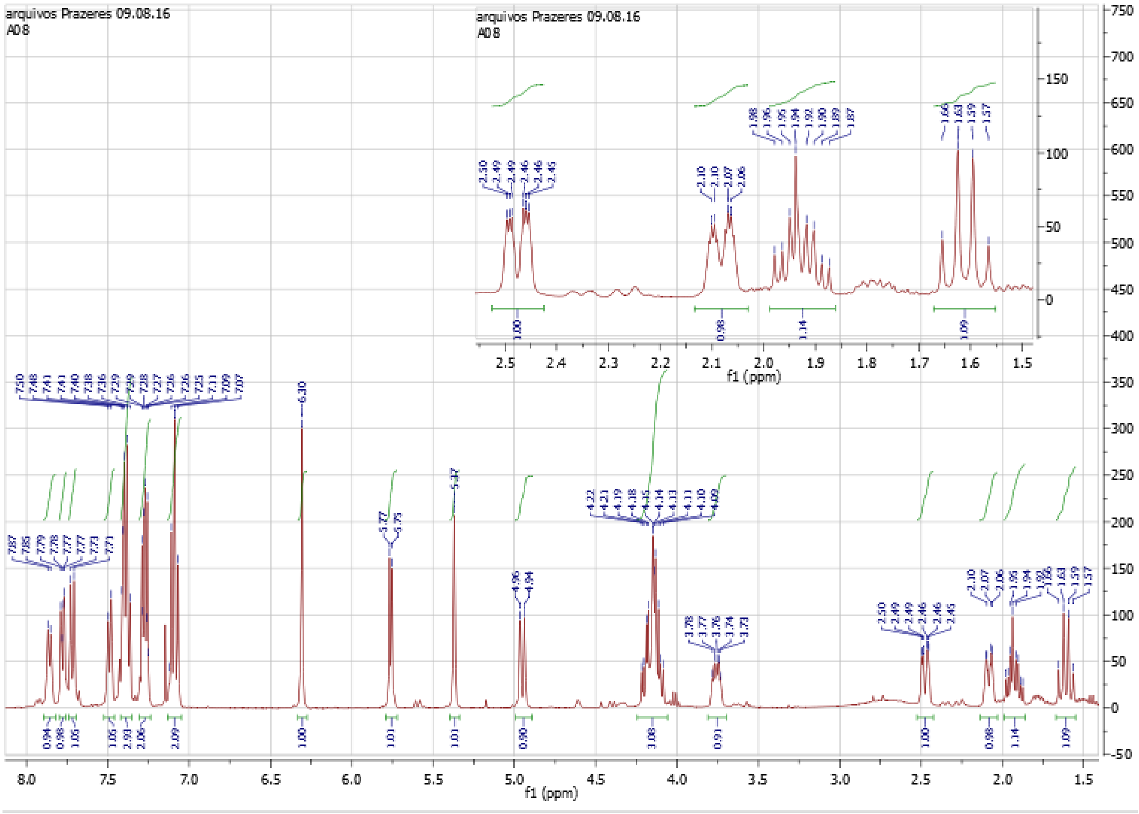

Figure S58. RMN ^{1}H spectrum of adduct **8h**.

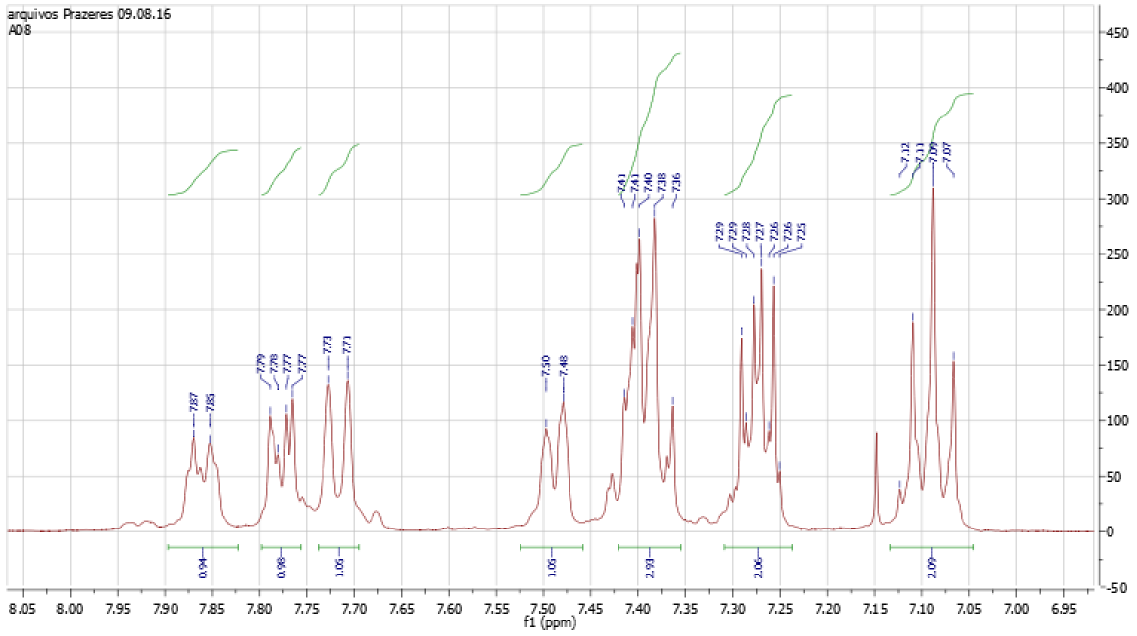

Figure S59. RMN ¹H spectrum enlargement of adduct **8h** (7.05 – 7.9ppm).

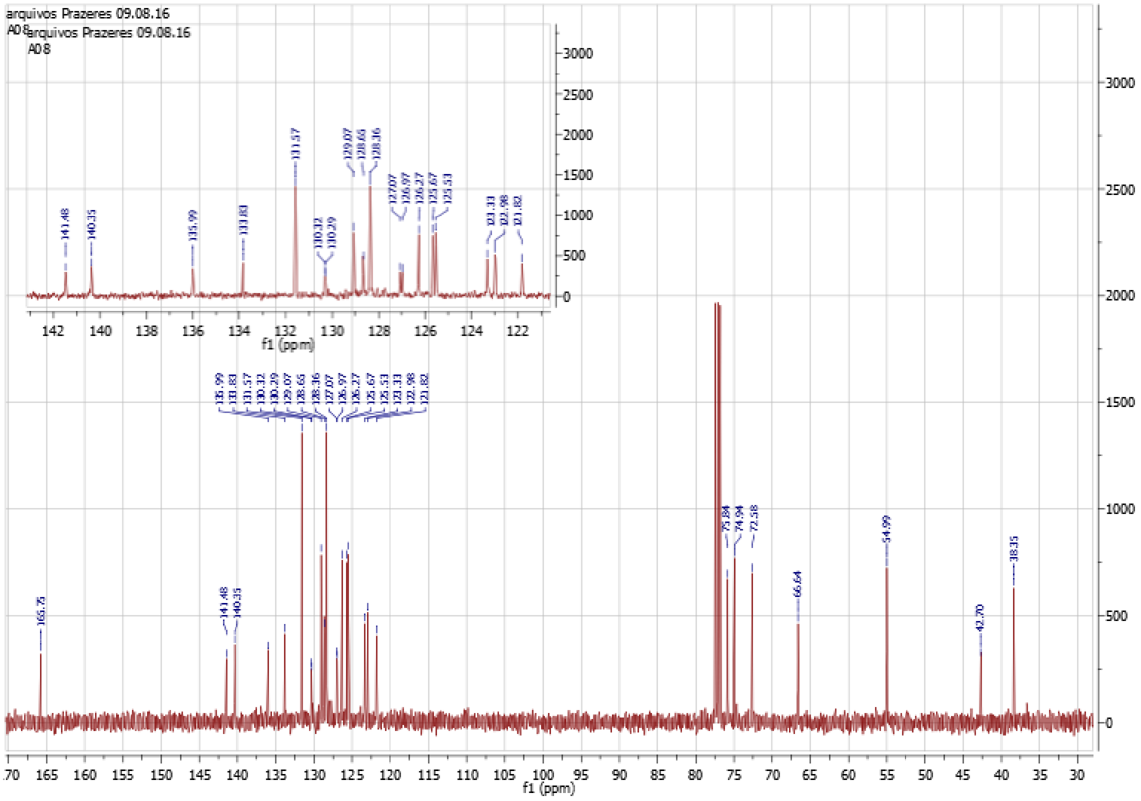

Figure S60. RMN ¹³C spectrum of adduct **8h**.

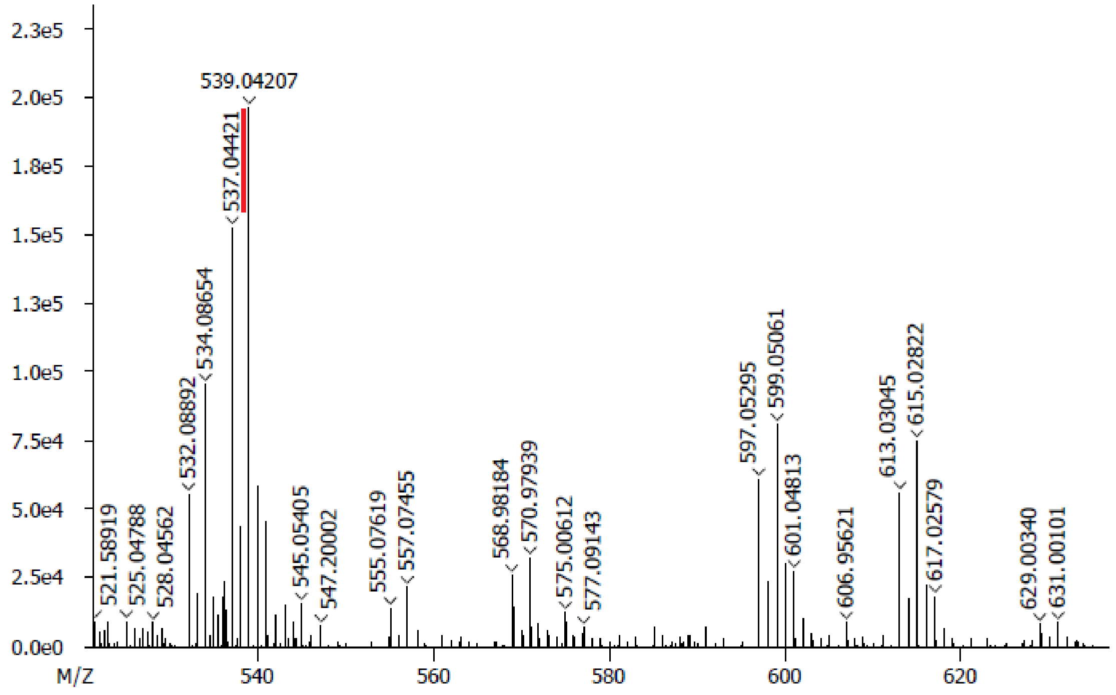

Figure S61. HRMS spectrum of compound **8h**.

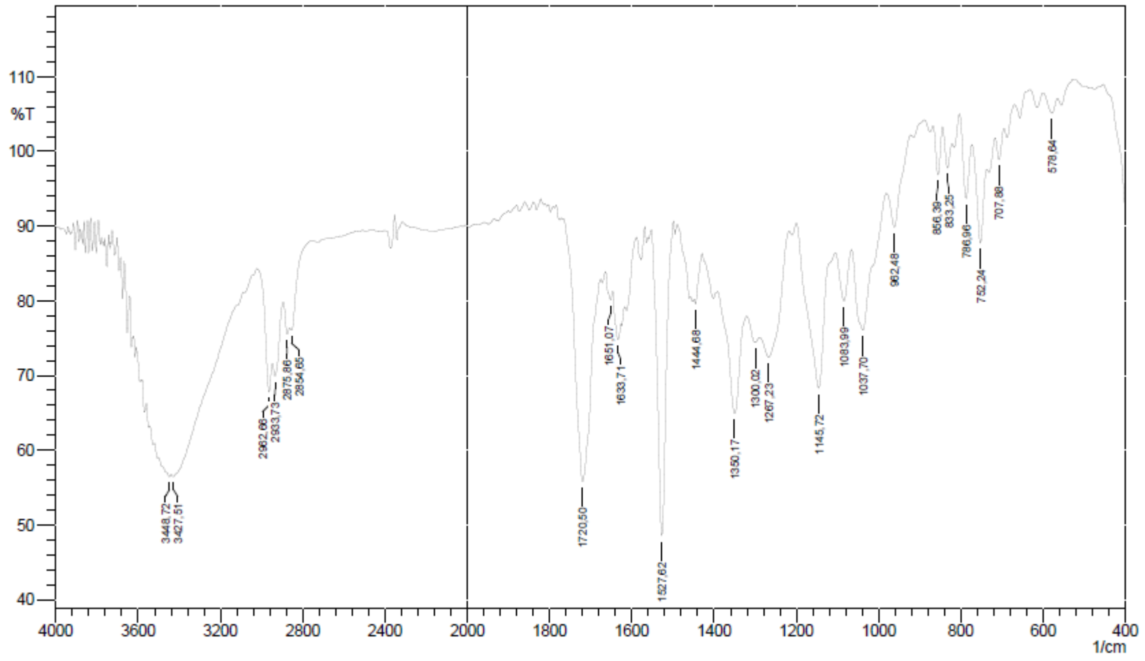

Figure S62. FTIR spectrum of adduct **9a**.

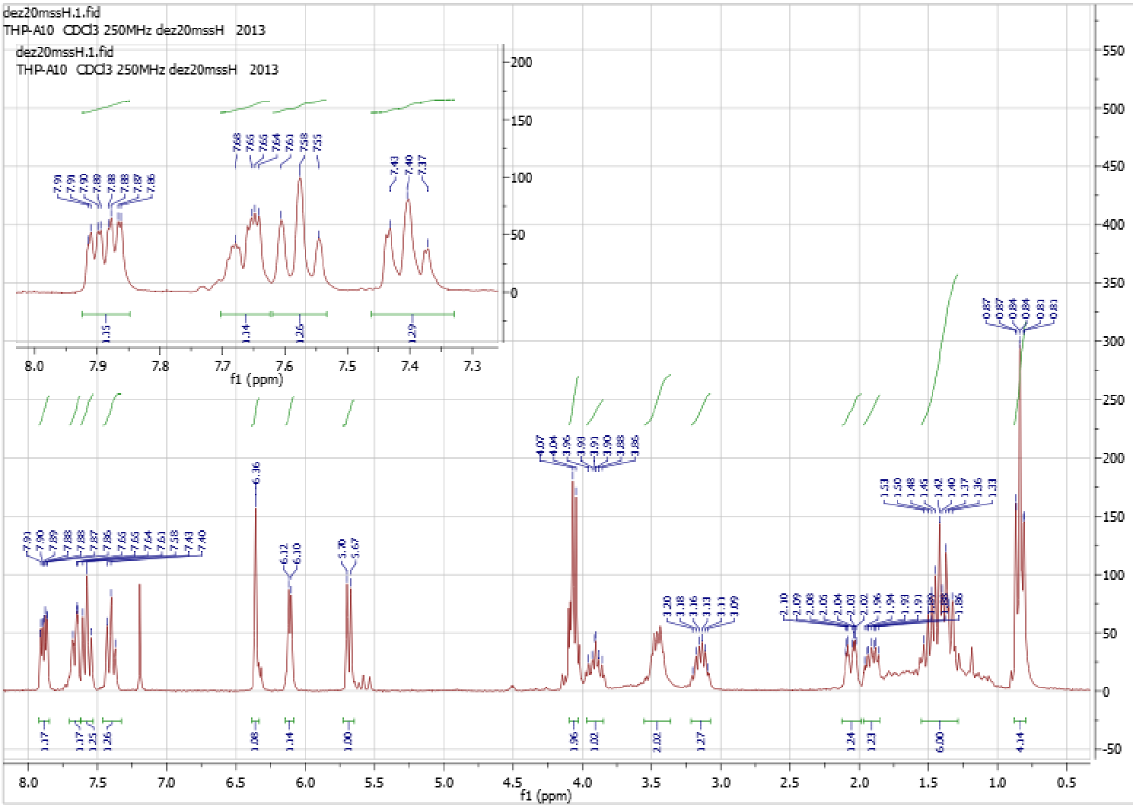

Figure S63. RMN ^{1}H spectrum of adduct **9a**.

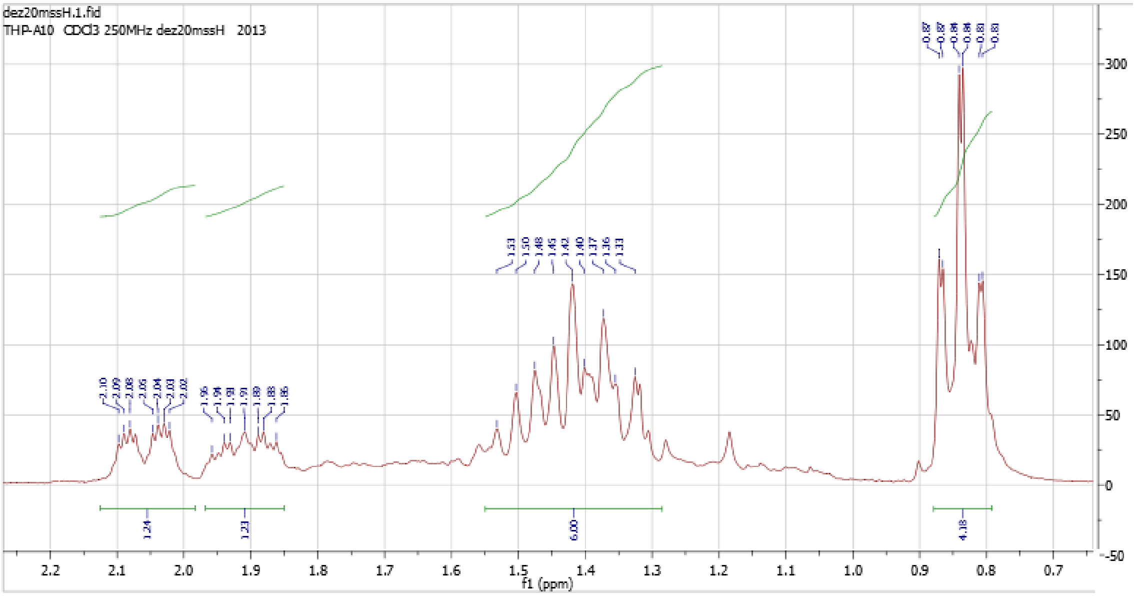

Figure S64. RMN ^{1}H spectrum enlargement of adduct **9a** (0.7 – 2.2ppm).

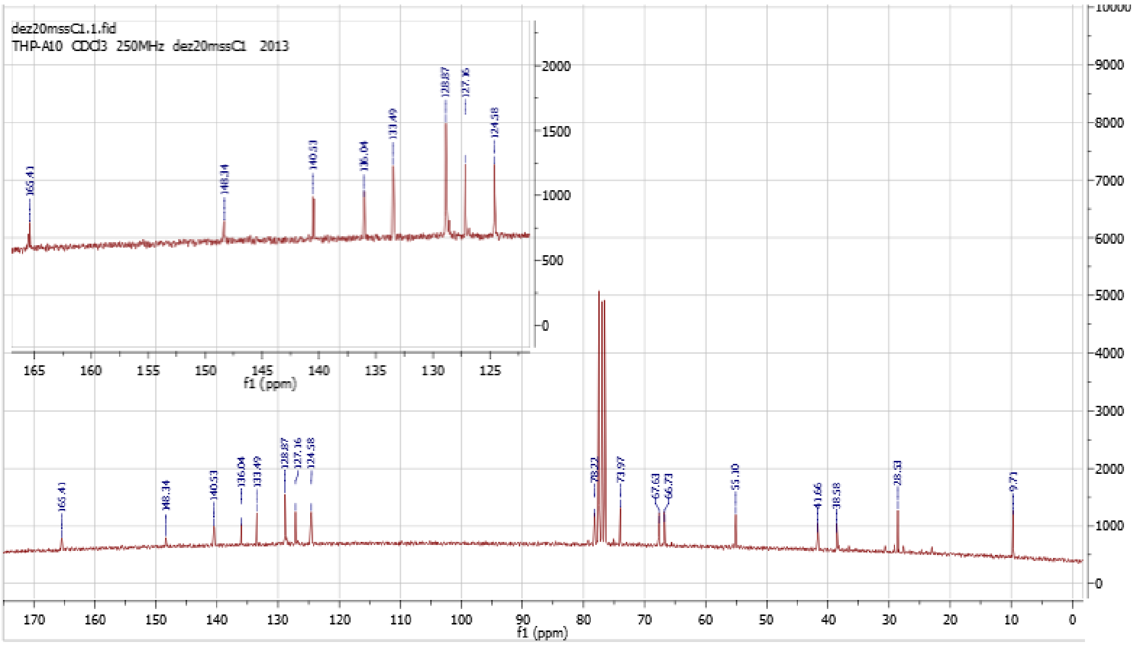

Figure S65. RMN ^{13}C spectrum of adduct **9a**.

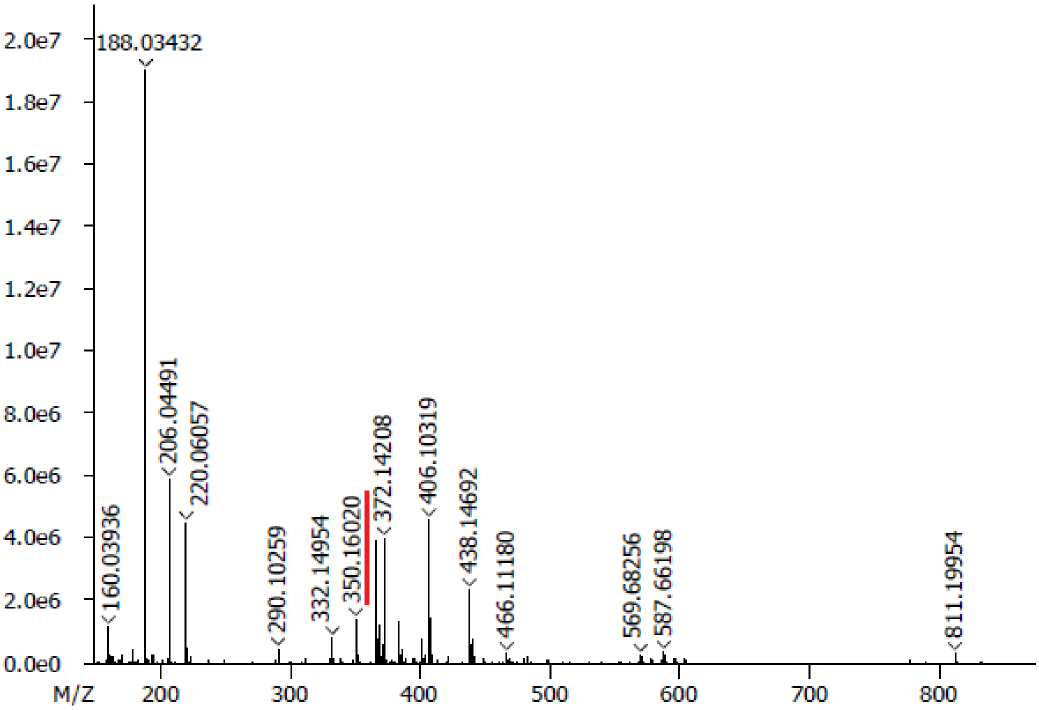

Figure S66. HRMS spectrum of compound **9a**.

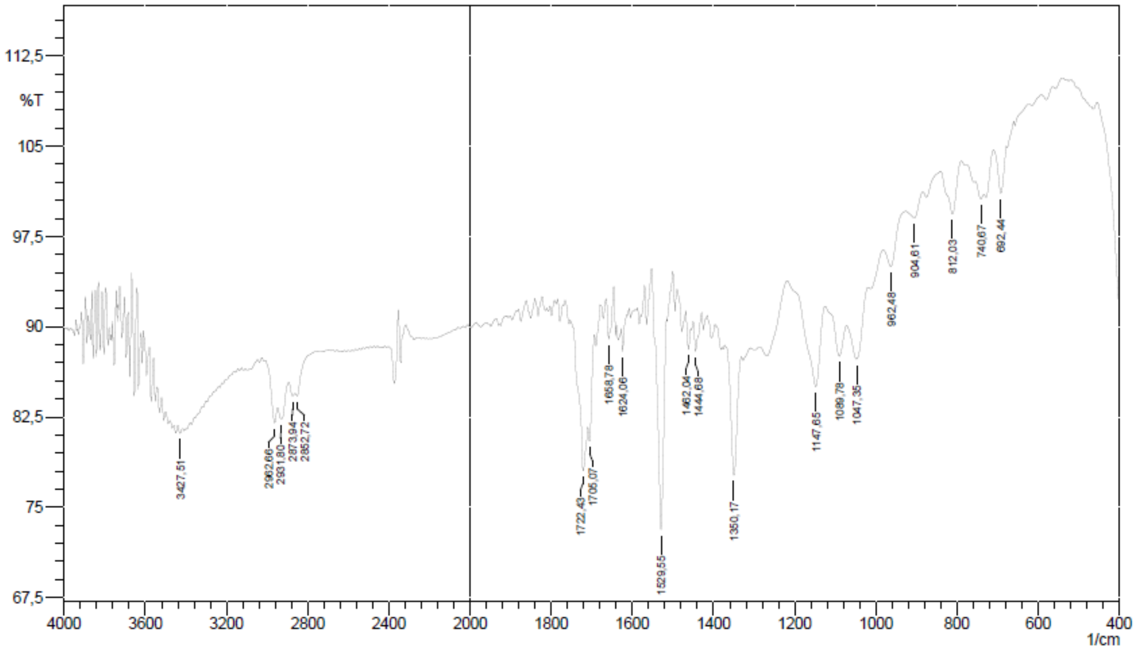

Figure S67. FTIR spectrum of adduct **9b**.

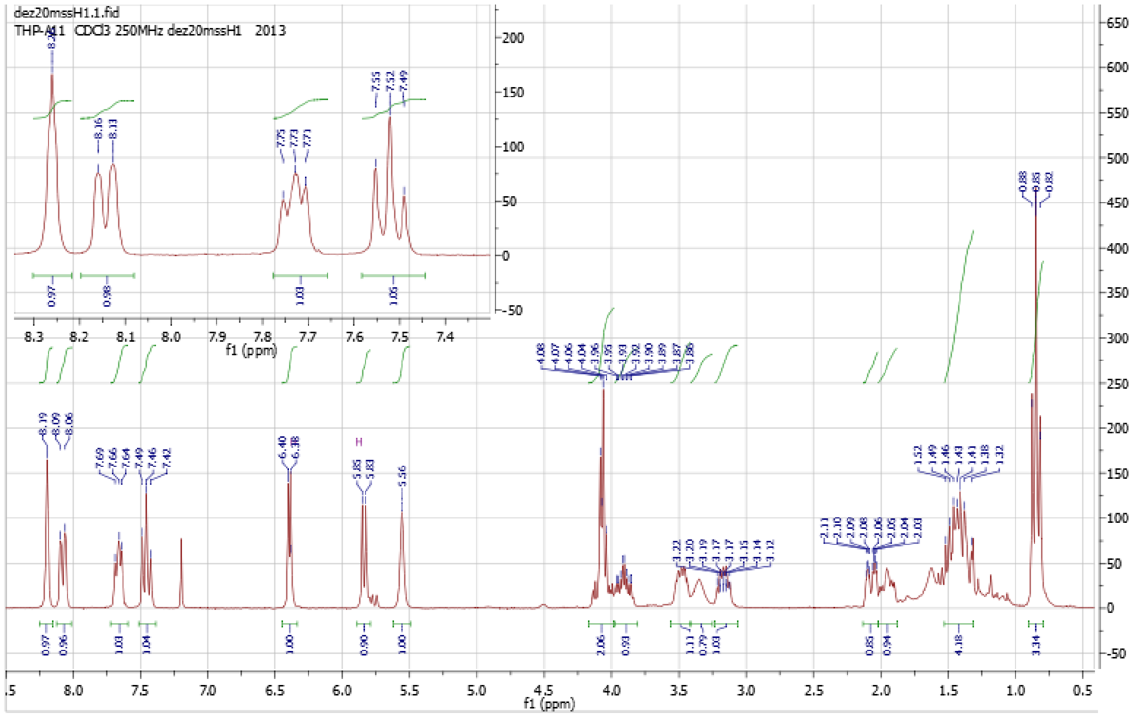

Figure S68. RMN ^{1}H spectrum of adduct **9b**.

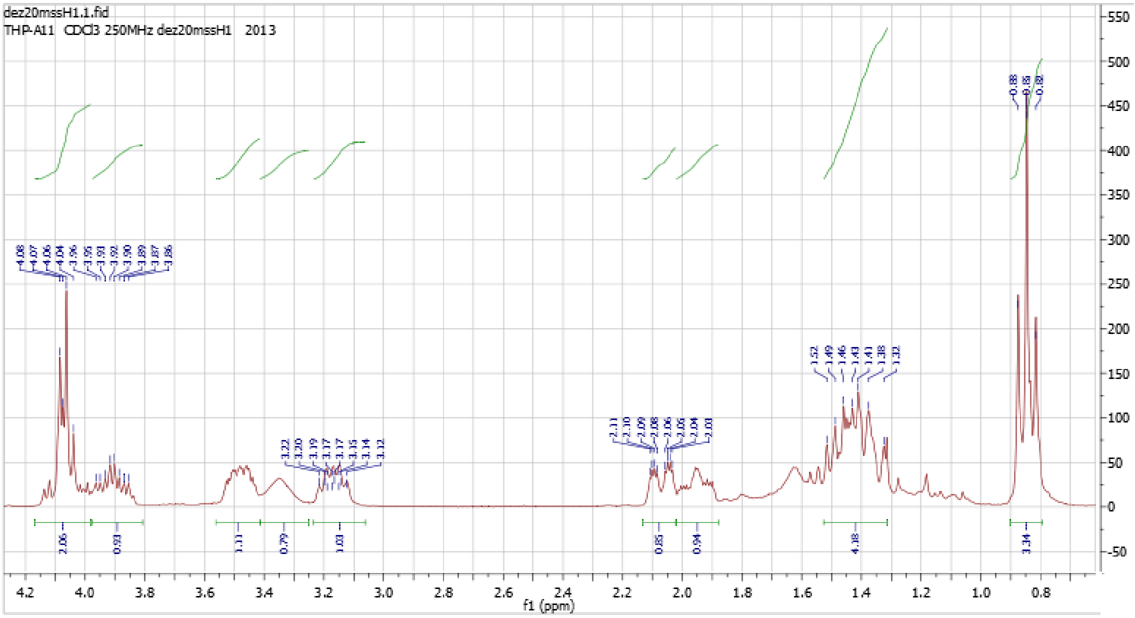

Figure S69. RMN ^{1}H spectrum enlargement of adduct **9b** (0.8 – 4.2ppm).

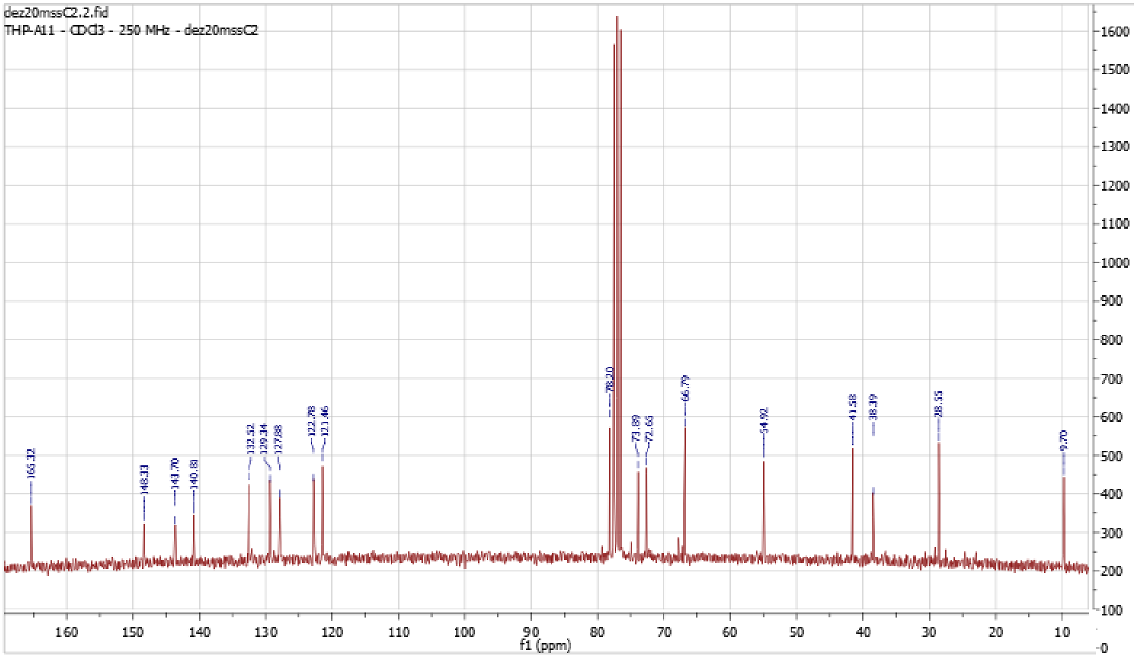

Figure S70. RMN ^{13}C spectrum of adduct **9b**.

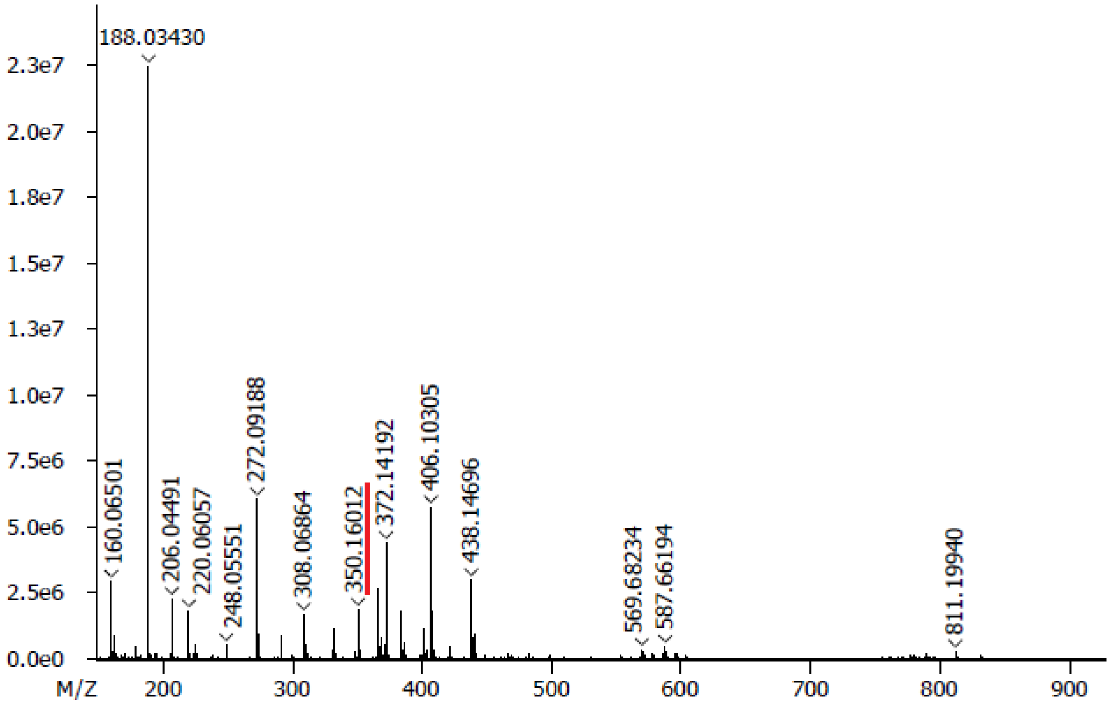

Figure S71. HRMS spectrum of compound **9b**.

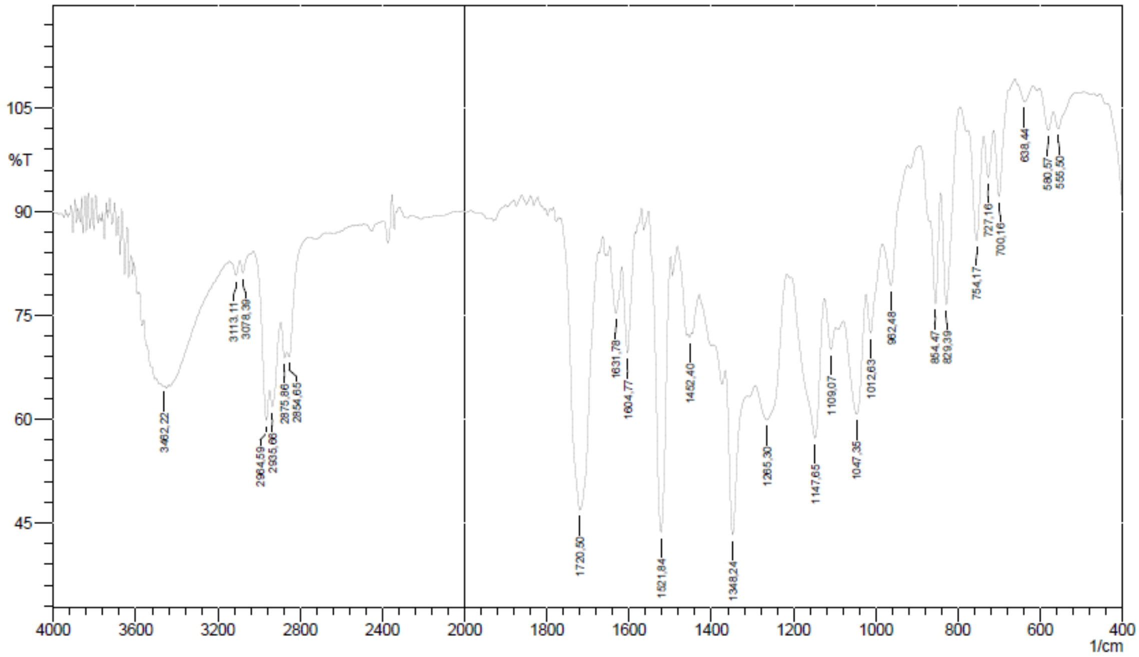

Figure S72. FTIR spectrum of adduct **9c**.

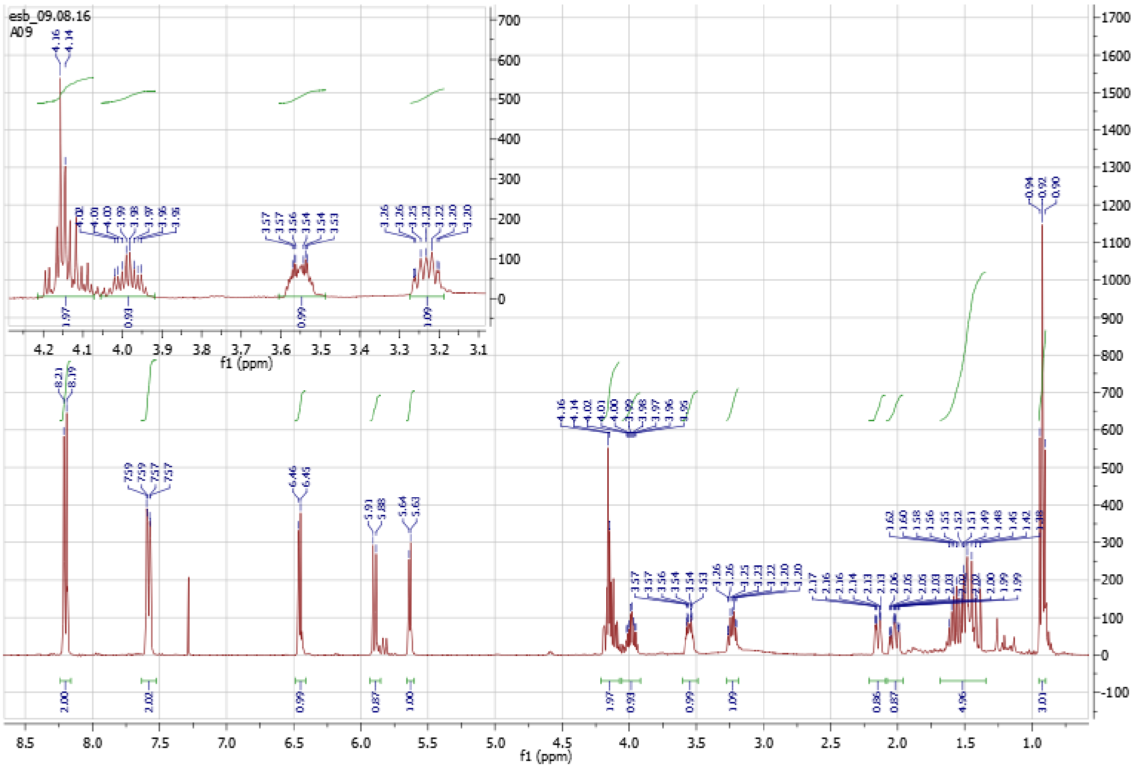

Figure S73. RMN ^{1}H spectrum of adduct **9c**.

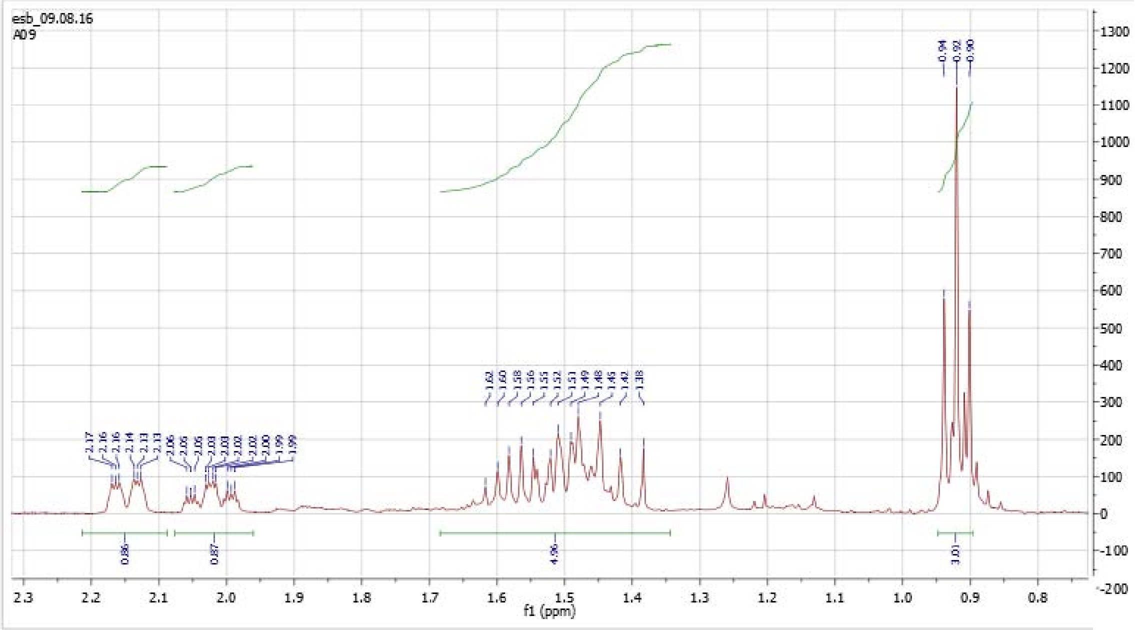

Figure S74. RMN ^{1}H spectrum enlargement of adduct **9c** (0.8 – 2.2ppm).

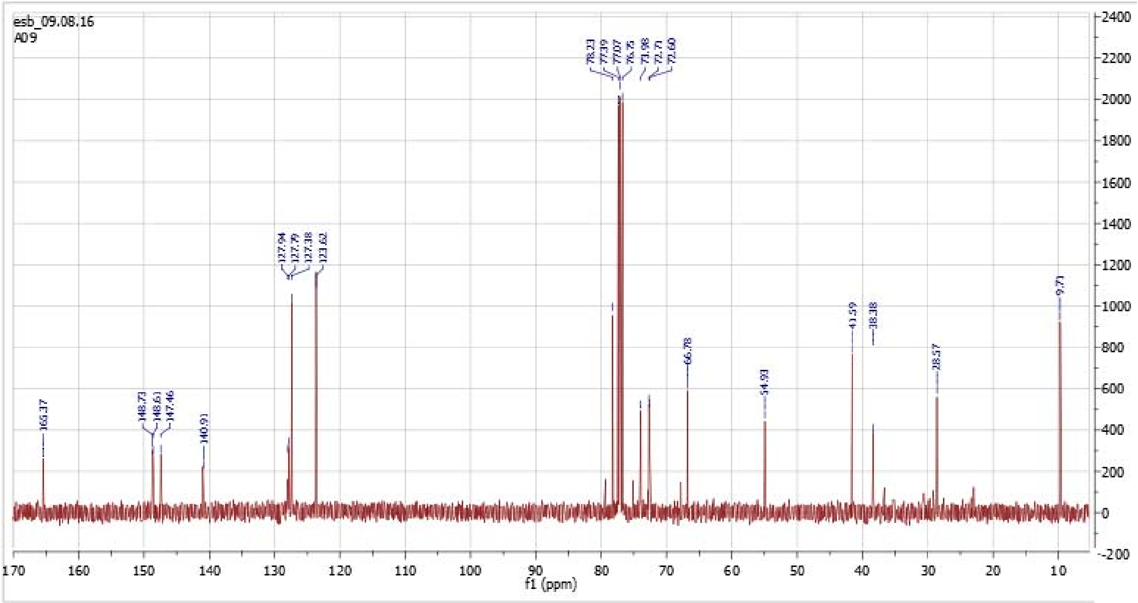

Figure S75. RMN ^{13}C spectrum of adduct **5c**.

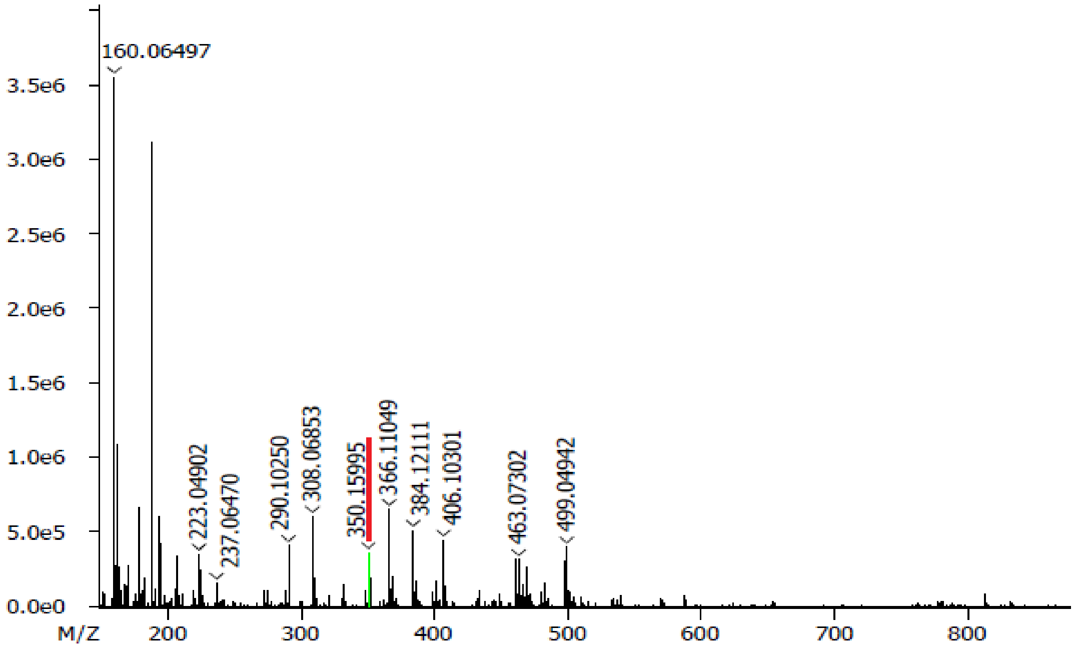

Figure S76. HRMS spectrum of compound **9c**.

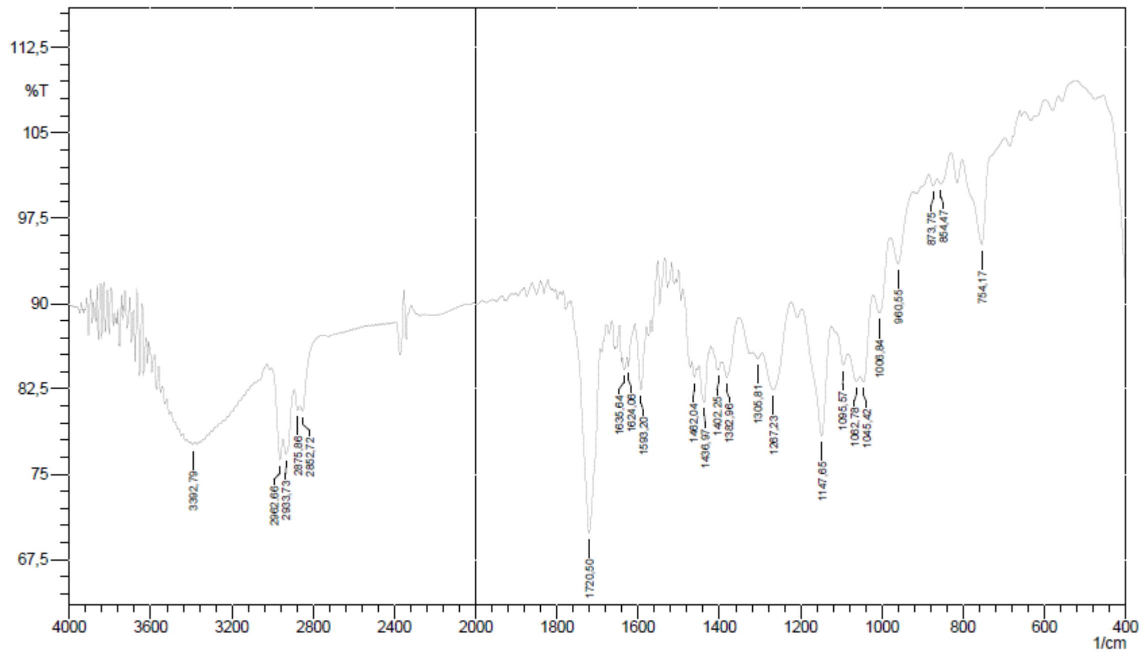

Figure S77. FTIR spectrum of adduct **9d**.

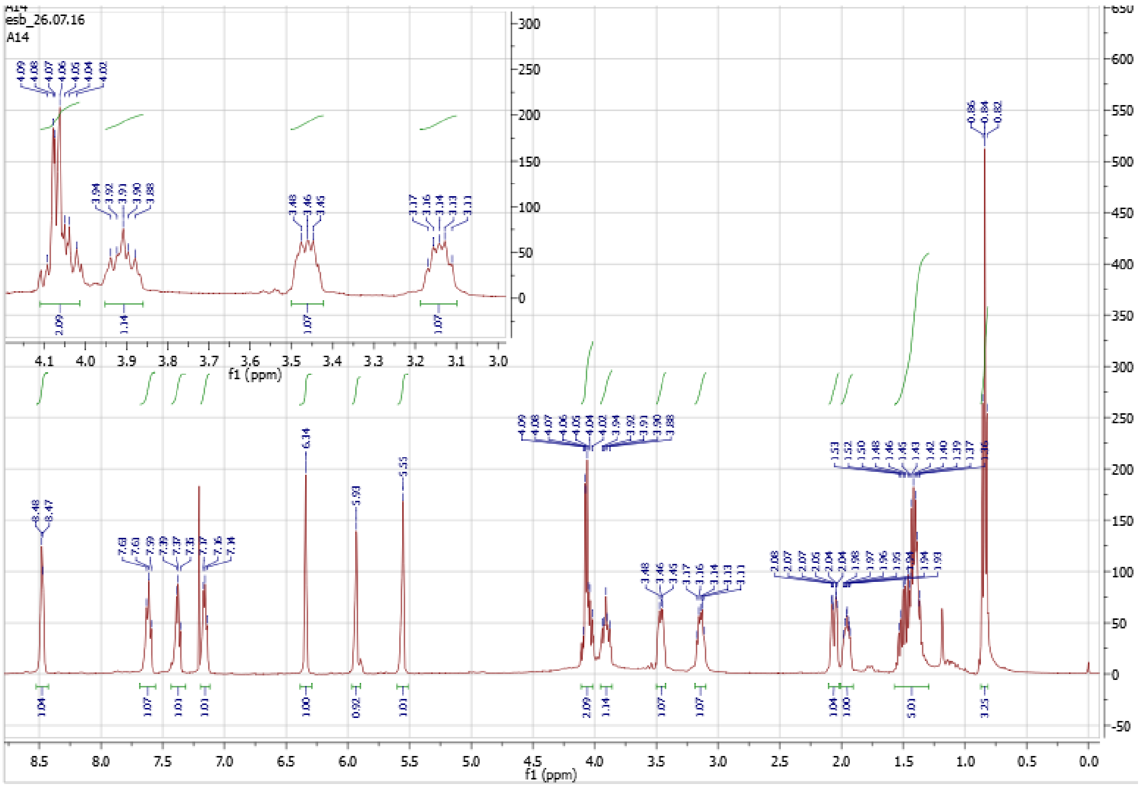

Figure S78. RMN ^{1}H spectrum of adduct **9d**.

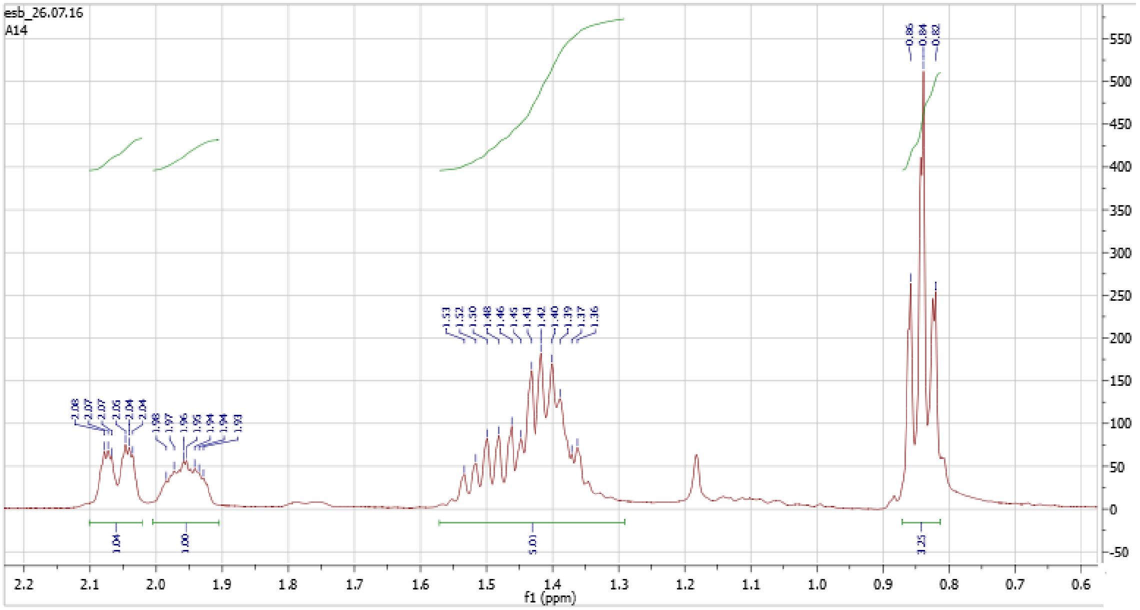

Figure S79. RMN ^{1}H spectrum enlargement of adduct **9d** (0.7 – 2.2ppm).

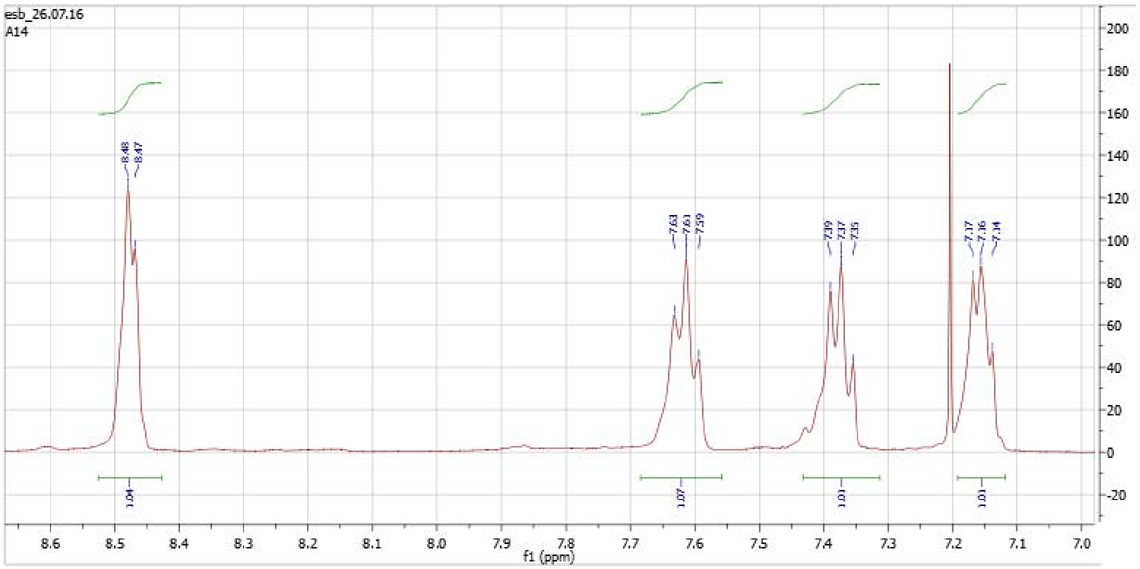

Figure S80. RMN ^{1}H spectrum enlargement of adduct **9d** (7.1 – 8.6ppm).

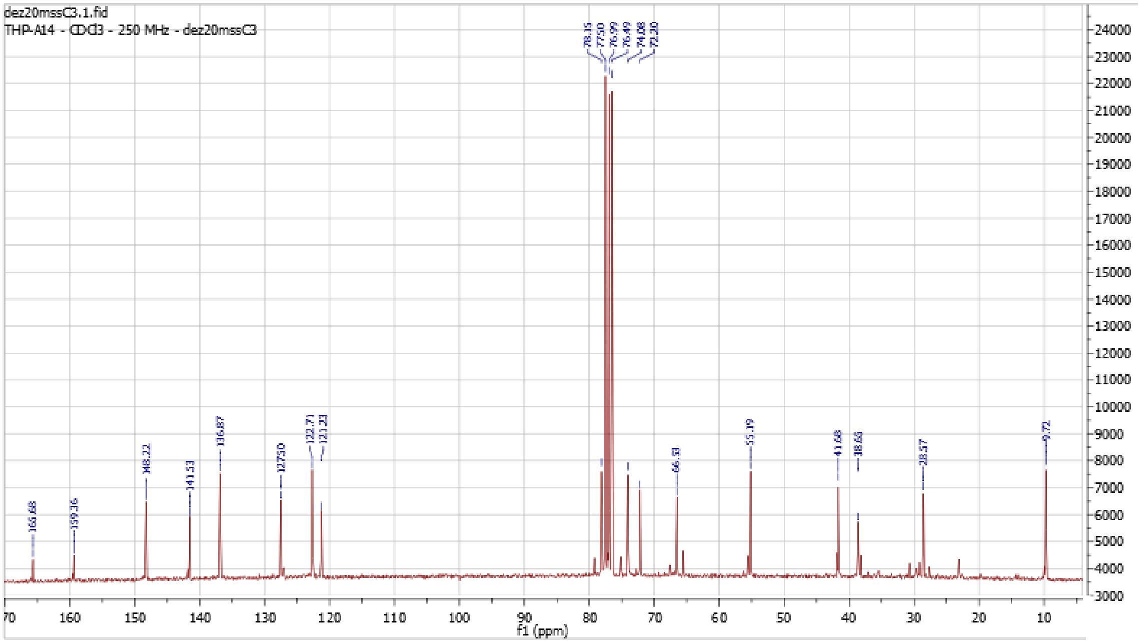

Figure S81. RMN ^{13}C spectrum of adduct **9d**.

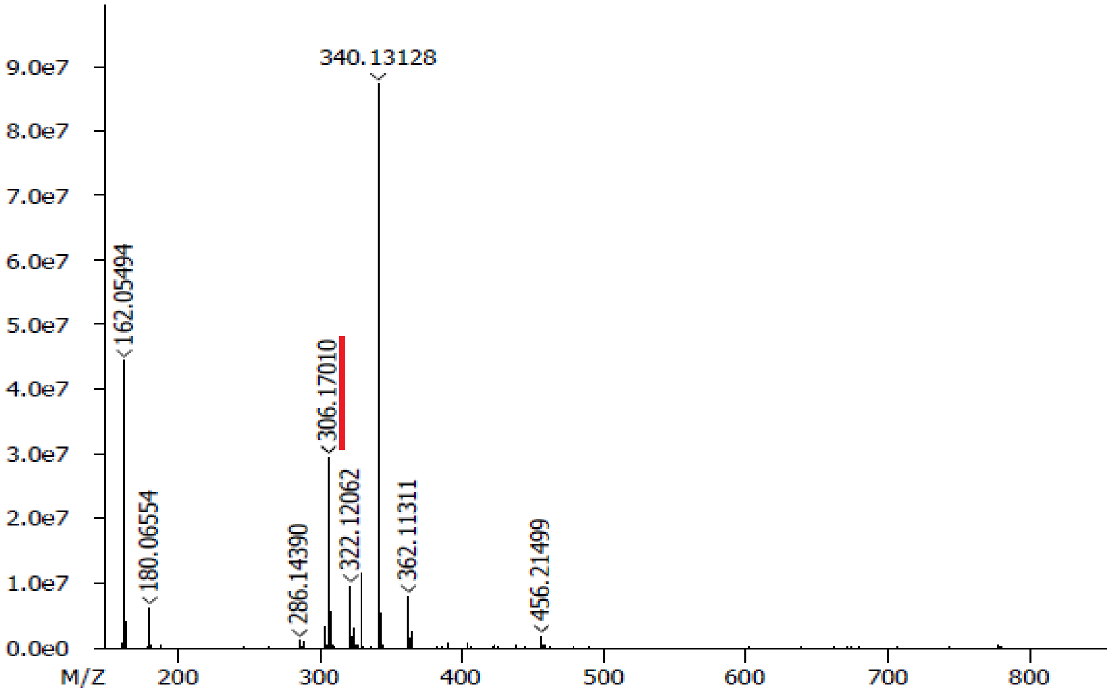

Figure S82. HRMS spectrum of compound **9d**.

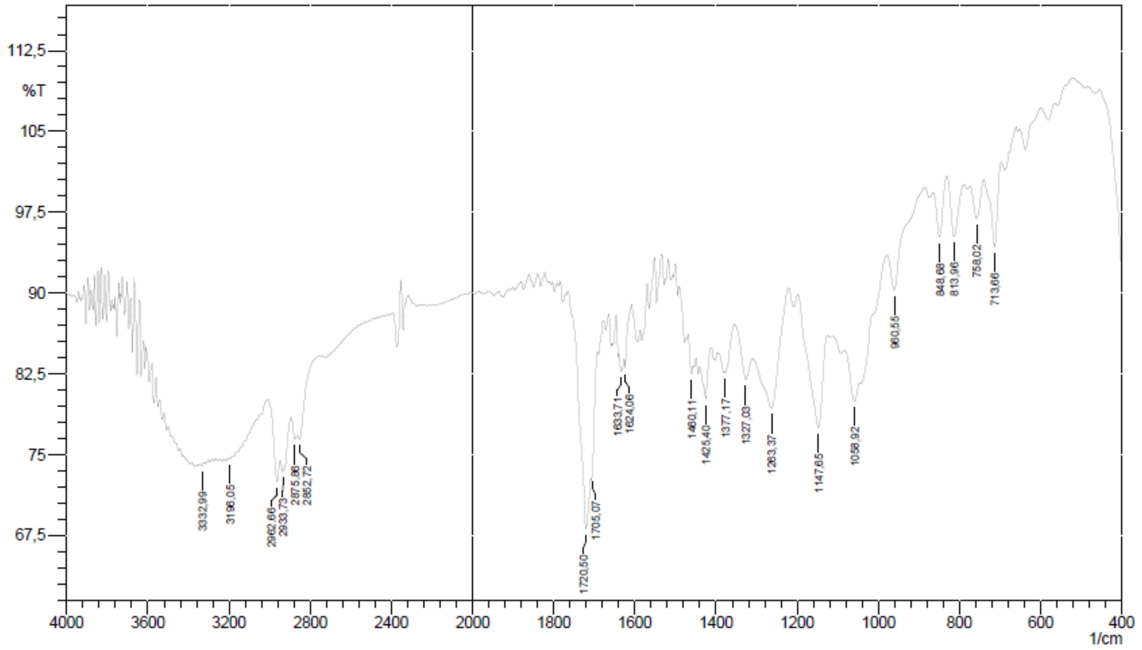

Figure S83. FTIR spectrum of adduct **9e**.

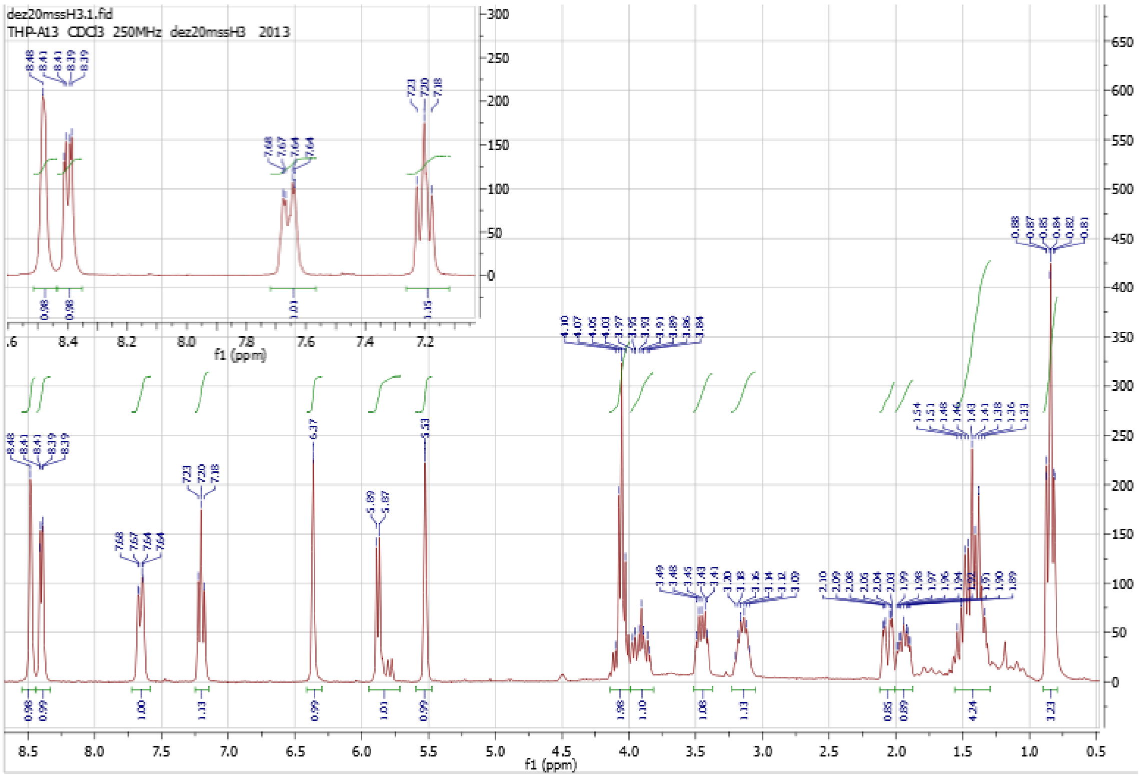

Figure S84. RMN ^{1}H spectrum of adduct **9e**.

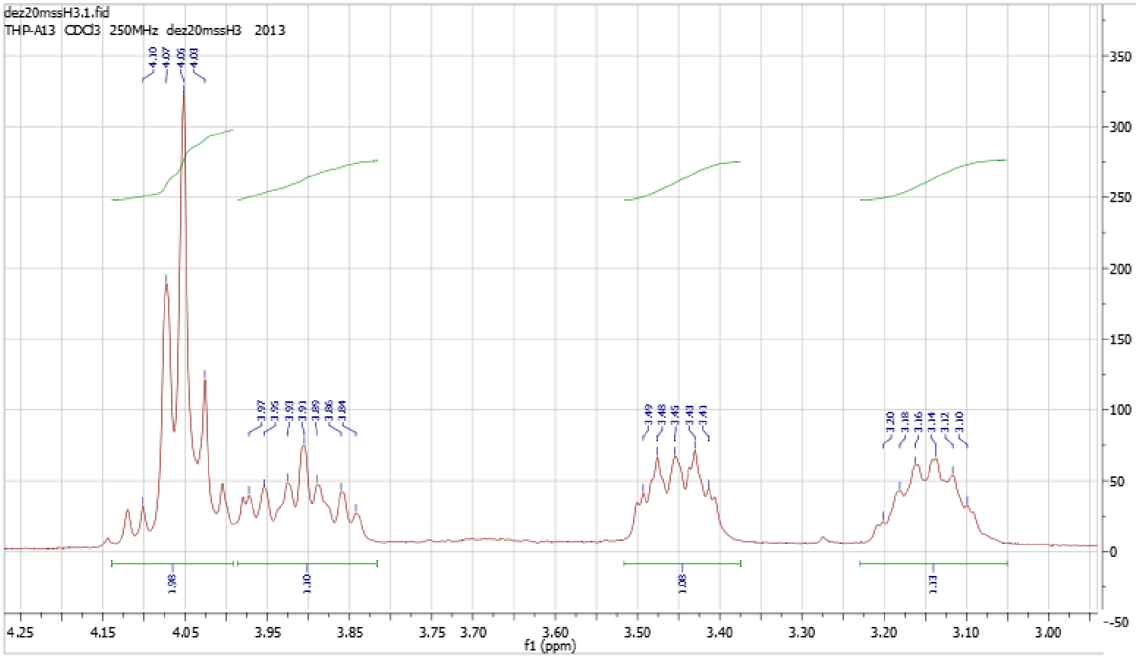

Figure S85. RMN ^{1}H spectrum enlargement of adduct **9e** (3.0 – 4.15ppm).

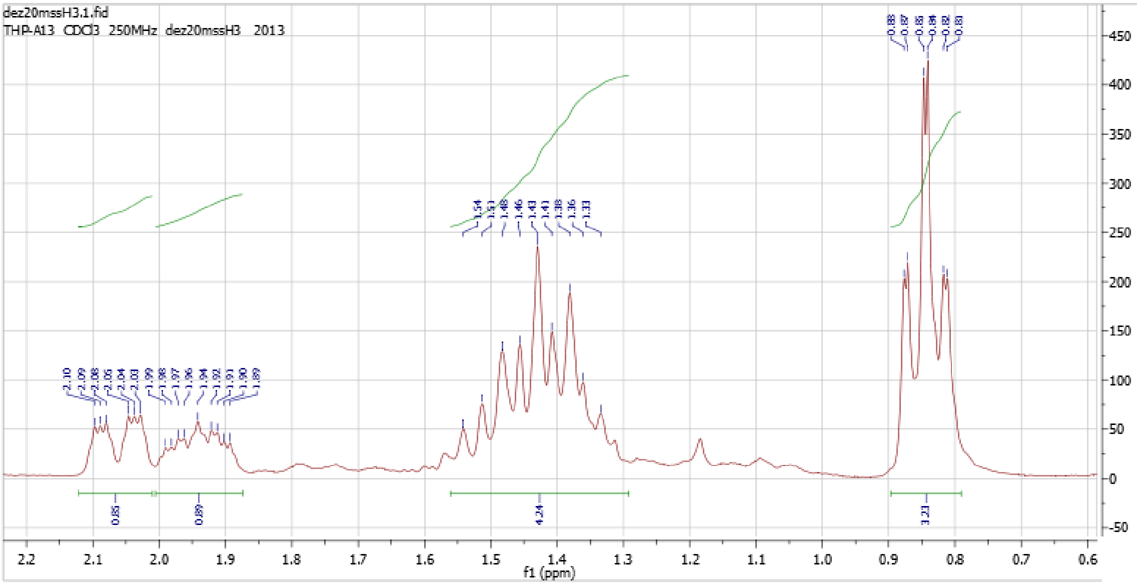

Figure S86. RMN ^{1}H spectrum enlargement of adduct **9e** (0.7 – 2.2ppm).

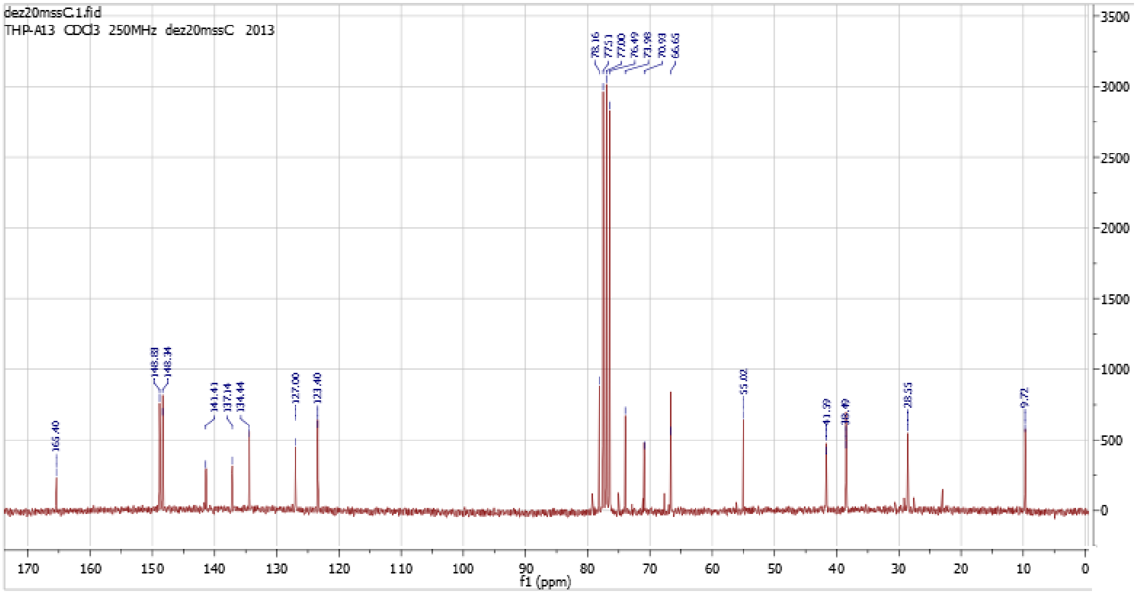

Figure S87. RMN ^{13}C spectrum of adduct **9e**.

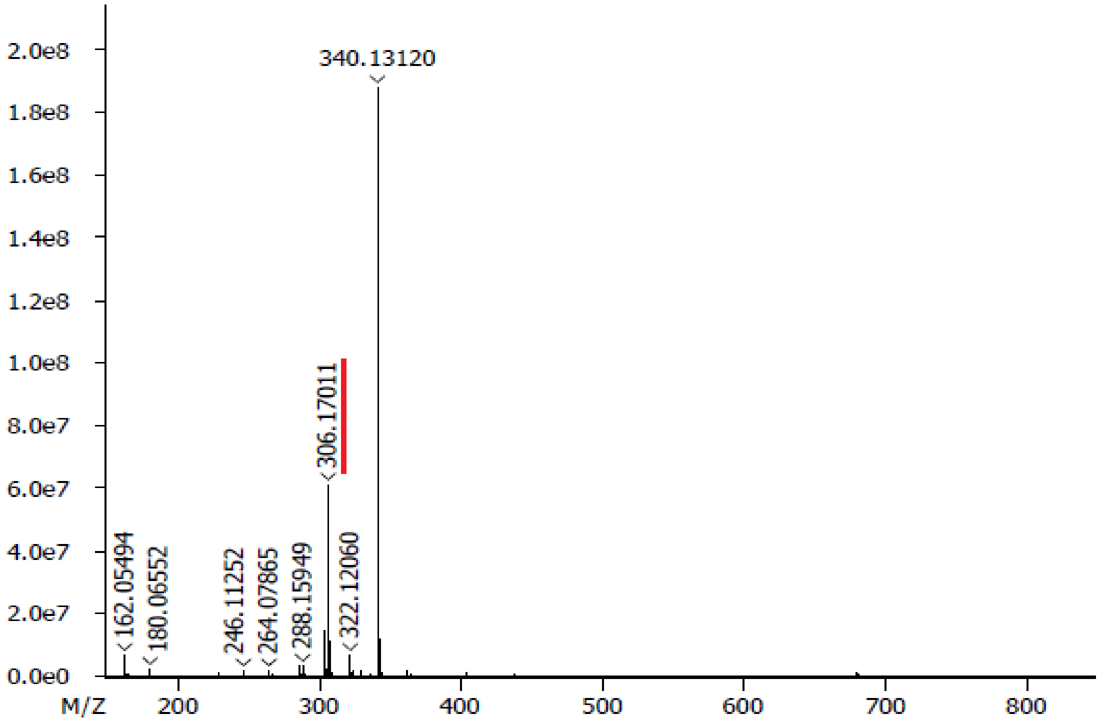

Figure S88. HRMS spectrum of compound **9e**.

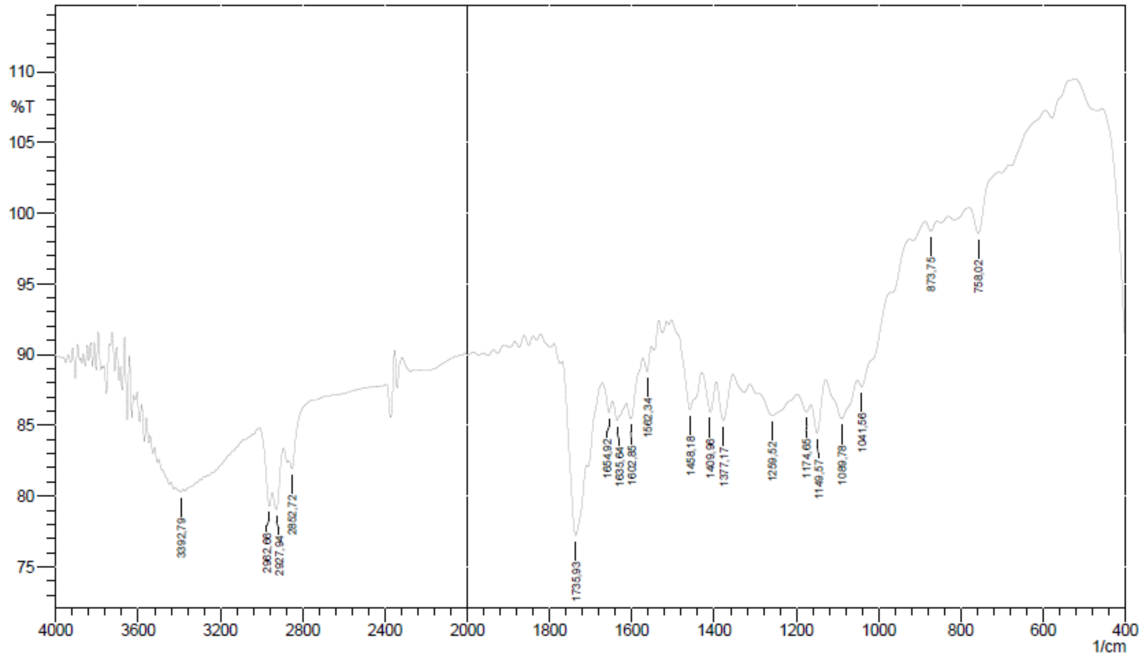

Figure S89. FTIR spectrum of adduct **9f**.

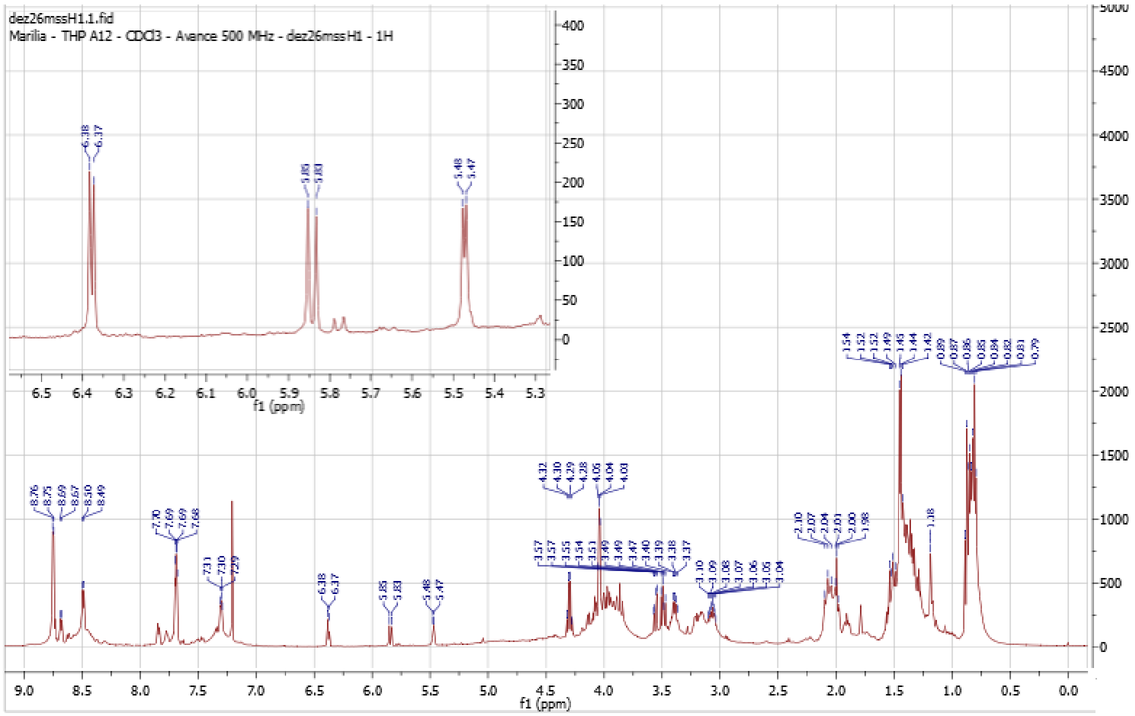

Figure S90. RMN ^{1}H spectrum of adduct **9f**.

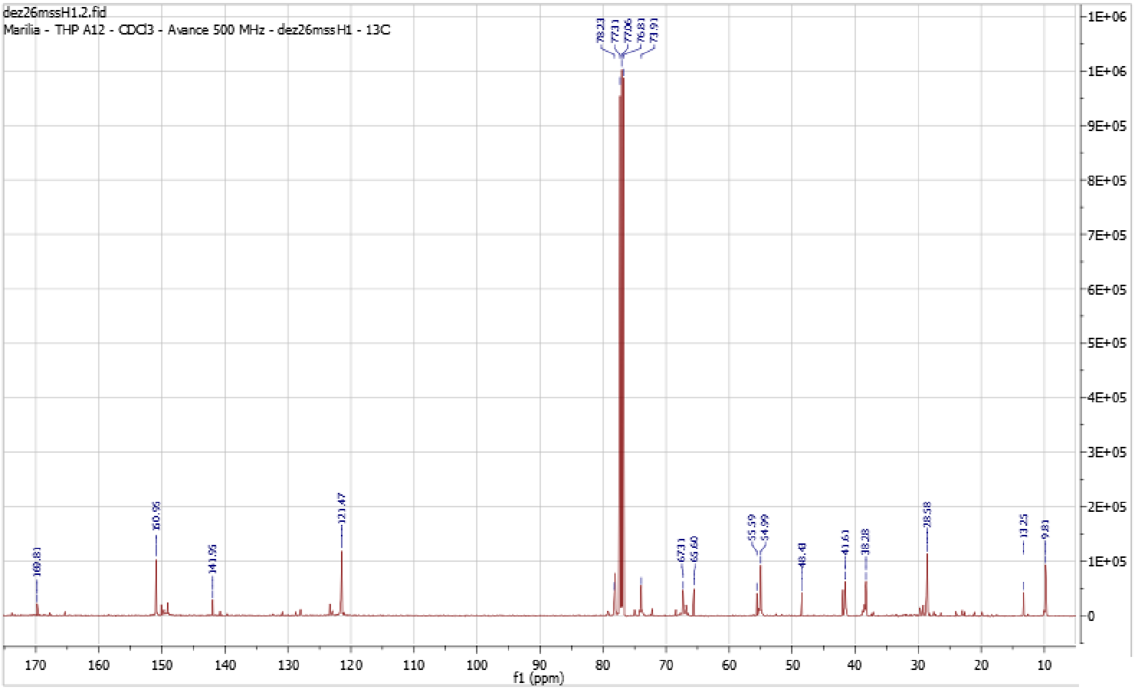

Figure S91. RMN ^{13}C spectrum of adduct **9f**.

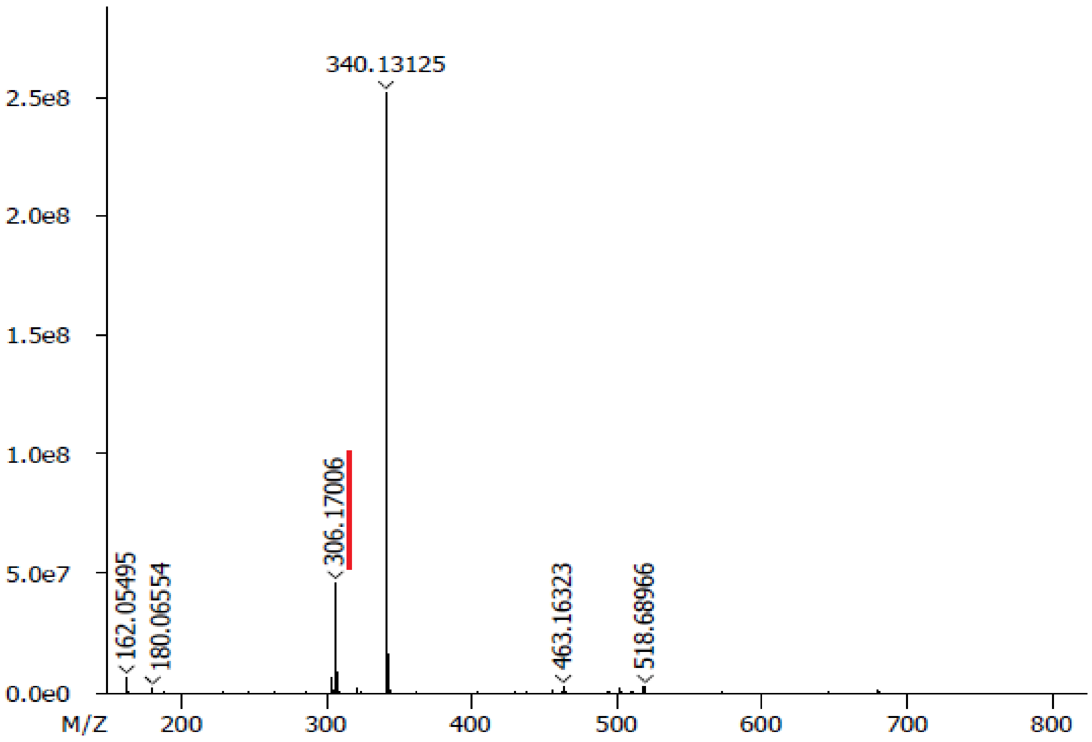

Figure S92. HRMS spectrum of compound **9f**.

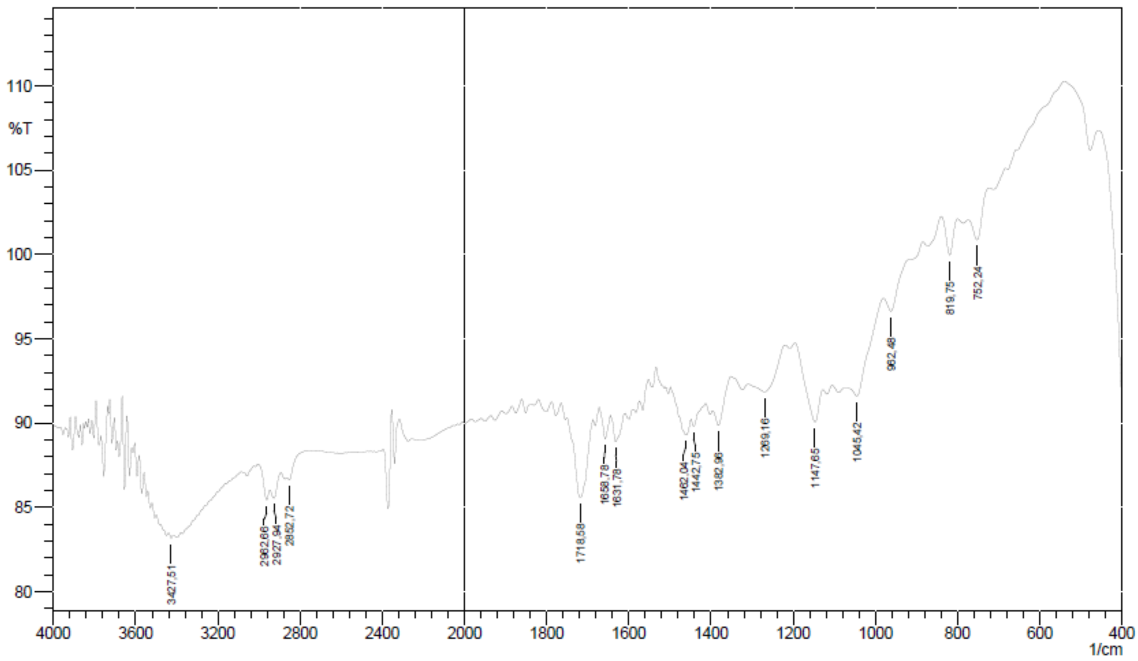

Figure S93. FTIR spectrum of adduct **9g**.

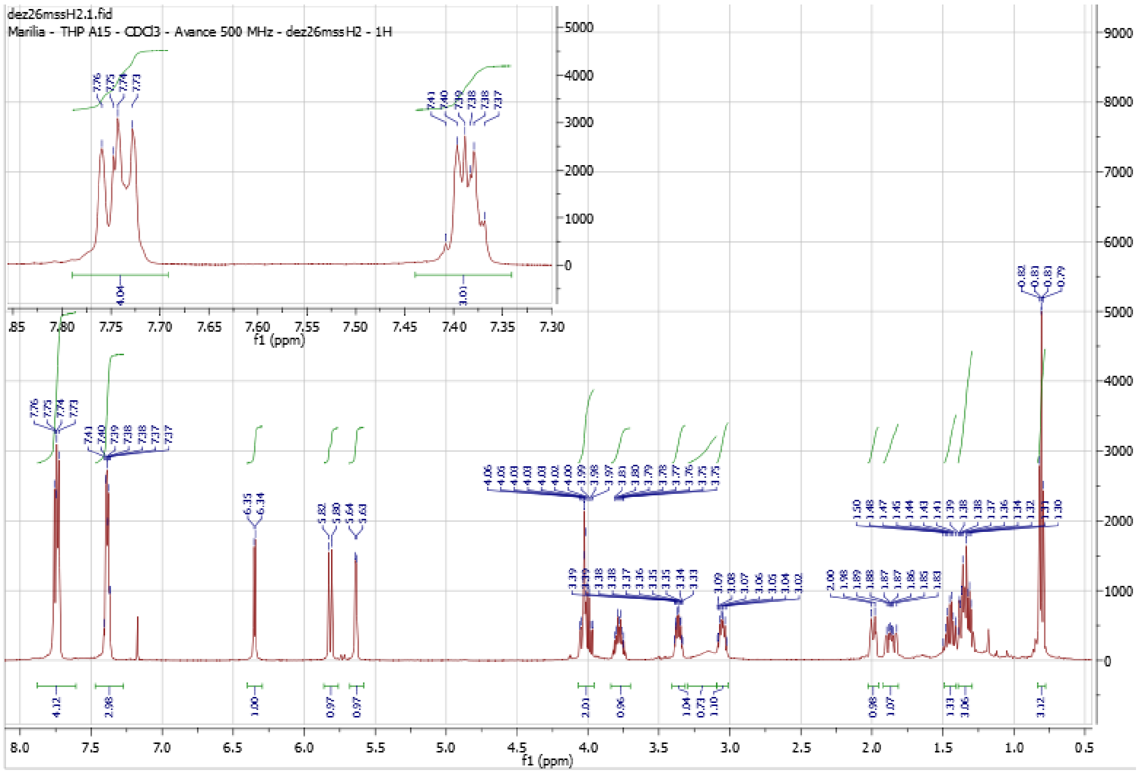

Figure S94. RMN ^{1}H spectrum of adduct **9g**.

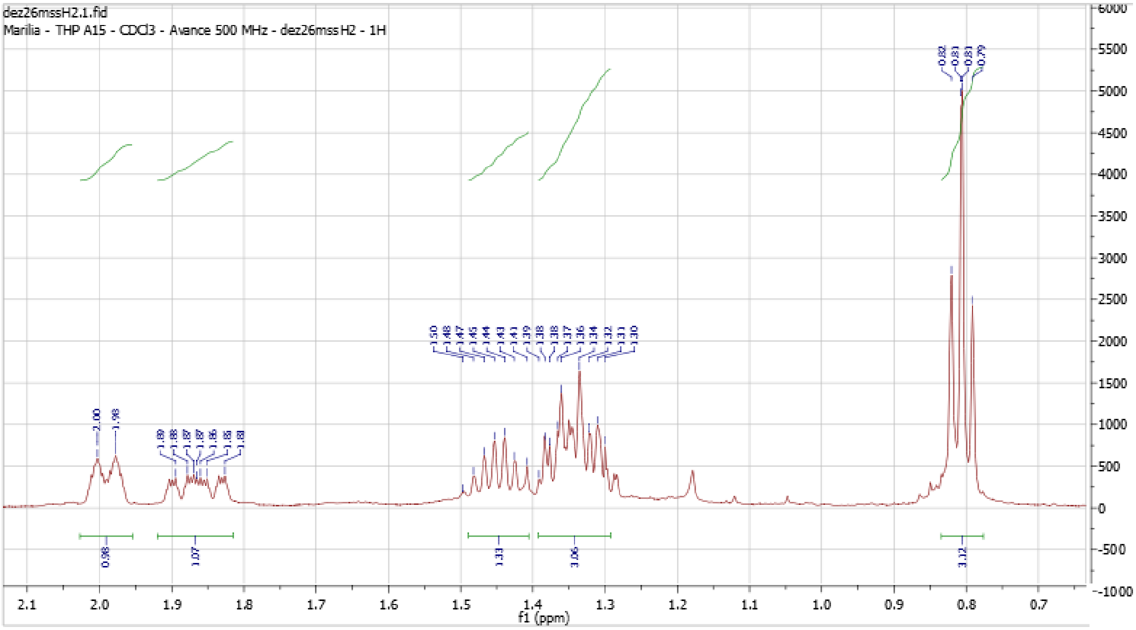

Figure S95. RMN ^{1}H spectrum enlargement of adduct **9g** (0.7 – 2.1ppm).

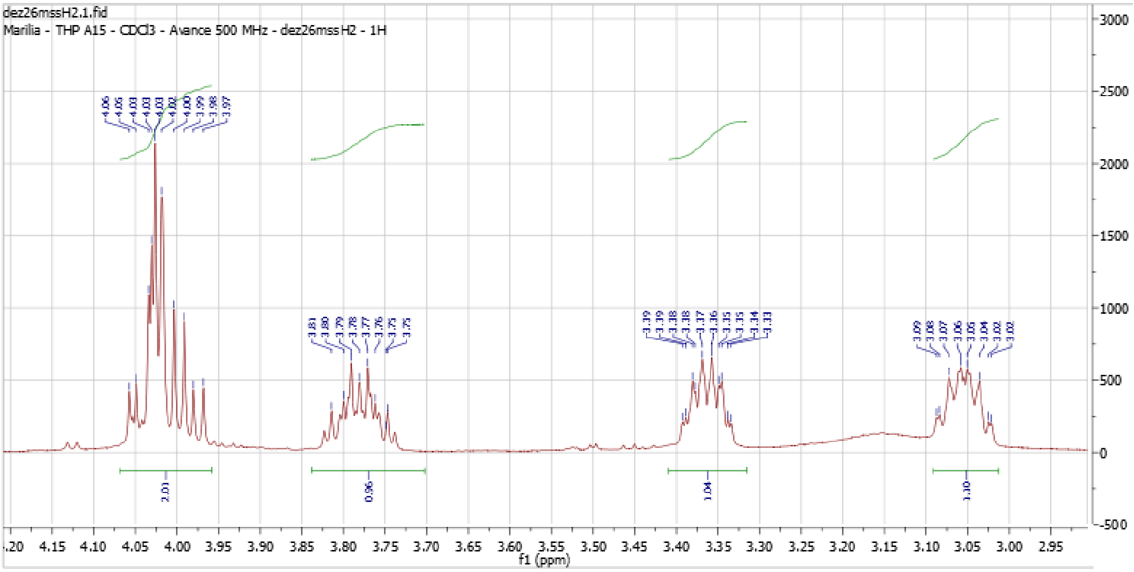

Figure S96. RMN ^{1}H spectrum enlargement of adduct **9g** (3.0 – 4.1ppm).

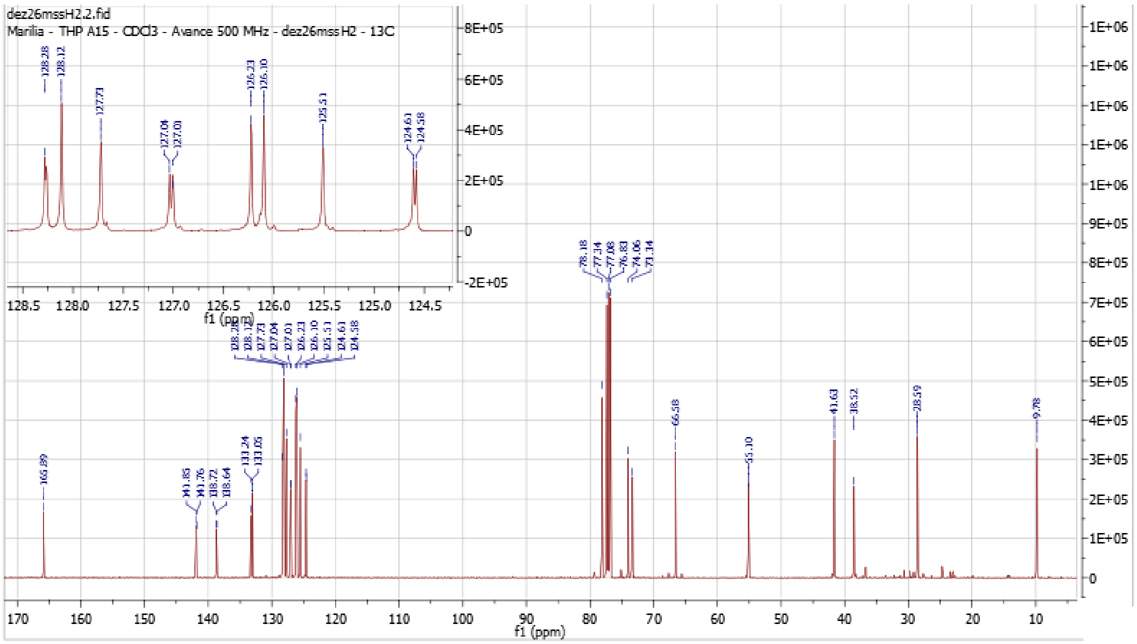

Figure S97. RMN ^{13}C spectrum of adduct **9g**.

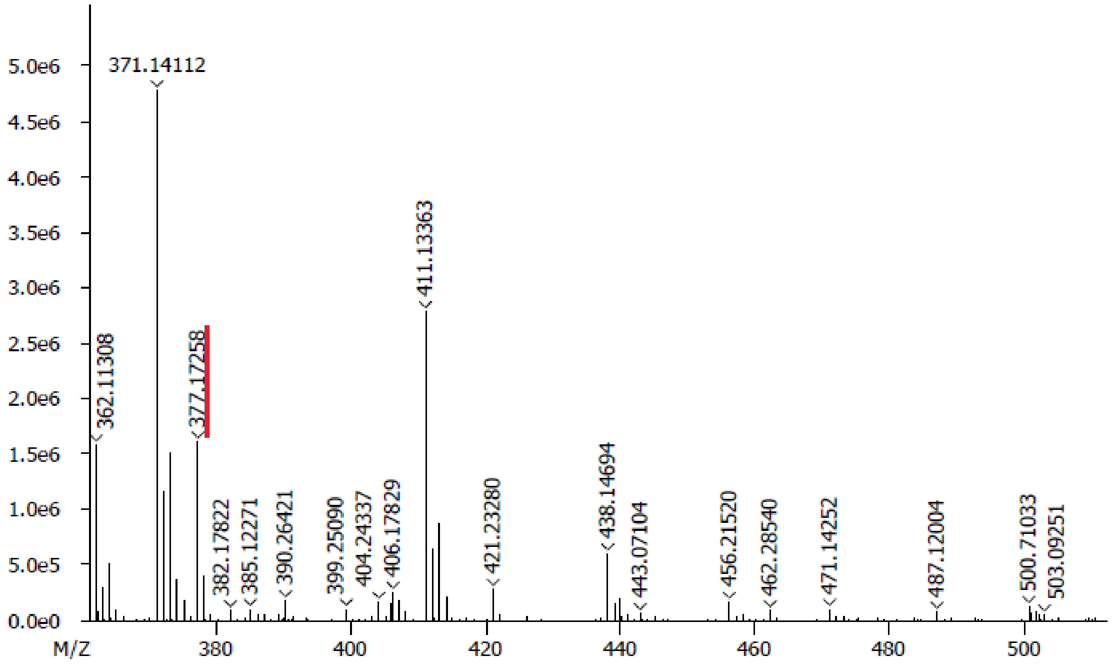

Figure S98. HRMS spectrum of compound **9g**.

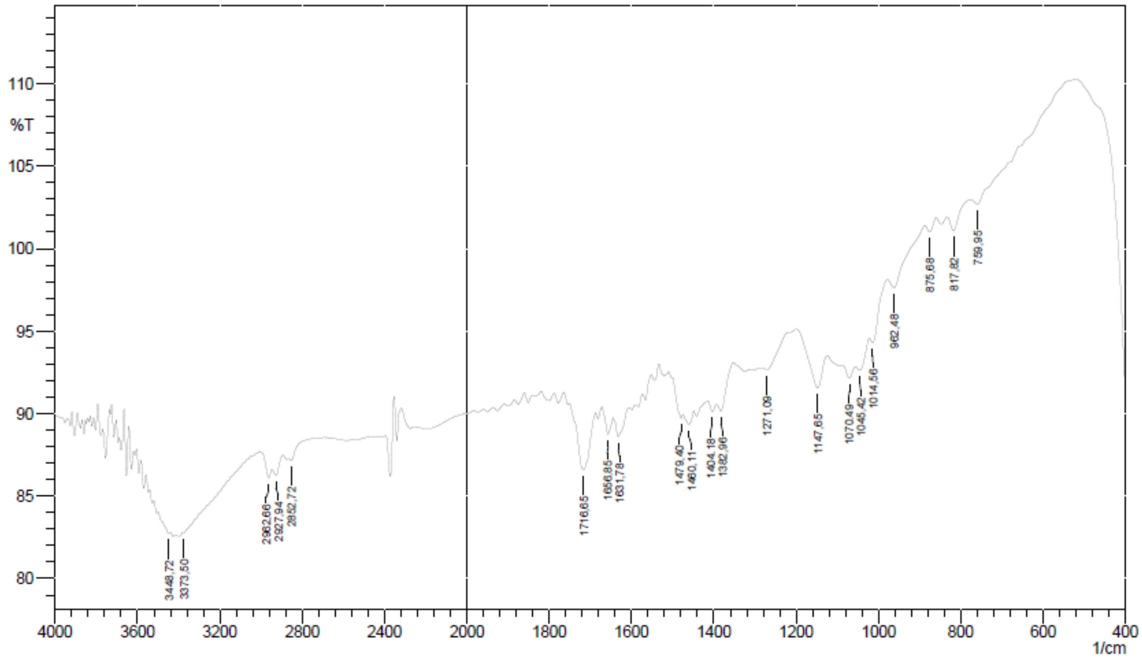

Figure S99. FTIR spectrum of adduct **9h**.

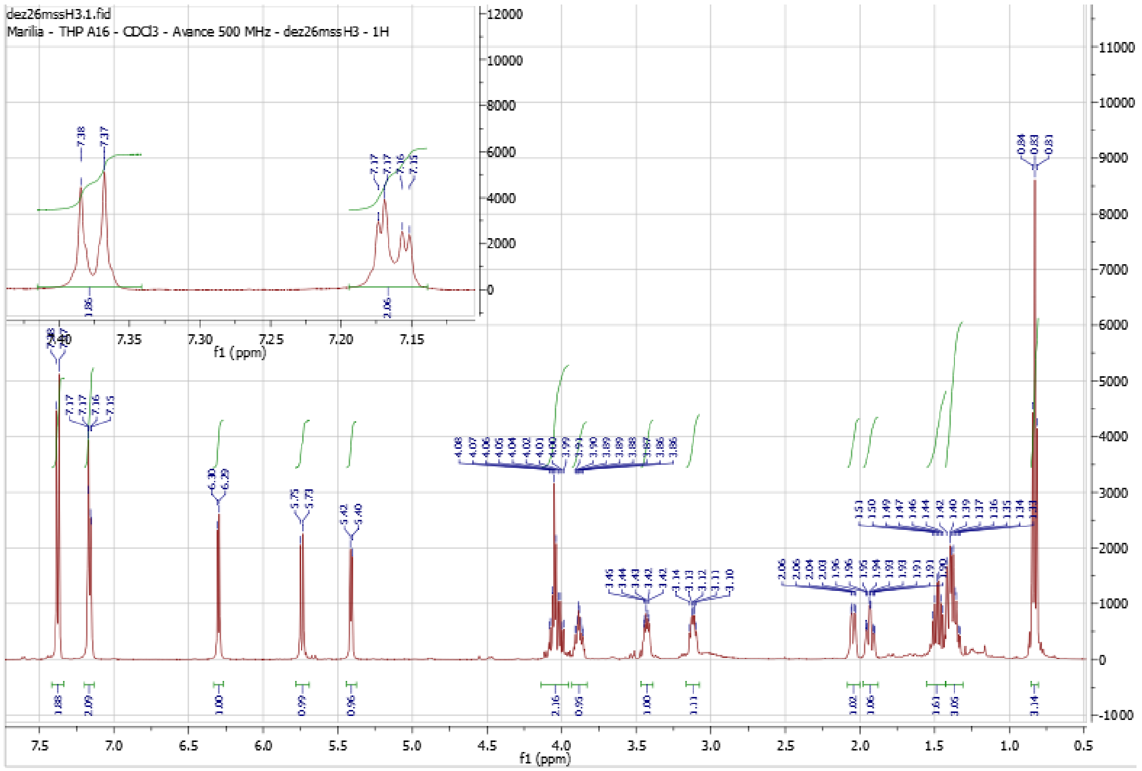

Figure S100. RMN ^{1}H spectrum of adduct **9h**.

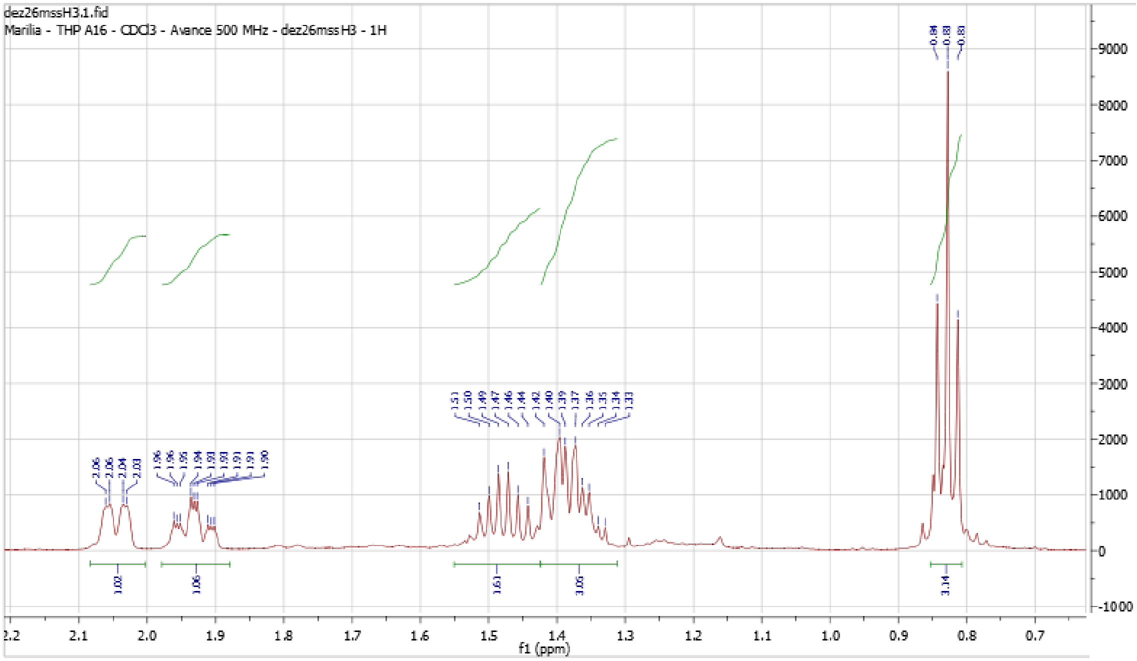

Figure S101. RMN ^{1}H spectrum enlargement of adduct **9h** (0.7 – 2.1ppm).

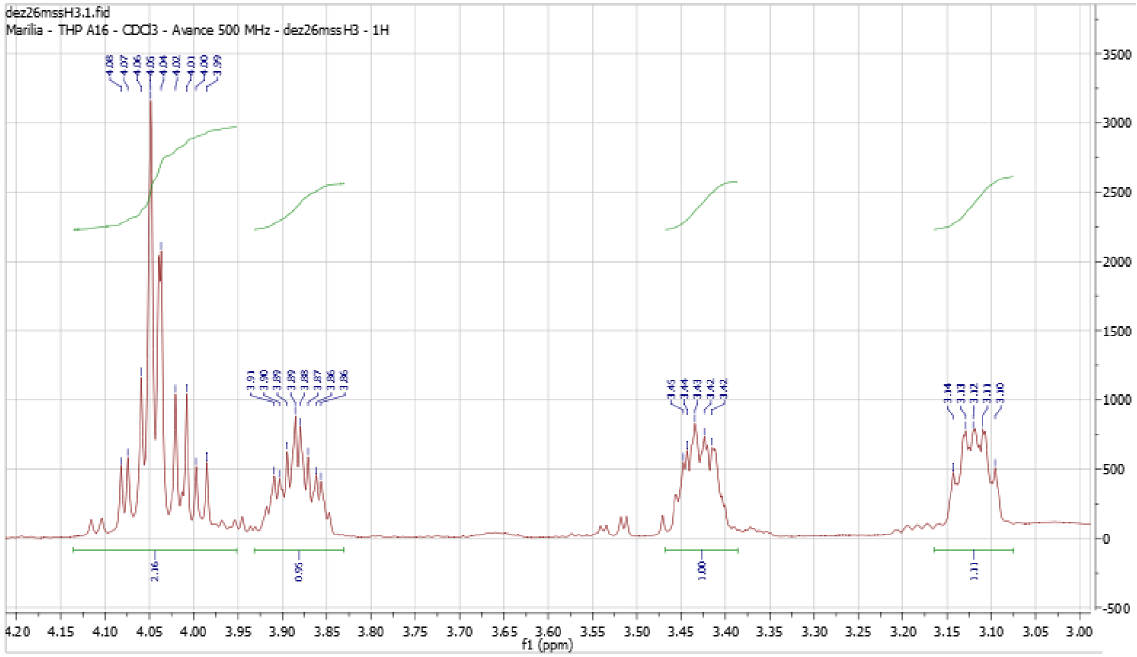

Figure S102. RMN ^{1}H spectrum enlargement of adduct **9h** (3.05 – 4.15ppm).

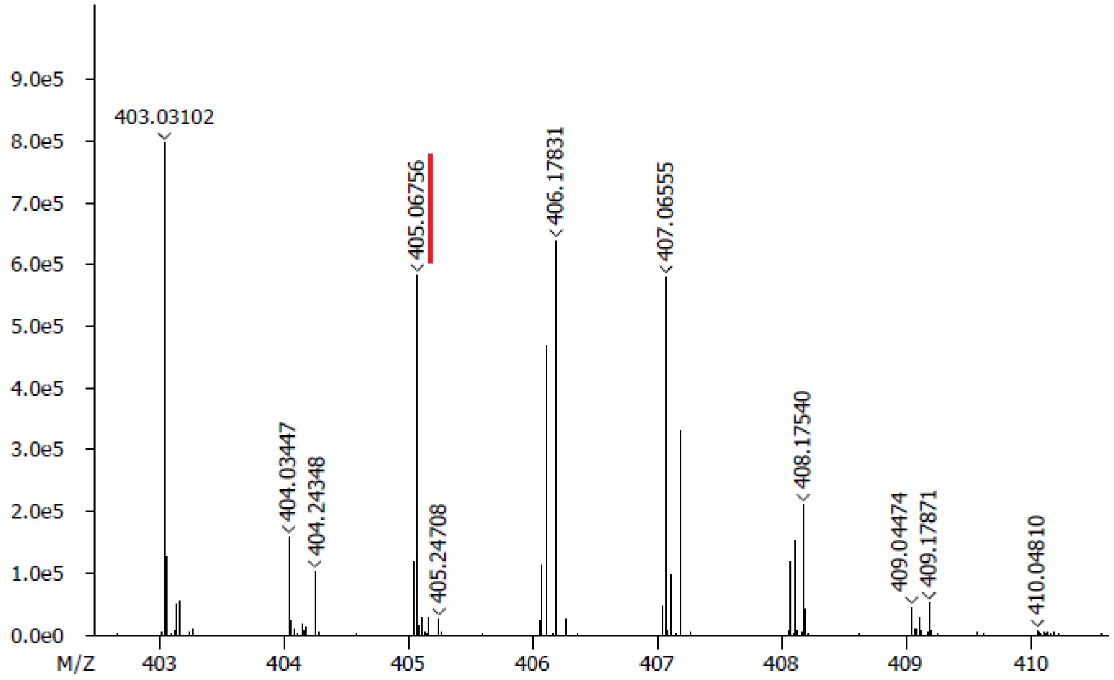

Figure S 103. HRMS spectrum of compound **9h**.
